# Immunomodulatory contribution of mast cells to the regenerative biomaterial microenvironment

**DOI:** 10.1038/s41536-023-00324-0

**Published:** 2023-09-20

**Authors:** Raymond M. Wang, Joshua M. Mesfin, Maria Karkanitsa, Jessica L. Ungerleider, Emma Zelus, Yuxue Zhang, Yu Kawakami, Yuko Kawakami, Toshiaki Kawakami, Karen L. Christman

**Affiliations:** 1 Shu Chien-Gene Lay Department of Bioengineering, Sanford Consortium of Regenerative Medicine, University of California San Diego, 2880 Torrey Pines Scenic Drive, La Jolla, CA 92037 USA; 2 Department of Genitourinary Medical Oncology, The University of Texas MD Anderson Cancer Center, Houston, TX 77030 USA; 3 Laboratory of Allergic Diseases, Center for Autoimmunity and Inflammation, La Jolla Institute for Immunology, La Jolla, California 92037 USA; 4 Department of Dermatology, University of California San Diego, School of Medicine, La Jolla, CA 92093 USA

**Keywords:** Biomaterials, Regenerative medicine

## Abstract

Bioactive immunomodulatory biomaterials have shown promise for influencing the immune response to promote tissue repair and regeneration. Macrophages and T cells have been associated with this response; however, other immune cell types have been traditionally overlooked. In this study, we investigated the role of mast cells in the regulation of the immune response to decellularized biomaterial scaffolds using a subcutaneous implant model. In mast cell-deficient mice, there was dysregulation of the expected M1 to M2 macrophage transition typically induced by the biomaterial scaffold. Polarization progression deviated in a sex-specific manner with an early transition to an M2 profile in female mice, while the male response was unable to properly transition past a pro-inflammatory M1 state. Both were reversed with adoptive mast cell transfer. Further investigation of the later-stage immune response in male mice determined a greater sustained pro-inflammatory gene expression profile, including the IL-1 cytokine family, IL-6, alarmins, and chemokines. These results highlight mast cells as another important cell type that influences the immune response to pro-regenerative biomaterials.

## Introduction

The immune response is known to have a necessary role in tissue homeostasis, remodeling, and repair. Thus, enhancing our understanding of how immune cell populations direct these outcomes can improve our capacity to treat diseases and damaged tissues. Mast cells are both tissue-resident and infiltrating granulocyte populations classically associated with host defense, foreign body reactions, and various immune disorders^1^. These cells are often identified by their granules that contain a host of immune mediators contributing to pathological responses such as allergic reactions^2^, inflammatory cell recruitment^3^, and fibrosis^4^. Among diseased conditions, several studies have emphasized the detrimental role of mast cells in long-term tissue remodeling outcomes^5–7^. However, research has also produced confounding results^8–11^ or evidence of their beneficial contributions to select cases of native tissue repair or inflammatory resolution^9,12–15^, suggesting a greater functional complexity. Despite this evidence and the plethora of pro-remodeling/anti-inflammatory cytokines that mast cells are known to secrete, their role in tissue regenerative therapies has not been studied.

Decellularized ECM scaffolds are a rapidly expanding field of biomaterials that are emerging as a viable clinical strategy for tissue repair and regeneration^16^. These materials, which are derived from tissue that is stripped of its cellular content to isolate the underlying ECM scaffold, have been shown to elicit a host of endogenous cellular responses, including induced immune cell phenotypes for supporting improved tissue outcomes^17–19^. The immunomodulatory influence from pro-regenerative biomaterials has often been characterized as an early pro-inflammatory response that transitions to a pro-remodeling phenotype. This dynamic pro-inflammatory to pro-remodeling transition has also been observed in cases of native tissue repair and regeneration versus pathological tissue remodeling outcomes such as chronic inflammation and fibrosis^20–22^. Although this pro-remodeling shift has been rigorously evaluated with macrophage^18,19,21,23^ and T cell populations^23,24^, the potential contribution of mast cells to the immunomodulatory effect of naturally derived biomaterials has been largely ignored and underinvestigated^25^.

In vitro culture of human mast cell lines has demonstrated that interaction with the decellularized extracellular matrix (ECM) microenvironment promotes mast cell differentiation, maturation, and viability compared to collagen controls^26^. However, in vivo studies have mainly just evaluated shifts in mast cell numbers associated with biomaterial implantation, though their general low numbers in tissue make it difficult to determine conclusive results from this approach^27,28^. Early studies with synthetic materials assessing their functional role have mainly found a classical activated mast cell phenotype highlighting contributions to foreign body reactions with early inflammatory cell infiltration^3^ and fibrous capsule formation^29^. These synthetic implant studies have further shown that inhibiting mast cell degranulation or presence limits these foreign body reactions^29,30^, which can improve outcomes and function of these devices, such as with subcutaneously implanted glucose monitors^31^. However, decellularized ECM biomaterials do not elicit a foreign body reaction (with a lack of fibrous capsule and lack of foreign body giant cells) if appropriately decellularized and processed^32–35^, and yet an increase in mast cells was observed with a decellularized ECM hydrogel^17^, suggesting the phenotype of mast cells could potentially be altered similar to pro-remodeling macrophages and T cells.

For our investigation, we utilized an injectable ECM hydrogel derived from decellularized porcine left ventricular tissue. This material was selected given its demonstrated efficacy in both small and large animal myocardial infarction models^17,33,36^, immunomodulatory capability^17,23^, and biocompatibility^33^. Furthermore, biocompatibility was demonstrated with human immune cells in vivo in a humanized mouse model implantation study^23^ as well as through initial safety evaluation in a Phase I clinical trial in post-myocardial infarction patients^37^. For elucidating the response elicited by mast cells with decellularized biomaterials, mast cell-deficient Kit^W-sh^ mice were employed as they are highly deficient in mast cells across various tissues and in the skin by 10–12 weeks of age while maintaining relatively normal levels of a broad range of lymphocyte and myeloid lineages^38^. Responses in mast cell-deficient mice were compared to wild-type controls and deficient mice restored with mast cell engraftment to evaluate the contribution of mast cells to the immune response following biomaterial implantation. Based on recent evidence suggesting that mast cells can have beneficial influences on native tissue remodeling outcomes and their commonly cited role in mediating immune responses, we hypothesized that mast cells contribute to the immunomodulatory response of decellularized ECM biomaterial scaffolds. In this study, we demonstrated their influence on the pro-inflammatory to pro-remodeling immunomodulatory transition, which deviated in a sex-specific manner.

## Results

### Mast cell recruitment and degranulation in response to ECM biomaterials

In our initial investigation, we assessed how conventional host responses and immune reactions driven by mast cells were altered by the implantation of a decellularized biomaterial. Limited studies have investigated the interaction of infiltrating mast cells and naturally derived biomaterials in the in vivo setting aside from observation of increased recruitment^17,28^. For this study, a subcutaneous implant model was chosen as we and others have previously shown this can recapitulate the shifts in immune cell polarization induced by decellularized ECM materials in disease and injury models^23,39^, and is representative of long-term remodeling outcomes^20,34,39,40^.

**Fig. 1:**
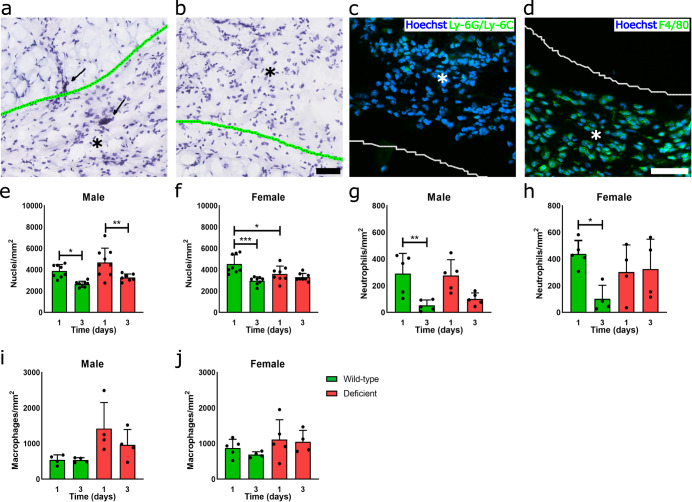
ECM scaffold promotes early immune cell infiltration with or without mast cells. Representative images of toluidine blue staining of ECM scaffold (green outlined with asterisk where material is present) and neighboring dermal tissue in wild-type (**a**) and mast cell-deficient (**b**) mice at day 1 post-injection. Mast cells can be found as early as day 1, showing a lack of or minimal degranulation (black arrow). Representative fluorescent images of injected biomaterial (white outline with asterisk denotes where material is present) with Ly-6G/Ly-6C^+^ neutrophil (**c**) and F4/80^+^ pan-macrophage staining (**d**) in green and Hoechst nuclei counterstain in blue. Quantification of total nuclei (**e**, **f**), neutrophil (**g**, **h**) and macrophage (**i**, **j**) density at 1 and 3 days post-injection for male and female wild-type (green) and mast cell-deficient (red) mice. *n* = 7–9 per group for total nuclei, *n* = 4–5 per group for immune cell quantification. Data displayed as mean ± SD. Scale bars of 50 µm in toluidine blue (**a**, **b**) and fluorescent images (**c**, **d**). (*p < 0.05, **p < 0.01, and ***p < 0.001).

Decellularized ECM hydrogels were subcutaneously injected into the dorsal region of C57BL6/J wild-type mice and Kit^W-sh^ mast cell-deficient mice. As mast cells have demonstrated sex-divergent responses in vitro^41^, in vivo^42^, and in mast cell-associated diseases clinically^43,44^, both male and female mice were initially assessed to determine whether this difference translated to altered biomaterial responses across sexes. Isolated subcutaneous injections showed mast cell presence at low cellular density as early as day 1 post-injection. Mast cells were non-degranulated or minimally degranulated based on toluidine blue staining (Fig. 1a). As expected, deficient mice showed no mast cell presence in the material or throughout the skin, confirming a lack of localized mast cell presence or recruitment (Fig. 1b). Greater inward infiltration of non-degranulated mast cells was observed in the ECM hydrogel (Supplementary Fig. 1a, b) by 3 days post-injection. Comparison with subcutaneous injection of a commercial milled urinary bladder matrix showed similar recruitment of non-degranulated mast cells bordering the material at day 3 post-injection, although there was a lack of inward infiltrating mast cells likely due to limited porosity compared to a hydrogel form (Supplementary Fig. 1c, d). In contrast, 3 days following injection of a nondegradable polyethylene glycol (PEG) hydrogel, a limited number of mast cells, which appeared partially degranulated, were found bordering the material (Supplementary Fig. 1e, f). There was also a lack of mast cells or any immune cell infiltration into the PEG hydrogel at this timepoint (Supplementary Fig. 1g, h). This suggested that the recruitment of mast cells that maintain a relatively non-degranulated state is specific to decellularized ECM materials and not due to the procedure or hydrogel implantation.

As mast cells and their degranulation are known as central mediators of foreign body reactions, the lack of significant degranulation despite an increased mast cell presence observed in our study and previous research^17^ aligns with the lack of foreign body reaction elicited with our ECM hydrogel and other decellularized materials^18,23,45,46^. Due to being a tissue-derived material, we also assessed for bacterial endotoxin content as these contaminates have been shown to influence mast cell response and the progression of foreign body reactions. A limulus amebocyte lysate (LAL) endotoxin assay measuring lipopolysaccharides determined 2.0 EU/mL in our ECM hydrogel. This suggests a low endotoxin content that was achieved under standard lab processing conditions versus more controlled conditions for a clinical product. This amount has been shown to be below concentrations that elicit pro-inflammatory responses due to bacterial contaminates in materials. Furthermore, LPS at several tenfold higher concentrations have been utilized for eliciting downstream anti-inflammatory responses that led to mitigation of foreign body reactions and were indicative of induced endotoxin tolerance^47–49^. Thus, the increased mast cell recruitment and limited of degranulation are likely not due to contaminates in our naturally derived material.

Similar to a previous study utilizing human mast cell lines to study the influence of decellularized ECM on mast cell viability^26^, interaction of differentiated bone marrow-derived FcƐRI^+^c-kit^+^ mast cells (Supplementary Fig. 2) with the ECM hydrogel material in solution at nongelling concentrations in comparison to collagen and media only controls showed higher alamarBlue^TM^ readings, confirming matrix interaction enhanced differentiated mast cell viability (Supplementary Fig. 3). All together, these results suggest that mast cell interaction with ECM biomaterials promoted an alternative phenotypic state instead of the classically activated degranulated phenotype found in allergic and foreign body type responses.

### Similar or increased leukocyte recruitment despite lack of mast cell contribution

One known physiological function of mast cells is the release of histamine during degranulation and secretion of chemokines for promoting vascular permeability and recruitment of immune cells such as neutrophils^50,51^ and macrophages^52^ to sites of inflammation or in response to foreign agents. Studies have utilized mast cell-deficient mice and agents to demonstrate the benefits of inhibiting this mast cell function with synthetic material implants^3,30^. However, pro-regenerative ECM biomaterials have been demonstrated to drive and rely on the infiltration of a variety of immune cell populations for promoting immunomodulatory responses and subsequent tissue repair^53^. Thus, we evaluated whether the early infiltrating mast cells we observed with our decellularized biomaterials were contributing to the recruitment of other immune cell populations.

Total cellular infiltration into the ECM hydrogel scaffold between deficient and wild-type mice was quantified along with staining for Ly-6G/Ly-6C^+^ neutrophil (Fig. 1c) and F4/80^+^ macrophage populations (Fig. 1d). Surprisingly, we observed that cellular recruitment was overall the same and sometimes higher without mast cell presence, contrasting results with synthetic materials. In male mice, similar nuclei density was observed between mouse models that were initially higher on day 1 and significantly decreased on day 3 with no significant difference between models at matching timepoints (Fig. 1e). In contrast, a higher total cell infiltration was observed in female mice at day 1 for wild-type compared to deficient mice, though this difference was lost by day 3 (Fig. 1f). Assessment of neutrophils notably showed a similar high- to low-density change pattern between day 1 to 3 (Fig. 1g), likely from the characteristic early neutrophil-driven immune response. This pattern was not seen with female mast cell-deficient mice, which maintained a higher average neutrophil density at the day 3 timepoint (Fig. 1h). Similarly, staining for total macrophages showed no significance between either sex or model, though averages were generally higher in the deficient model (Fig. 1e–j). Overall, these results demonstrated that early immune cell infiltration into implanted ECM biomaterials does not depend on mast cell contribution.

### Macrophage polarization is influenced by mast cell presence

Another mast cell function is the release of a varied secretome with components that are known to regulate various phases of the immune response. As ECM scaffold injection allowed for a similar early cellular infiltration in both mast cell-deficient and wild-type mice despite the lack of mast cell contribution (Fig. 1), it was investigated whether mast cells acted as a regulatory contributor to the expected pro-inflammatory M1 to pro-remodeling M2 macrophage polarization transition elicited from an ECM biomaterial^20–22^.

A comprehensive flow cytometry panel (Supplementary Figs. 4 and 5) was performed to evaluate the immune cell content in subcutaneously injected hydrogels. A comparison of relative macrophage polarization was determined by the M2/M1 macrophage ratio based on CD206 versus CD86 expression. For simplicity, only macrophages that were distinctly M2 as CD206^+^CD86^−^ or M1 CD206^−^CD86^+^ were used in this evaluation. Response to complex stimuli in vivo, such as from ECM scaffolds, and general improvements in high-throughput technology have improved our understanding of the variety of macrophage populations and phenotypes outside the classic M2 versus M1 polarization paradigm. These include macrophages with both CD206^+^CD86^+^ being expressed in vivo^14,45,53^ that have been suggested to influence ECM remodeling^54^, macrophages that remain double negative or silent despite the presence of a specific classical stimuli^55^, and other unique subpopulations discovered by single-cell technologies^19,55,56^. Indeed, based on this macrophage variety, several guidelines for in vitro studies have been proposed for distinguishing these phenotypes based on stimuli and detailing of experimental parameters^57^. However, with the greater complexity and variety in the in vivo and clinical setting, the M2 versus M1 approach has still maintained use for the generalized characterization of the predominant macrophage response. Specifically, it has been utilized as a prognosis marker in patients^20,58^ and for initial screening of a biomaterial’s response and safety^23,24,34,59^.

**Fig. 2:**
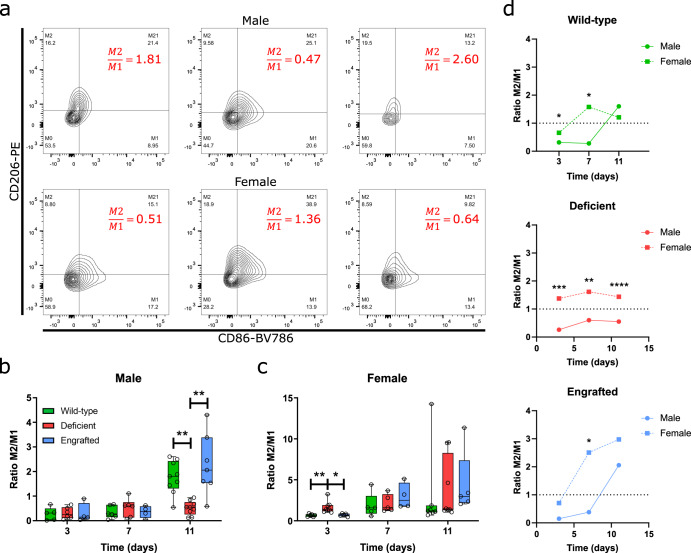
Transition from a pro-inflammatory to pro-remodeling macrophage is dysregulated in mast cell-deficient mice in a sex-specific manner. **a** Representative contour plots for assessing macrophage polarization by CD206-PE versus CD86-BV786 labeling. Red values indicate the M2/M1 ratio per plot, and contour lines separate 5% of the events, each with outliers as individual black dots. Ratios of CD206^+^ M2 versus CD86^+^ M1 macrophage counts were assessed at 3, 7, and 11 days post-injection between wild-type (green), mast cell-deficient (red), and mast cell-engrafted (blue) male (**b**) and female mice (**c**). *n* = 4–9 per group. Box plots display a median with a 25–75% percentile distribution. **d** Median M2/M1 values between male (circles with solid lines) and female (squares with dashed lines) over time with statistical analysis between sexes at the same timepoint. Quantified results were pooled from 2–3 independent experiments (*p < 0.05, **p < 0.01, ***p < 0.001, ****p < 0.0001).

From this ratio, we observed a lack of expected polarization shift toward being M2 dominant in male mast cell-deficient mice at day 11 post-injection compared to a significant shift in wild-type mice (Fig. 2a, b). Up to Day 11 was evaluated since beyond this timepoint, the ECM hydrogels were largely degraded and could not be consistently identified and isolated. To confirm that this difference was mast cell-specific, mast cell-engrafted mice were also similarly assessed. These mice were generated from the adoptive transfer of bone marrow-derived mast cells differentiated in vitro (Supplementary Fig. 2). These cells were injected subcutaneously throughout the dorsal region in deficient mice at least 5 weeks preceding biomaterial implantation to reconstitute the local resident mast cell population as previously described^38,60^. Mast cell engraftment was confirmed at this later age-matched timepoint of 11–13 weeks old by toluidine blue staining showing the presence of mast cells in dermal tissue and extracted ECM scaffold injections (Supplementary Fig. 6). These engrafted mice showed a restoration of the M2 shift similar to wild-type mice (Fig. 2a), which was also significant compared to the deficient group (Fig. 2b). Based on individual percentages of M2 and M1 macrophage populations, M2 macrophage percentages generally increased for all groups from day 3 to 11. For M1 macrophages, percentages were relatively higher for wild-type and engrafted mice at day 3 compared to deficient mice and decreased by day 11. In contrast, the percentage of M1 macrophages in deficient mice was relatively maintained around the same percentage from day 3 to 11 (Supplementary Fig. 7). This suggested the ratio results (Fig. 2b) occurred from a lack of transition of macrophages in deficient mice from the pro-inflammatory phenotype and not from a lack of M2 macrophage presence.

For female mice, a significant M2 dominant presence was determined for deficient mice compared to both wild-type and engrafted mice at the early day 3 timepoint (Fig. 2b). Assessing individual M2 versus M1 percentages, a higher relative percentage of M2 macrophages and a slight relative decrease in M1 macrophage percentages were observed indicating a more mixed contribution from these two macrophage subpopulations to the M2/M1 ratio (Supplementary Fig. 7d, f). Notably, a significantly higher percentage of total macrophages for female engrafted versus deficient mice were found at day 3, potentially from the relatively higher percentage of M1 macrophages or an unassessed macrophage subpopulation. A higher percentage of hybrid M21 macrophages at day 11 for female wild-type mice versus other groups was also determined, potentially suggesting greater stimulation of ECM remodeling responses^54^.

Comparing the differences in the polarization progression between sexes, we observed a skew toward more pro-inflammatory macrophage phenotypes for male mice (Fig. 2d). This was observed based on the delay in the M1 to M2 transition for male mice compared to female mice in the wild-type and engrafted groups though the overall transition was maintained by day 11 (Fig. 2d). Furthermore, in deficient mice, we observed a M1 dominant response throughout while female mice maintained an opposite M2 dominant response (Fig. 2d). Based on the individual percentages, a relatively higher percentage of M1 macrophages was found at early timepoints in wild-type and engrafted male mice, and throughout all timepoints for deficient mice compared to percentages for female mice (Supplementary Fig. 4).

Evaluation of other immune cell types using flow cytometry, including T cells (Supplementary Fig. 8), B-cells (Supplementary Fig. 9), dendritic cells (Supplementary Fig. 10), and mast cells (Supplementary Fig. 11) showed limited differences in deficient mice aside from the expected absence of mast cell presence compared to wild-type and engrafted models. Therefore, these results similarly supported that mast cells have limited impact on the infiltration of immune populations in response to ECM biomaterials, as also seen in Fig. 1, but do influence the phenotypes of immune cells, such as the relative polarization of macrophages.

**Fig. 3:**
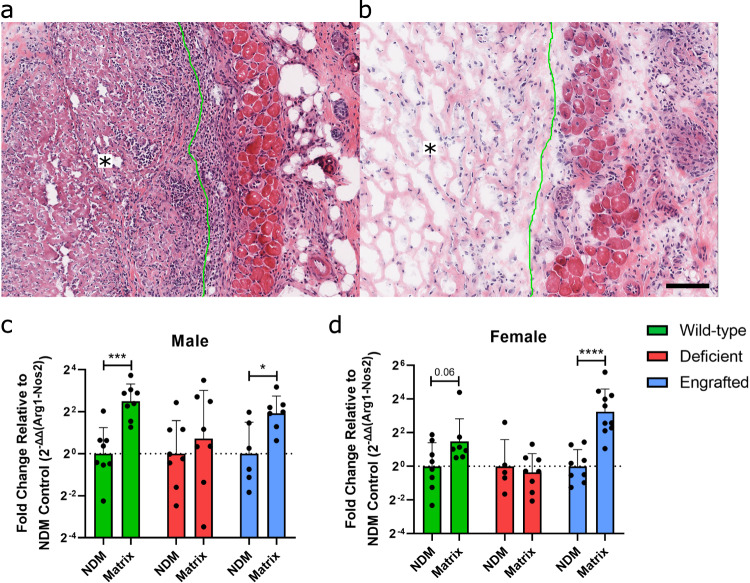
Comparison of mast cell influence in response to non-decellularized and decellularized ECM scaffolds. Representative hematoxylin and eosin staining in mast cell-deficient mice following dorsal subcutaneous injection of **a** non-decellularized and **b** decellularized ECM scaffold at 7 days post-injection (material outlined in green with an asterisk on side where material is present). Scale bar is 100 µm. Gene expression ratio at day 7 post-injection of M2/M1 macrophage markers Arg1 relative to Nos2 in **c** male and **d** female mice. Decellularized ECM scaffold in wild-type (green), mast cell-deficient (red), and mast cell-engrafted (blue) groups were compared by relative gene expression with 2^−^^ΔΔct^ normalized to non-decellularized (NDM) control per group. *n* = 6–10 per group. Data displayed as geometric mean ± geometric SD with results pooled from 2–3 independent experiments. (Value displayed for trend *p* ≤ 0.1, *p < 0.05, ***p < 0.001 and ****p < 0.0001).

Finally, to contextualize the degree of altered macrophage polarization observed in the mast cell-deficient mice, we compared the response of our decellularized ECM biomaterial in the mast cell-deficient model to a pro-inflammatory injectable control consisting of a similarly processed non-decellularized material (NDM) that still contained cellular debris. In a separate cohort of mice, H&E staining of injected NDM confirmed that immune rejection was elicited in mast cell-deficient mice (Fig. 3a), which was distinct from the decellularized ECM material response (Fig. 3b). Since the NDM did not gel and was not distinguishably maintained up to the day 11 timepoint, we used a different validated macrophage polarization assessment based on an M2/M1 gene expression ratio, Arg1/Nos2, where the M1-to-M2 transition to decellularized ECM was expected by day 7 post-injection relative to NDM as a pro-inflammatory point of reference^23,34,35^. At day 7 post-injection, a trending or significantly greater M2 dominant response for decellularized materials based on Arg1/Nos2 was determined for the wild-type and engrafted response in both sexes, matching previous results in wild-type mice^23^. In contrast, the deficient response was nonsignificant, with a similar average between the decellularized and non-decellularized materials in both sexes (Fig. 3c, d). This outcome aligned with the flow results described above for male mice, although, unlike the flow results, there was an M1 to M2 transition in the wild-type and engrafted female mice based on Arg1/Nos2 gene expression. As our flow results determined expression in individual cells compared to bulk gene expression, expression of Nos2 from other cells may have been responsible for this discrepancy. Differences may have also been magnified due to the reference point of a non-decellularized material being a highly inflammatory response. Overall, these results further support that mast cell deficiency leads to alteration in the expected M1-to-M2 transition that occurs for ECM biomaterials.

### Pro-inflammatory cytokine expression is similarly based on mast cell presence

**Fig. 4:**
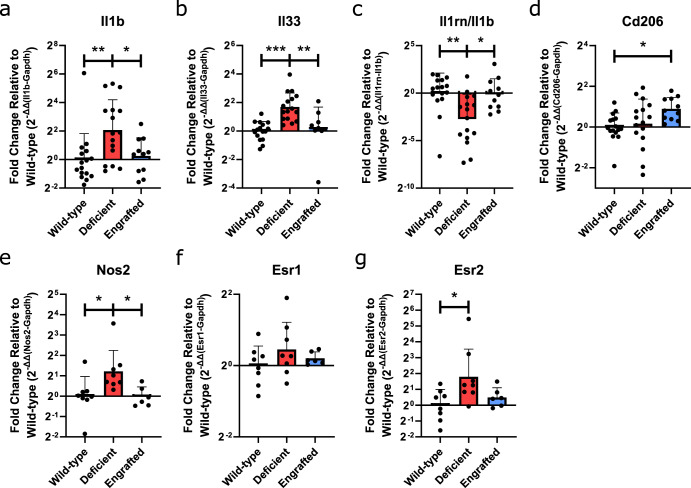
Immune marker profiling and hormone receptor gene expression demonstrating differentiation expression based on mast cell presence. qRT-PCR gene expression in deficient and engrafted normalized to wild-type expression by 2^−^^ΔΔct^ at day 11 in male mice for **a** Il1b and **b** Il33 relative to the housekeeping gene, Gapdh. **c** Gene expression ratio of receptor antagonist, Il1rn, relative to Il1b. **d** Gene expression of Cd206 relative to the housekeeping gene, Gapdh. qRT-PCR gene expression at day 3 in female mice measured for **e** Nos2, **f** Esr1, and **g** Esr2 relative to Gapdh. *n* = 11–16 per group for male samples, *n* = 6–8 per group for female samples. Data displayed as geometric mean ± geometric SD with results pooled from 2–3 independent experiments. (*p < 0.05, **p < 0.01, and ***p < 0.001).

For a better understanding of shifts in the overall immune microenvironment from mast cell absence, we evaluated the gene expression by qRT-PCR of several pro-inflammatory and pro-remodeling/anti-inflammatory markers associated with the mast cell secretome (Supplementary Table 1). RNA was collected from segments of the same subcutaneous injections that had been used for flow cytometry analysis. These segments were individually evaluated instead of being pooled per animal, as done in the flow cytometry analysis. At day 3 post-injection, only a significant increase in anti-inflammatory Il38 in male deficient versus wild-type mice was found (Supplementary Fig. 12a). At day 11 when significant shifts based on flow cytometry analysis were observed, significant differences in the IL-1 cytokine family, including increases in pro-inflammatory Il1b and Il33, and decreases in Il38, were found (Supplementary Fig. 12b). Further comparison with mast cell-engrafted male mice reversed these altered expression profiles for Il1b and Il33 (Fig. 4a, b) along with the ratio between IL-1 receptor antagonist, Il1rn, to Il1b (Fig. 4c). Expression of Cd206 was also significantly upregulated in engrafted mice compared to wild-type controls (Fig. 4d) matching the slightly increased predominance of M2 macrophages seen via flow cytometry (Fig. 2b).

Assessment of female mice at day 3 when dysregulation of macrophage polarization had been observed from flow analysis (Fig. 2b) determined only a significant upregulation of M1 marker Nos2 in deficient mice compared to wild-type that was also reversed in engrafted mice (Fig. 4e and Supplementary Fig. 13). Similar to our Arg1/Nos2 gene expression ratio results (Fig. 3), increased expression of Nos2, a M1 marker, in deficient mice conflicted with the results determined in our flow analysis. Given the consistency of Nos2 between these two assays, we investigated for a potential source of discrepancy between female mast cell deficiency and Nos2 expression. Secretion of the relevant protein, iNOS, can occur from a variety of different cell populations under certain conditions, including fibroblasts, keratinocytes, endothelial cells, vascular smooth muscle cells, and various immune cell populations^61^. Notably, stimulation of vascular smooth muscle cells and macrophages with female sex hormones has been shown to induce or enhance Nos2 expression^62,63^. Assaying for gene expression of estrogen receptors, Esr1 and Esr2 (Supplementary Table 2), determined a greater average expression for both receptors in the deficient mice compared to other groups that were significant for Esr2 between the deficient and wild-type mice (Fig. 4f, g). As mast cells have also been shown to be responsive to female sex hormones^64^, the absence of mast cell response or regulatory contribution might have exacerbated the response to these hormones in other cell populations.

Notably, when evaluating the variability in the response within groups, we determined a trending or significant positive correlation between the Δct values of Il1b and M2/M1 flow ratio (Supplementary Fig. 14). This result goes in line with studies supporting that shifts in both IL1B expression, a cytokine released by activated macrophages and a major mediator of the inflammatory response, and macrophage polarization are characteristic of tissue regeneration and repair^34,65^. The correlation analysis for wild-type mice excluded one Il1b Δct datapoint from Fig. 4a because it was an extreme outlier. This value was determined to be over 7 standard deviations away from the mean of the remaining data. Additionally, various outlier evaluations (Grubb’s, Dixon, Chi-squared test) were performed and confirmed this assessment. No other correlations were determined between datasets. Overall, these results demonstrate the influence of mast cells on the expression of the IL-1 cytokine family in the ECM biomaterial response in male mice. In contrast, the response in female mice appears mainly a result of shifts in macrophage response; however, potential influence from dysregulated female hormone responses with mast cell deficiency makes these results difficult to interpret based on standardized macrophage markers.

### Differential pro-inflammatory expression profile based on mast cell presence

As mast cells are known to secrete and influence a broad spectrum of immune responses, a more comprehensive multiplex analysis was carried out through a Nanostring panel. Only day 11 male mouse samples were assessed by a Nanostring immune panel as results consistently suggested a significant dysregulation in the immune response leading to a lack of transition to a pro-remodeling immune microenvironment from an ECM hydrogel. Furthermore, conflicting results observed from the female mast cell-deficient mice analysis, as described above, suggested that solely evaluating standardized immune markers with bulk gene expression in this group would not be sufficient.

**Fig. 5:**
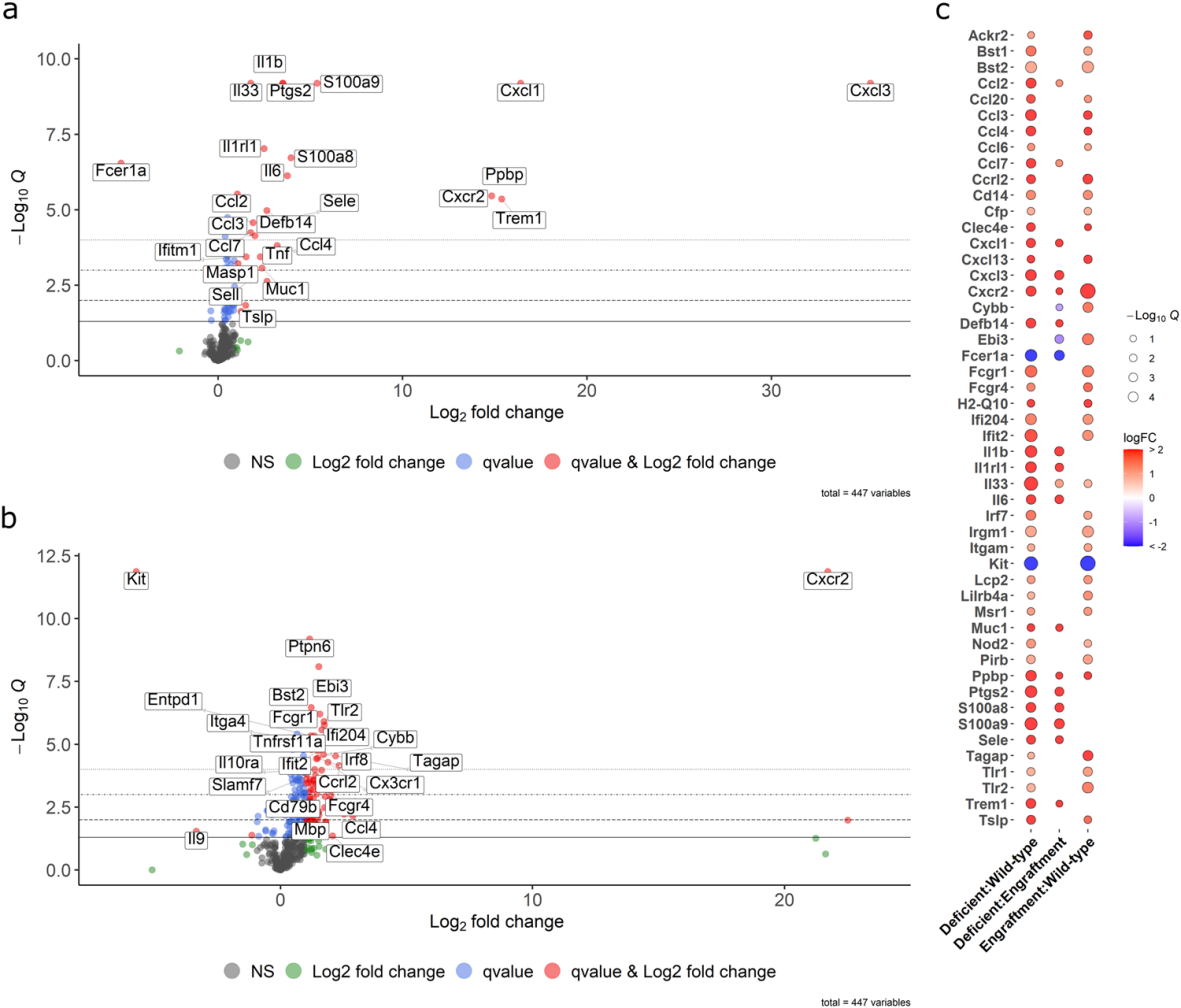
Mast cell-deficient male mice maintain late pro-inflammatory gene expression profile in response to ECM biomaterial implantation. Volcano plots labeled with the top 25 differentially expressed genes **a** in a multi-group comparison between mast cell-deficient versus both wild-type and mast cell-engrafted groups, and **b** in a pairwise comparison between engrafted versus wild-type samples. Dot colors indicate *q* < 0.05 (blue, red), absolute fold change greater than 2 (green, red) or nonsignificant (black). Dashed lines indicating different significance thresholds (*q < 0.05, **q < 0.01, ***q < 0.001 and ****q < 0.0001). **c** Bubble plot showing significant differentially expressed genes consistent across at least two pairwise analyses among the wild-type, deficient, and engrafted groups. The red to blue color scale displays log2 fold change that was up- or downregulated, respectively, and the circle size indicates the significance of the negative log_10_ transformed *q*-value (1: *q* = 0.1, 2: *q* = 0.01, 3: *q* = 0.001, 4: *q* = 0.0001). *n* = 8 per group pooled from 2–3 independent experiments.

PCA analysis of the top 50% of genes based on variance showed that the majority of wild-type and engrafted samples clustered in separate groups along PC1, with some samples dispersed between the two clusters. Mast cell-deficient samples were more distinct along PC2 and not as tightly clustered, potentially conveying increased variance due to the lack of regulatory influence from mast cells (Supplementary Fig. 15). Differentially expressed genes were assessed in multiple ways among the three groups (Supplementary Table 3). Generalized multi-group differential expression analysis of the deficient compared to the wild-type and engrafted responses determined differentially upregulated genes for a host of pro-inflammatory associated cytokines (Il1b, Il33, Il6, Tnf), chemokine and associated receptors (Ccl2, Ccl3, Ccl4, Ccl7, Cxcl1, Cxcl3, Cxcr2, Tslp), alarmins (S100a8, S100a9), enzymes (Ptgs2), and regulatory receptors (Clec4e, Il1rl1, Trem1). Additionally, upregulation of immune-associated adhesion proteins (Muc1, Sele, Sell), defensins (Defb14), complement pathway proteases (Masp1), and growth factors (Ppbp) were determined (Fig. 5a). Notably, mast cell marker, Fcer1a, was also significantly decreased for the mast cell-deficient group compared to wild-type and engrafted in both multi-group and pairwise comparisons, but not between the engrafted and wild-type samples (Fig. 5b, c and Supplementary Fig. 16), which further supported successful mast cell engraftment.

Pairwise analysis showed a similar pro-inflammatory response profile in deficient compared to wild-type alone (Ccl2, Ccl3, Ccl7, Cxcl1, Cxcl3, Il1b, Il1rl1, Il33, Il6, Ptgs2, S100a8, S100a9, Sele, Tnf, Trem1) (Supplementary Fig. 17a). Although the total number of differentially expressed genes between mast cell-deficient and engrafted samples was more limited, notably, these consisted of the various previously highlighted pro-inflammatory markers (Ccl2, Cxcl1, Cxcl3, Il1b, Il1rl1, Il33, Il6, Ptgs2, S100a8, S100a9, Sele, Trem1), conveying an inflammatory profile specifically related to the loss of the localized mast cell response (Supplementary Fig. 17b). This was absent in the pairwise comparison between engrafted to wild-type mice. Instead, differential expression highlighted the upregulation of cellular signaling components (Cd79b, Entpd1, Mbp, Ptpn6), membrane oxidases (Cybb), transcription factors (Pou2f2), immune cell and immunoglobulin-related regulatory receptors (Cd79b, Fcgr1, Fcgr4, Il10ra, Itga4, Slamf7, Tlr2, Tnfrsf11a), regulatory transcription factors (Irf8), immune cell activating proteins (Tagap), IFN-induced proteins (Ifi204, Ifit2), cytokine components (Ebi3), chemokines and associated receptors (Ccl4, Cx3cr1, Cxcr2), and downregulation of Il9 cytokine, which regulates cell growth and apoptosis (Fig. 5b). All together, these results convey an alternatively regulated immune profile in the engrafted versus wild-type response compared to the distinctly greater pro-inflammatory profile in the mast cell-deficient response.

Finally, specifically investigating significant differentially regulated genes consistent across at least two pairwise comparisons among the three sample groups confirmed matching upregulation of previously highlighed pro-inflammatory markers (Ccl2, Ccl7, Cxcl1, Cxcl3, Cxcr2, Il1b, Il1rl1, Il33, Il6, Ptgs2, S100a8, S100a9, Trem1), defensins (Defb14), and adhesion proteins (Muc1, Sele) in deficient versus either wild-type or engrafted responses. In contrast, the majority of these pro-inflammatory markers were not similarly upregulated between engrafted and wild-type samples. Instead, genes related to cell growth and signaling factors (Bst1, Bst2, Ppbp), immune regulatory receptors (Ackr2, Irf7, Lilrb4, Pirb, Tlr1, Tlr2), chemokines and associated regulatory receptors (Ccl2, Ccl3, Ccl4, Ccl6, Ccl20, Ccrl2, Cxcl13, Cxcr2, Tslp), MHC class I antigen presentation and signaling (H2-Q10, Pirb), immune cell activating proteins (Lcp2, Nod2, Tagap), integrins (Itgam), cytokine components (Ebi3), macrophage scavenger receptors (Msr1), IFN-induced proteins (Ifi204, Ifit2), complement pathway regulators (Cfp), regulatory GTPases (Irgm1), and myeloid marker (Cd14) were upregulated (Fig. 5c). Considering the different gene profile between the deficient and engrafted response where only mast cell presence differs, particularly for pro-inflammatory cytokines, these results support that local mast cell presence alone can significantly alter the immunomodulatory biomaterial response.

### Enrichment of pro-inflammatory pathways in the absence of mast cells

**Fig. 6:**
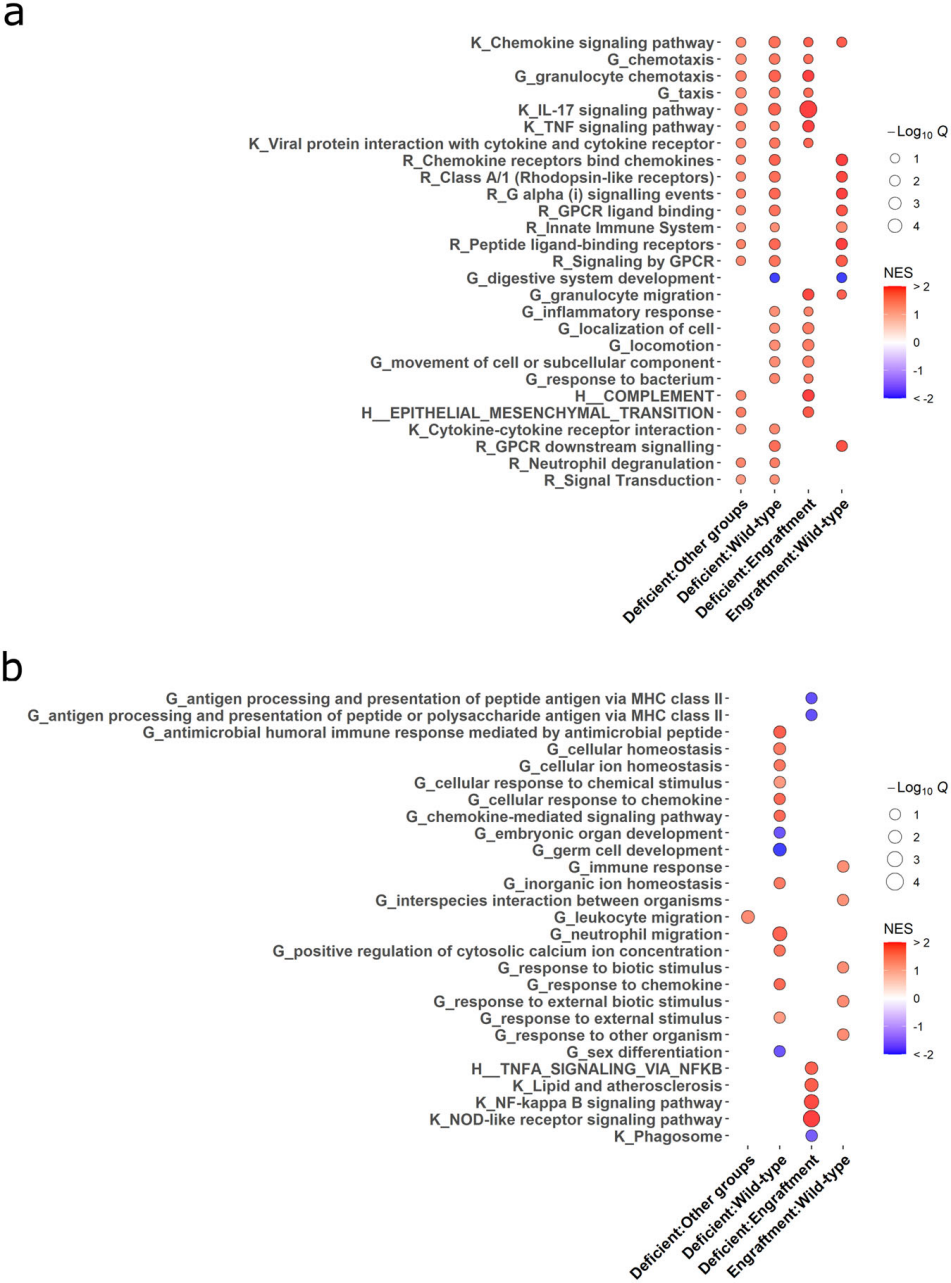
Mast cell-deficient male mice maintain late pro-inflammatory response to biomaterial implantation while mast cell engraftment restores physiological cellular responses to biomaterial stimuli. Bubble plot from gene set enrichment analysis for enriched pathways **a** consistent or **b** independently enriched among different group comparisons. Assessed databases included simplified biological process GO terms (“G_…”), KEGG pathways (“K_…”), Reactome pathways (“R_…”), and MSigDB hallmark gene sets (“H_…”). The red to blue color scale of each point represents the magnitude and directionality of up- and downregulated pathways based on normalized enrichment score (NES). Point size represents significance based on the negative of the log_10_ transformed *q*-value (1: *q* = 0.1, 2: *q* = 0.01, 3: *q* = 0.001, 4: *q* = 0.0001). *n* = 8 per group pooled from 2–3 independent experiments.

Gene set enrichment analysis was performed to evaluate trends that were consistent (Fig. 6a) or divergent (Fig. 6b) among different group comparisons for the deficient versus both wild-type and engrafted groups, deficient versus wild-type and engrafted groups individually, and engrafted versus wild-type groups (Supplementary Table 4). For comparisons versus the deficient response, enriched pathways corroborated previously made differential gene expression associations to pro-inflammatory processes. Specifically, deficient sample comparisons consistently enriched for pathways related to early or pro-inflammatory immune responses, including inflammatory responses, neutrophil degranulation, innate immune system, IL-17 and TNF signaling pathways, and viral protein-like responses (Fig. 6a). Similarly, several independent pairwise comparisons against the deficient samples showed upregulation of pro-inflammatory TNF, and NF-kappa B and NOD-like receptor signaling pathways (Fig. 6b).

In contrast, enriched gene sets for the engrafted versus wild-type responses showed a lack of significant upregulation along the majority of these pro-inflammatory pathways except for potential upregulation associated with the innate immune system and granulocyte migration (Fig. 6a). Instead, enrichment consistent with the other comparisons mainly consisted of signaling and receptor binding pathways such as related to generalized chemokine and G protein signaling related pathways (Fig. 6a). Independent enrichment pathways for engrafted versus wild-type comparison also showed upregulation of general immune and responses associated to biotic stimuli (Fig. 6b), suggesting a more normalized immune response profile similar to the wild-type group. Ultimately, these results further supported the significant contribution of mast cell presence to the pro-inflammatory transition of the ECM biomaterial-induced immune response.

## Discussion

Biomaterials have gained momentum as a pro-regenerative therapy through modulation of the innate and adaptive immune response. In particular, studies have focused on the influence of macrophages^21^ and T cells^24^ in inducing tissue remodeling progression. However, the immune response consists of a wide breadth of immune cell populations, and discoveries of significant contributions of understudied cell populations to immunotherapies have highlighted the need to expand our investigative focus^66^. In this study, we focused our investigation on the contribution of mast cells to the immunomodulatory response of decellularized ECM biomaterials. Within a short time following the subcutaneous deployment of various decellularized bioscaffolds, mast cells with no to minimal degranulation were identified in the nearby surrounding tissue, at the bioscaffold-tissue border, and in the core of the material, contrasting the degranulated states observed with synthetic biomaterial implants^3,29,67^. This corroborates a previous report where increased numbers of tryptase+ mast cells were found with an ECM hydrogel that improved cardiac function in a rat myocardial infarction model^17^, suggesting that greater mast cell presence can be supportive of therapeutic outcomes.

Implantation of the ECM biomaterial in male and female Kit^W-sh^ mast cell-deficient mice demonstrated similar or greater total and immune subpopulation-specific infiltration. This was observed despite the lack of mast cell-releasing factors such as histamine for vasodilation and chemokines known to contribute to driving the early immune response, particularly for neutrophils. Given that bioactive ECM components contain signals for promoting immune cell recruitment and migration^68^, naturally derived ECM biomaterials do not appear dependent on mast cells for early immune cell recruitment. Furthermore, although some evidence of neutrophilia has been previously observed in this mast cell-deficient model^69^, this did not manifest into significant differences in localized neutrophil infiltration in this experimental context.

Despite being typically xenogeneic, decellularized ECM biomaterials, including our ECM hydrogel^23^, are well known to promote a pro-remodeling response and M2 macrophage polarization given the majority of cellular antigens are stripped from the material^32,34,35^, which is even more thorough with the hydrogel form^23^. Furthermore, many xenogeneic ECM products are in clinical use. For our ECM hydrogel, this macrophage polarization response has been previously demonstrated in both wild-type and humanized mouse models^23^. Herein, two assays were utilized for evaluating the expected M2 versus M1 macrophage polarization: CD206^+^/CD86^+^ ratio by flow cytometry at various timepoints and Arg1/Nos2 gene expression relative to a pro-inflammatory non-decellularized control. Both methods demonstrated a significant difference in the ratio of alternatively activated M2 and classically activated M1 macrophages in the wild-type versus mast cell-deficient response. Furthermore, these differences were reversed by engraftment of mast cells in deficient mice, thereby supporting differences that were mast cell presence specific.

Interestingly, a discrepancy in the timing of differential macrophage polarization was seen between these two assessments. For male mice, both assays supported a consistent pro-inflammatory state for mast cell-deficient mice. In contrast, gene expression analysis suggested a faster M2 polarization in wild-type and mast cell-engrafted mice compared to flow analysis, which could have simply resulted from the different timing of macrophage marker expression. For female mast cell-deficient mice, this discrepancy was more distinct as the M1 dominant Arg1/Nos2 ratio and independently assessed increased Nos2 expression from bulk gene expression at early timepoints conflicted with the flow cytometry suggesting a consistent M2 macrophage state. These distinct and almost opposite outcomes observed for mast cell-deficient groups across sexes and assays were unexpected as this and previous research have demonstrated similar immunomodulatory responses across sexes in wild-type animal models^17,23,33,36^. However, as flow cytometry is measured at the individual cell level, these results were considered more representative of the actual macrophage state compared to bulk expression measurements.

To further evaluate the discrepancy in flow cytometry versus gene expression results in female mice, we investigated whether increased Nos2 expression could be specifically influenced in macrophages or other cell types based on sex. Though iNOS secretion is predominantly from macrophages, a large variety of immune and non-immune cells have been shown to secrete iNOS under certain conditions^61^. Sex differences have also been abundantly noted for the immune response, with female hormones and related receptors having been shown to elicit Nos2 expression in macrophages^63^ and other cell populations such as vascular smooth muscle cells^62,70^. This led to our additional investigation on female hormone receptor expression, showing increased expression of estrogen receptor, Esr2, in female deficient mice compared to wild-type. This relationship suggests this classical M1 marker’s bulk expression might not be representative of the macrophage response in female mast cell-deficient mice. Alternatively, since even in the in vitro setting, classical M2 versus M1 markers have shown inconsistency based on experimental parameters and stimuli^57^, the increased complexity of the in vivo setting likely exacerbates the potential for unique macrophage characteristics or subpopulations to arise. Furthermore, given the increase in receptor expression, influence from sex hormones might be more predominant with an absence of mast cells, but this would need to be confirmed in subsequent studies, likely with high-throughput single-cell methods. These results highlight the need for further investigation into the understudied field of sex/hormone-specific responses to biomaterial therapeutics.

For male mice samples, differences in pro-inflammatory gene expression profiles in the mast cell-deficient mice compared to both wild-type and mast cell-engrafted mice correlated with altered polarization determined from macrophage assessments. This was also highlighted in the enrichment analyses where mast cell-deficient samples were upregulated in early or pro-inflammatory-related gene sets. Notably, distinctly altered genes based on mast cell presence such as Il1b, and presence of immune cell populations such as M2 macrophages and degranulated mast cells have also been highlighted in comparisons between healing versus non-healing wounds in patients^71^. Ultimately, these comparisons support that mast cell presence aids in the temporal dynamics and regulation of the immunomodulatory response for an ECM biomaterial.

Considering the consistency of pro-inflammatory markers differing in the deficient male mouse response compared to either wild-type or engrafted responses, comparisons were also made between wild-type and engrafted groups to evaluate the degree mast cell engraftment restored the wild-type response. These results also provided evidence of whether the specific localization of mast cells around the biomaterial space was beneficial or detrimental. Based on differential analysis, significant genes were mainly associated with immune cell regulation, recruitment, differentiation, and activation along with general cell metabolic and signaling genes while lacking enrichment of distinct pro-inflammatory markers. Thus, while some differential genes and pathways were present between the wild-type and engrafted samples, the pro-inflammatory response was normalized to the wild-type response with mast cell engraftment. Notably, several differentially regulated genes that were not normalized with mast cell engraftment, such as Tslp and Il33, have been therapeutic targets in immunomodulatory biomaterial therapy studies for regulating chronic inflammatory responses^72–74^. Thus, further promoting localized non-degranulated mast cell presence could provide contributions extending beyond just mitigating initial pro-inflammatory responses by promoting beneficial regulatory responses in the biomaterial immune microenvironment.

One limitation of this study is that these results could not be extended into an injury model due to both technical and biological limitations of this approach for mast cell studies. As a technical limitation, the viscosity of the ECM hydrogel prevents intramyocardial injection into the thin heart wall in mouse myocardial infarction models leading to high mortality rates. This issue is not present in larger animals such as rats and pigs, but there is a lack of standardized transgenic models for these larger animals in general for studying a mast cell-specific role. Alternative methods to remove mast cell influence in these larger animal models, such as antibody-based conditional depletions, are limited by mast cell-specific antigen targets that would affect other hematopoietic populations. From a biological standpoint, mast cell deficiency inherently influences tissue remodeling and function with injury^75,76^, thus, making it difficult to definitively determine whether mast cell deficiency influences biomaterial-induced repair responses when inherent markers of chronic tissue diseases such as function or fibrosis are already significantly affected. With a more specific understanding of mast cell characteristics in vivo, genetic manipulations to specifically manipulate mast cell function, such as crossing *Mcpt5-Cre* transgenic mice with *IL-10*^fl/fl^ for mast cell-specific knockout of IL10 secretion^77^ and other similar strategies^78^. However, it is known that some off-targeting effects have been observed with these mouse models^78^, thus, this is still an actively developing field to improve mast cell-specific manipulations in animal models. Another limitation was the inability to evaluate the specific mast cell phenotype in vivo. Mast cells are a particularly difficult cell to isolate from tissue without loss of viability, eliciting degranulation, and altering their phenotype. To avoid these issues, mast cells are commonly only evaluated through immunohistochemistry methods since the tissue is preserved as quickly as possible. Advances in technology that have expanded on the analyses from these preserved tissue samples, such as by spatial transcriptomics, have the potential to better evaluate native mast cells in a high-throughput manner. Finally, our evaluations were done using a soft naturally derived hydrogel material or an injectable powder form of decellularized ECM, which allow for minimally invasive delivery and limited acute or prolonged tissue damage with implantation^79^. As mast cell recruitment and activation have been shown to be influenced by localized stiffness^80^, stiffness changes^81^, and mechanical forces^82^, it is possible that more rigid naturally derived materials may elicit a more classical response.

Overall, in contrast to previous in vivo studies of the mast cell response to synthetic biomaterials, which showed involvement in acute inflammation and fibrous capsule formation^3,29^, this study demonstrated that mast cells can contribute to the dynamic immunomodulatory response to pro-regenerative biomaterial scaffolds. This work highlights the need to delve deeper into the role of other immune cells beyond macrophages and T cells, as well as sex differences in pro-regenerative therapies, and supports the potential of mast cells as a critical immune regulatory element for stimulating endogenous tissue repair.

## Methods

All procedures in this study were approved by the Institutional Animal Care and Use Committee at the University of California San Diego and in accordance with the guidelines established by the Association for the Assessment and Accreditation of Laboratory Animal Care.

### ECM hydrogel and other injectable material preparation

An ECM hydrogel was generated from porcine left ventricular tissue based on previously established protocols^83^. In brief, porcine left ventricular tissue was isolated and minced into small pieces. The tissue was decellularized under mechanical agitation in a solution of phosphate-buffered saline (PBS) containing 1% (wt/vol) sodium dodecyl sulfate (SDS) (Fischer Scientific, Fair Lawn, NJ) with 0.5% 10,000 U/mL penicillin streptomycin (PS) (Gibco, Life Technologies, Grand Island, NY) until fully decellularized based on previously established criteria^83^. Once decellularized, the decellularized ECM was thoroughly rinsed to remove residual SDS, lyophilized, and milled into a fine powder. ECM powder was partially digested with 1 mg/mL pepsin in 0.1 M HCL solution for 48 h before the solution was neutralized to a pH of 7.4 and reconstituted to physiological salt concentrations for a 6 mg/mL ECM solution. Partially digested ECM solution was aliquoted, lyophilized and stored at −80 °C until resuspended with sterile water prior to injection, which self-assembled into hydrogels in vivo.

MicroMatrix®, a matrix powder derived from decellularized porcine urinary bladder tissue, was received from ACell Inc. and was prepared as a 6 mg/mL injectable suspension with sterile saline. Non-decellularized material was created as previously described^23^, where minced porcine left ventricular tissue was rinsed for one day in PBS with PS instead of multiple days of decellularization with SDS. The non-decellularized material was then similarly processed into an injectable form through lyophilization, milling, and partial pepsin digestion as the decellularized material described above. The non-decellularized material was then diluted to a 6 mg/mL injectable solution. Polyethylene glycol (PEG)-trilysine hydrogels were generated as previously described with slight modifications^84^. In brief, equal volumes of 100 mg/mL research grade 4 arm-Succinimidyl Glutarate-PEG (BroadPharm, San Diego, CA) and 1 mg/mL trilysine (Sigma-Aldrich, St. Louis, MO) in 1x PBS were mixed for hydrogel formation.

### Mast cell-deficient mouse model

Homozygous mast cell-deficient mouse, w-sh mice: B6.Cg-*Kit*^*W-sh*^/HNihrJaeBsmJ (Jackson Laboratory, Sacramento, CA) were bred in a UCSD vivarium for up to 11–13 weeks before being designated for subcutaneous procedures. Both male and female mice were utilized for investigation. Age-matched wild-type C57BL6/J mice were used as control animals.

### Bone marrow-derived differentiated mast cell culture

Bone marrow-derived cells were harvested from wild-type C57BL6/J mice that were euthanized by CO2 and cervical dislocation. Each femur was isolated and bone marrow was flushed with 10 mL of media, collected and pipetted into culture flasks incubated in a cell culture incubator at 37 °C and 5% CO_2_. Mast cell differentiation media consisting of DMEM media (Gibco) with 10% FBS (Gibco), 1% penicillin streptomycin (Gibco), sterile filtered conditioned culture medium from D11 hybridoma cells as a source of IL3, 100 uM MEM nonessential amino acids (Lonza), 1 mM sodium pyruvate (Gibco), 0.1 mM HEPES (Gibco), and 55 uM β-mercaptoethanol (Gibco). Non-adherent cells were passaged at days 3, 7, 10, 14, 18, 22 and 28 before differentiation of mast cells was assessed by flow cytometry with antibody markers for CD117 APC (2B8, Biolegend, San Diego, CA) and FcεRI PE (MAR-1, Biolegend). Cells at greater than ~95% purity were utilized up to 4–6 weeks from initial isolation before mast cell functionality was expected to subside.

For assessment of cell viability, 100,000 mast cells were plated into a 48-well plate (*n* = 4) with ECM hydrogel material at nongelling concentrations of 0.6 mg/mL or neutralized Collagen I, Rat Tail at concentrations of equivalent stiffness and collagen content of 0.25 mg/mL concentration^79^ (Corning®, Corning, NY) doped into culture media. Media-only culture was used as a control. alamarBlue^TM^ Cell Viability Reagent (Invitrogen, Waltham, MA) was added to each well at a tenth of the volume totaling 400 µL solution, and cells were incubated in a 37 °C, 5% CO_2_ cell culture incubator. At 0, 2, 4, and 8 h after plate set-up, fluorescent readings from alamarBlue reagent using 560 nm excitation and 590 emission were read on a Synergy^TM^ H4 multi-mode microplate reader using Gen5^TM^ software (Biotek®, Wilnooski, VT). The experimental set-up was repeated to demonstrate the relative reproducibility of the results.

### Mast cell-engrafted model generation

A mast cell-engrafted model was generated using a previously described protocol^38,60^. For injection procedures, animals were briefly put under anesthesia at 2% isoflurane on a nose cone, and mice were placed upright to expose the dorsal region. The dorsal region was shaved and cleaned with three intervals of betadine and 70% ethanol. Mast cell-engrafted mice were created by delivering eight to ten 100 µL subcutaneous injections of bone marrow differentiated mast cells in DMEM, totaling 4 million cells. Injections were at evenly spaced intervals throughout the dorsal region of 4- to 6-week-old deficient mice. Subsequent procedures were done in engrafted mice at 11–13 weeks old. Toluidine blue staining of subcutaneous tissue and injections confirmed the presence of mast cells in engrafted mice at the time of subcutaneous procedures and harvest (Supplementary Fig. 6). For comparisons with engrafted mice, age-matched mast cell-deficient mice injected with DMEM alone at similar timepoints for comparative studies, and wild-type mice were utilized as controls.

### Biomaterial injection and harvesting

For biomaterial injections, animals were briefly put under anesthesia using 2% isoflurane on a nose cone, and each mouse was injected with 6 mg/mL ECM hydrogel material, receiving two evenly spaced 200 μL subcutaneous injections in the upper and lower dorsal region. At one, three, seven, and eleven days post-injection, mice were euthanized by CO_2_ and cervical dislocation. Timepoints were selected based on expectations of the immune response being at the pro-inflammatory and pro-remodeling phases based on previous studies^23^, along with observation of consistent material retention and clear visibility up to the day eleven timepoint. General dorsal skin tissue was cut and flipped to expose the underside to observe the location and area of material injection. The injections, along with neighboring dermal tissue, were excised. Each harvested injection was divided into multiple parts for analysis by flow cytometry, staining or gene expression analysis as described below (*n* = 3–8 mice per group, 6–16 injections per group). For timepoints with notable differences between the deficient and wild-type responses, samples were collected across 2–3 experimental batches of animals to demonstrate consistency of results. Other materials, specifically 6 mg/mL non-decellularized digested tissue (high contaminant control), 6 mg/mL urinary bladder matrix powder suspended in sterile saline (ACell Inc, sterilized by electron beam irradiation and processed under cell culture conditions), and 100:1 mg/mL PEG-trilysine mixed solution prior to gelation (BroadPharm, Sigma-Aldrich), were delivered and isolated in a similar fashion.

### Flow cytometry

Pieces from multiple excised subcutaneous injections per animal were pooled and minced in ice-cold HBSS (Gibco) and enzymatically digested in a solution consisting of 1:1 solution of HBSS (calcium and magnesium supplemented) and 1% bovine serum albumin in PBS with 1 µM HEPES (Gibco), 300 U/mL collagenase type IV (Worthington Biochemical), 60 U/mL hyaluronidase (Sigma-Aldrich) and 10 U/mL DNase I (Sigma-Aldrich). The material in enzymatic digestion solution was incubated at 37 °C under mechanical agitation at 750 rpm on a thermomixer (Benchmark Scientific) for 45 min. Solutions were then kept in ice and FACs buffer consisting of 1% bovine serum albumin and 1 mM EDTA in DPBS lacking calcium and magnesium added to inhibit further enzyme reaction. Digested tissue was filtered through a 100 µm cell strainer. Cells were centrifuged at 400 rcf centrifugation at 4 °C and resuspended in HBSS. The cell suspension was stained with LIVE/DEAD^TM^ Fixable Aqua (ThermoFisher Scientific) for 10 min at 4 °C, and excess dye was quenched with FACs buffer. Cells were fixed and permeabilized by BD Cytofix/Cytoperm^TM^ Buffer (BD Biosciences) for 10 min and washed in BD Perm/Wash^TM^ Buffer (BD Biosciences). Cells were counted by hemocytometer and stained with antibody panels for immune cell subpopulations. General immune cell populations were stained with the following antibody panel: 1:400 CD11c BV421 (N418, #117329, Biolegend), 1:400 F4/80 BUV395 (T45–2342, #565614, BD Biosciences), 1:400 CD3 PerCp/Cy5.5 (17A2, #100218, Biolegend), 1:200 CD117 APC (2B8, #105811, Biolegend), 1:200 FcεRI PE (MAR-1, #134307, Biolegend), and 1:800 CD19 APC-Cy7 (6D5, #115530, Biolegend). An antibody panel for macrophage and T cell polarization was also stained for consisting of: 1:400 F4/80 BUV395 (T45–2342, #565614, BD Biosciences), 1:400 CD86 BV786 (GL-1, #105043, Biolegend), 1:200 CD206 PE (C068C2, #141705, Biolegend), 1:400 CD3 PerCp/Cy5.5 (17A2, #100218, Biolegend), 1:400 CD4 APC (GK1.5, #100408, Biolegend), 1:800 CD8a Alexa Fluor 488 (53–6.7, #100723, Biolegend), and 1:200 FoxP3 BV421 (MF-14, #126419, Biolegend). Antibodies and IgG isotype controls were stained in BD Perm/Wash^TM^ Buffer for 30 min at 4 °C before rinsing and resuspending in FACs buffer consisting of 1% bovine serum albumin and 1 mM EDTA in PBS for analysis. Stained cells were analyzed on a BD FACSCanto^TM^ II and BD LSRFortessa^TM^ X-20 (BD Biosciences). Gating and compensation were set based on positive, isotype and fluorescence minus one controls utilizing a mixed single cell suspension control sample derived from 7-day post-injection biomaterial, isolated spleen cells, and 4-week differentiated mast cells to ensure all cells of interest were present in distinguishable amounts. To account for batch-to-batch variability of harvested sample for analysis, isotype controls were rerun per set of tissue sample processed and stained with gating based on the outer perimeter of the isotype control contour plot with 10% population distribution between contour lines. Gating and flow data were processed in FlowJo v10.6.2 (FlowJo LLC, Ashland, OR).

### Quantitative real-time polymerase chain reaction (qRT-PCR)

Each flash-frozen or RNAlater^TM^ (Invitrogen) treated subcutaneous injection sample was homogenized with a mechanical rotator and then run through an RNeasy Mini kit (Qiagen, Germantown, MD) to extract RNA based on manufacturer instructions with an on-column DNase I digestion (Qiagen) for minimizing genomic DNA contamination. Superscript IV Reverse Transcriptase kit (Applied Biosystems, Foster City, MA) was used to synthesize cDNA with thermocycler settings of 65 °C for 5 min, 23 °C for 10 min, 55 °C for 10 min and 80 °C for 10 min. Eva Green Master Mix (Biotium, Fremont, CA) was used with custom-made forward and reverse primers (Supplementary Table 1, 2) at a final concentration of 0.2 µM for qPCR reactions. Samples were run in technical duplicate along with negative controls without template cDNA to confirm the lack of contamination from qPCR reagents. PCR reactions were run on a CFX95^TM^ Real-Time System (Bio-Rad, Hercules, CA) with the following thermal cycler settings: 30 s at 50 °C, 2 min at 95 °C, 40 cycles of 10 s at 95 °C, and 30 s at 55–65 °C based on pre-determined optimal primer efficiency amplification temperature. After completing 40 cycles of PCR amplification, automated melting curve analysis, consisting of increasing the thermal cycler temperature from 50 °C to 95 °C at 5 °C increments lasting 5 s each, for confirming singular amplicon product in each reaction vessel. Bio-Rad CFX Manager^TM^ 3.0 (Bio-Rad) was used for determining cycle threshold values from recorded signals based on a preset threshold.

### Nanostring multiplex gene expression analysis

For a comprehensive evaluation of the whole immune profile, equal amounts of RNA sample across subcutaneous injections per animal were pooled (n = 8 pooled subcutaneous injections representing mouse replicates per group) and were analyzed by Nanostring nCounter® MAX Analysis System with nCounter® Immunology Panel (Mouse) allowing for multiplexed assessment of 546 genes^85,86^. Samples were processed according to manufacturer instructions. In brief, RNA sample concentrations were measured on a Qubit 3.0 Fluorometer with a Qubit^TM^ RNA HS Assay kit. 70 µL of hybridization buffer was mixed with Immunology Panel Reporter CodeSet solution, and 8 µL of this master mix was mixed in a separate reaction vessel with 100 ng of RNA per tissue sample and RNA-free water up to 13 µL total. 2 µL of Capture ProbeSet was added to each vessel, mixed and placed on a thermocycler at 65 °C for 16–48 h before being maintained at 4 °C for less than 24 h. Nanostring nCounter Prep Station performed automated fluidic sample processing to purify and immobilize hybridized sample to cartridge surface. Digital barcode reads were analyzed by Nanostring nCounter® Digital Analyzer.

Results were analyzed by manufacturer nSolver^TM^ Analysis Software 4.0 and custom R scripts^87^ under R versions 4.04. Gene expression normalization and differential expression were determined by the NanostringDiff package^88^ with genes with average probe count one standard deviation below average negative control probe count excluded from analysis. Significance was set with a false discovery rate Benjamini-Hochberg method correction for calculating significance based on *q*-value <0.05 and an absolute log2 fold change ≥1. Heatmap of significant differentially expressed genes was displayed with the pheatmap package^89^. Volcano plots created with the EnhancedVolcano package^90^ display the top 25 genes based on aggregated ranking for lowest *q*-value and greatest absolute log2FC using the RankAggreg package^91^. Gene set enrichment analysis was performed with the clusterprofiler package^92^ across the following databases: Gene Ontology (GO) terms^93,94^ with redundancy reduced with the GOSemSim package^95^, Kyoto Encyclopedia Encyclopedia of Genes and Genomes (KEGG) pathways^96^, Reactome pathways^97^ with the ReactomePA package^98^, and hallmark gene sets from the Broad Institute and UCSD derived MSigDB^99^ with the msigdbr package^100^. KEGG pathways related to disease gene sets were excluded with the gageData package^101^, at least three genes were required to be enriched for gene sets, and significance cut-off for enrichment analysis was set at *q*-value <0.05 with a false discovery rate by Benjamini-Hochberg correction.

### Immunohistochemistry

Harvested samples were embedded into Tissue-Tek O.C.T. Compound (Sakura®, Torrance, CA) for cryosectioning. Cryosections from 2–3 different evenly spaced locations were used for all immunohistochemistry, and injections too small to obtain at least two locations (around less than 300 µm total width) were not processed. Transverse sections were taken to obtain a cross-section of the biomaterial injection and neighboring dermal tissue. Histological evaluation of total cell and mast cell infiltration was taken from sections stained with hematoxylin and eosin (H&E) or Toluidine Blue Stain, 1% w/v (Ricca Chemical, Arlington, TX) and scanned with a Leica Aperio ScanScope® CS2 (Leica, Buffalo Brove, IL) system. For fluorescent staining, slides were fixed with 4% paraformaldehyde in PBS (Thermo Scientific^TM^) and blocked with a buffered solution containing 3% bovine serum albumin (Gemini Bio-Products, Inc., West Sacramento, CA) and 0.1% Triton X-100 (Sigma) in PBS. The primary antibodies, 1:800 pan-macrophage marker anti-mouse F4/80 (BM8, #14–4801–82, eBioscience, San Diego, CA) and 1:200 anti-mouse Ly-6G/Ly-6C (RB6–8C5, #14–5931–82, eBioscience), were incubated for 12–18 h at 4 °C: Secondary antibody 1:500 anti-rat Alexa Fluor 488 (#A-21208, Thermo Scientific^TM^) with 1:10,000 Hoechst 33342 (Thermo Scientific^TM^) counterstain was incubated for 30–40 min at room temperature. Coverslips were mounted with Fluoromount^TM^ Aqueous Mounting Medium (Sigma) and allowed to dry, protected from light. Fluorescent images were scanned with the Leica Ariol® DM6000B system (Leica). Cellular density and co-staining quantification were done with custom MATLAB scripts^102^ (Mathworks, Natick, MA). Nuclei quantification was averaged across at least two locations per injection for nuclei staining (*n* = 8–10 injection replicates or 4–5 mouse replicates per group), while locations across both injections were averaged for co-staining analysis due to greater variability and background signal from material non-specific staining (*n* = 4–5 mice replicates per group).

### Statistical analysis

All data and plots are presented as mean ± SD unless noted otherwise. Statistics were performed in Prism 8 (GraphPad Software, San Diego, CA) or custom R scripts with publicly available packages. For choosing the appropriate statistical method, results were assessed for general normality through R based on visual inspection of histograms and QQ-plots and Shapiro-Wilk normality tests. Evaluation of similar larger experimental datasets utilizing the same methods was also evaluated to confirm nonsignificant normality test outcomes were not just due to instances of limited sample size. For image analysis and qRT-PCR results based on non-transformed Δct values, generally, no evidence of significant deviation from normality was observed. Additional evaluation from Dixon and Chi-squared outlier tests determined rare instances of significant Shapiro-Wilk results occurred from singular outliers. Thus, parametric statistical methods were applied to these types of datasets. For two group assessments, an unpaired student’s *t*-test was performed, while assessments across three or more groups were evaluated with a one-way ANOVA with a post-hoc Tukey with significance taken at *p* < 0.05.

For percentages and polarization ratios from flow data, Shapiro-Wilk results of *p* < 0.05 were commonly observed, and nonparametric statistical methods, specifically Kruskal-Wallis with Dunn’s multiple comparisons tests for comparisons greater than two groups, were applied.

For linear correlation assessments, Pearson correlation coefficient, r, was calculated through R packages with effect size calculated based on the covariance of the two variables being compared divided by the product of their standard deviations.

For analysis of differential expression of Nanostring data, a false discovery rate threshold of *q* < 0.05 based on the Benjamini-Hochberg method and an absolute log2 fold change threshold ≥1 were utilized. Gene set enrichment analysis also utilized a *q*-value <0.05 with a false discovery rate by Benjamini-Hochberg correction.

### Reporting summary

Further information on research design is available in the Nature Research Reporting Summary linked to this article.

### Supplementary information

Supplemental Info

Reporting Summary

## Data Availability

Raw Nanostring data files and normalized counts are available through GEO (GSE218383). Additional data may be requested from the authors.

## Code availability

Custom scripts utilized for image^102^ (10.5281/zenodo.7196556) and Nanostring^87^ (10.5281/zenodo.7190263) analyses utilized publicly available packages as described in the manuscript and have been uploaded to Zenodo repositories with example data and results.

## References

[1] Christy AL, Brown MA (2007). The multitasking mast cell: positive and negative roles in the progression of autoimmunity. J. Immunol..

[2] Amin K (2012). The role of mast cells in allergic inflammation. Respir. Med..

[3] Tang L, Jennings TA, Eaton JW (1998). Mast cells mediate acute inflammatory responses to implanted biomaterials. Proc. Natl Acad. Sci. USA.

[4] Thevenot PT, Baker DW, Weng H, Sun MW, Tang L (2011). The pivotal role of fibrocytes and mast cells in mediating fibrotic reactions to biomaterials. Biomaterials.

[5] Bhattacharya K (2007). Mast cell deficient W/Wv mice have lower serum IL-6 and less cardiac tissue necrosis than their normal littermates following myocardial ischemia-reperfusion. Int. J. Immunopathol. Pharm..

[6] Arac A (2014). Evidence that meningeal mast cells can worsen stroke pathology in mice. Am. J. Pathol..

[7] Jolly S, Detilleux J, Desmecht D (2004). Extensive mast cell degranulation in bovine respiratory syncytial virus-associated paroxystic respiratory distress syndrome. Vet. Immunol. Immunopathol..

[8] Janicki JS, Brower GL, Levick SP (2015). The emerging prominence of the cardiac mast cell as a potent mediator of adverse myocardial remodeling. Methods Mol. Biol..

[9] Wulff BC, Wilgus TA (2013). Mast cell activity in the healing wound: more than meets the eye?. Exp. Dermatol..

[10] Legere SA, Haidl ID, Légaré J-F, Marshall JS (2019). Mast cells in cardiac fibrosis: new insights suggest opportunities for intervention. Front. Immunol..

[11] Willenborg S (2014). Genetic ablation of mast cells redefines the role of mast cells in skin wound healing and bleomycin-induced fibrosis. J. Invest. Dermatol..

[12] Lennon EM, Borst LB, Edwards LL, Moeser AJ (2018). Mast cells exert anti-inflammatory effects in an IL10 model of spontaneous colitis. Mediators Inflamm..

[13] Shiota N (2010). Pathophysiological role of skin mast cells in wound healing after scald injury: study with mast cell-deficient W/W^V^ mice. Int. Arch. Allergy Immunol..

[14] Tellechea A (2016). Mast cells regulate wound healing in diabetes. Diabetes.

[15] Weller K, Foitzik K, Paus R, Syska W, Maurer M (2006). Mast cells are required for normal healing of skin wounds in mice. FASEB J..

[16] Christman KL (2019). Biomaterials for tissue repair. Science.

[17] Wassenaar JW (2016). Evidence for mechanisms underlying the functional benefits of a myocardial matrix hydrogel for post-MI treatment. J. Am. Coll. Cardiol..

[18] Badylak SF, Valentin JE, Ravindra AK, McCabe GP, Stewart-Akers AM (2008). Macrophage phenotype as a determinant of biologic scaffold remodeling. Tissue Eng. Part A.

[19] Sommerfeld SD (2019). Interleukin-36γ–producing macrophages drive IL-17–mediated fibrosis. Sci. Immunol..

[20] Nassiri S, Zakeri I, Weingarten MS, Spiller KL (2015). Relative expression of proinflammatory and antiinflammatory genes reveals differences between healing and nonhealing human chronic diabetic foot ulcers. J. Invest. Dermatol..

[21] Godwin JW, Pinto AR, Rosenthal NA (2013). Macrophages are required for adult salamander limb regeneration. Proc. Natl Acad. Sci. USA.

[22] Hasegawa T (2017). Transient inflammatory response mediated by interleukin-1β is required for proper regeneration in zebrafish fin fold. Elife.

[23] Wang RM (2017). Humanized mouse model for assessing the human immune response to xenogeneic and allogeneic decellularized biomaterials. Biomaterials.

[24] Sadtler K (2016). Developing a pro-regenerative biomaterial scaffold microenvironment requires T helper 2 cells. Science.

[25] Ozpinar EW, Frey AL, Cruse G, Freytes DO (2021). Mast cell-biomaterial interactions and tissue repair. Tissue Eng. Part B Rev..

[26] Ozpinar EW (2020). Dermal extracellular matrix-derived hydrogels as an in vitro substrate to study mast cell maturation. Tissue Eng. Part A.

[27] Methe KN (2020). Differential activation of immune cells for genetically different decellularized cardiac tissues. Tissue Eng. Part A.

[28] Ibrahim M (2017). Characterization of the foreign body response to common surgical biomaterials in a murine model. Eur. J. Plast. Surg..

[29] Avula MN, Rao AN, McGill LD, Grainger DW, Solzbacher F (2014). Foreign body response to subcutaneous biomaterial implants in a mast cell-deficient Kitw-Sh murine model. Acta Biomater..

[30] Orenstein SB, Saberski ER, Klueh U, Kreutzer DL, Novitsky YW (2010). Effects of mast cell modulation on early host response to implanted synthetic meshes. Hernia.

[31] Avula M (2016). Local release of masitinib alters in vivo implantable continuous glucose sensor performance. Biosens. Bioelectron..

[32] Brown BN (2012). Macrophage phenotype as a predictor of constructive remodeling following the implantation of biologically derived surgical mesh materials. Acta Biomater..

[33] Seif-Naraghi SB (2013). Safety and efficacy of an injectable extracellular matrix hydrogel for treating myocardial infarction. Sci. Transl. Med..

[34] Brown BN, Valentin JE, Stewart-Akers AM, McCabe GP, Badylak SF (2009). Macrophage phenotype and remodeling outcomes in response to biologic scaffolds with and without a cellular component. Biomaterials.

[35] Allman AJ (2001). Xenogeneic extracellular matrix grafts elicit a TH2-restricted immune response. Transplantation.

[36] Singelyn JM (2012). Catheter-deliverable hydrogel derived from decellularized ventricular extracellular matrix increases endogenous cardiomyocytes and preserves cardiac function post-myocardial infarction. J. Am. Coll. Cardiol..

[37] Traverse JH (2019). First-in-man study of a cardiac extracellular matrix hydrogel in early and late myocardial infarction patients. JACC Basic Transl. Sci..

[38] Grimbaldeston MA (2005). Mast cell-deficient W-sash c-kit mutant Kit W-sh/W-sh mice as a model for investigating mast cell biology in vivo. Am. J. Pathol..

[39] Cramer M (2022). Tissue response, macrophage phenotype, and intrinsic calcification induced by cardiovascular biomaterials: can clinical regenerative potential be predicted in a rat subcutaneous implant model?. J. Biomed. Mater. Res. A.

[40] Yu T (2016). Temporal and spatial distribution of macrophage phenotype markers in the foreign body response to glutaraldehyde-crosslinked gelatin hydrogels. J. Biomater. Sci. Polym. Ed..

[41] Mackey E (2016). Sexual dimorphism in the mast cell transcriptome and the pathophysiological responses to immunological and psychological stress. Biol. Sex Differ..

[42] Mackey E (2020). Perinatal androgens organize sex differences in mast cells and attenuate anaphylaxis severity into adulthood. Proc. Natl Acad. Sci. USA.

[43] Hamilton MJ, Hornick JL, Akin C, Castells MC, Greenberger NJ (2011). Mast cell activation syndrome: newly recognized disorder with systemic clinical manifestations. J. Allergy Clin. Immunol..

[44] Álvarez-Twose I (2010). Clinical, biological, and molecular characteristics of clonal mast cell disorders presenting with systemic mast cell activation symptoms. J. Allergy Clin. Immunol..

[45] Sadtler K (2019). Divergent immune responses to synthetic and biological scaffolds. Biomaterials.

[46] Sicari BM (2014). An acellular biologic scaffold promotes skeletal muscle formation in mice and humans with volumetric muscle loss. Sci. Transl. Med..

[47] Roch T (2011). Reducing the endotoxin burden of desaminotyrosine- and desaminotyrosyl tyrosine-functionalized gelatin. Macromol. Symposia.

[48] van Putten SM (2011). Endotoxin contamination delays the foreign body reaction. J. Biomed. Mater. Res. Part A.

[49] Poplutz M (2017). Endotoxin tolerance in mast cells, its consequences for IgE-mediated signalling, and the effects of BCL3 deficiency. Sci. Rep..

[50] De Filippo K (2013). Mast cell and macrophage chemokines CXCL1/CXCL2 control the early stage of neutrophil recruitment during tissue inflammation. Blood.

[51] Kolaczkowska E, Kubes P (2013). Neutrophil recruitment and function in health and inflammation. Nat. Rev. Immunol..

[52] Eissmann MF (2019). IL-33-mediated mast cell activation promotes gastric cancer through macrophage mobilization. Nat. Commun..

[53] Sadtler K (2016). The scaffold immune microenvironment: biomaterial-mediated immune polarization in traumatic and nontraumatic applications. Tissue Eng. Part A.

[54] Witherel CE (2021). Regulation of extracellular matrix assembly and structure by hybrid M1/M2 macrophages. Biomaterials.

[55] Muñoz-Rojas AR, Kelsey I, Pappalardo JL, Chen M, Miller-Jensen K (2021). Co-stimulation with opposing macrophage polarization cues leads to orthogonal secretion programs in individual cells. Nat. Commun..

[56] Huang J (2021). Single-cell RNA-seq reveals functionally distinct biomaterial degradation-related macrophage populations. Biomaterials.

[57] Murray PJ (2014). Macrophage activation and polarization: nomenclature and experimental guidelines. Immunity.

[58] Dong P (2016). CD86^+^/CD206^+^, diametrically polarized tumor-associated macrophages, predict hepatocellular carcinoma patient prognosis. Int. J. Mol. Sci..

[59] Spiller KL (2014). The role of macrophage phenotype in vascularization of tissue engineering scaffolds. Biomaterials.

[60] Ando T (2014). Critical role for mast cell stat5 activity in skin inflammation. Cell Rep..

[61] Xue Q, Yan Y, Zhang R, Xiong H (2018). Regulation of iNOS on immune cells and its role in diseases. Int. J. Mol. Sci..

[62] Sartoretto SM (2019). Involvement of inducible nitric oxide synthase and estrogen receptor ESR2 (ERβ) in the vascular dysfunction in female type 1 diabetic rats. Life Sci..

[63] You HJ, Kim JY, Jeong HG (2003). 17 beta-estradiol increases inducible nitric oxide synthase expression in macrophages. Biochem. Biophys. Res. Commun..

[64] Vliagoftis H (1992). Estradiol augments while tamoxifen inhibits rat mast cell secretion. Int. Arch. Allergy Immunol..

[65] Gan M-S, Yang B, Fang D-L, Wu B-L (2022). IL-1B can serve as a healing process and is a critical regulator of diabetic foot ulcer. Ann. Transl. Med..

[66] Helmink BA (2020). B cells and tertiary lymphoid structures promote immunotherapy response. Nature.

[67] Rezzani R (2004). Mast cells and the inflammatory response to different implanted biomaterials. Arch. Histol. Cytol..

[68] Chen P (2015). Collagen VI regulates peripheral nerve regeneration by modulating macrophage recruitment and polarization. Acta Neuropathol..

[69] Nigrovic PA (2008). Genetic inversion in mast cell-deficient (Wsh) mice interrupts corin and manifests as hematopoietic and cardiac aberrancy. Am. J. Pathol..

[70] Tsutsumi S (2008). Differential regulation of the inducible nitric oxide synthase gene by estrogen receptors 1 and 2. J. Endocrinol..

[71] Theocharidis G (2020). Integrated skin transcriptomics and serum multiplex assays reveal novel mechanisms of wound healing in diabetic foot ulcers. Diabetes.

[72] Sands RW (2020). Tuning cytokines enriches dendritic cells and regulatory T cells in the periodontium. J. Periodontol..

[73] Li T (2020). Graft IL-33 regulates infiltrating macrophages to protect against chronic rejection. J. Clin. Invest..

[74] Hussey GS (2019). Matrix bound nanovesicle-associated IL-33 activates a pro-remodeling macrophage phenotype via a non-canonical, ST2-independent pathway. J. Immunol. Regener. Med..

[75] Ngkelo A (2016). Mast cells regulate myofilament calcium sensitization and heart function after myocardial infarction. J. Exp. Med..

[76] Widiapradja A (2019). Regulation of cardiac mast cell maturation and function by the neurokinin-1 receptor in the fibrotic heart. Sci. Rep..

[77] Dudeck A (2011). Mast cells are key promoters of contact allergy that mediate the adjuvant effects of haptens. Immunity.

[78] Galli SJ (2015). Approaches for analyzing the roles of mast cells and their proteases in vivo. Adv. Immunol..

[79] Ungerleider JL, Johnson TD, Rao N, Christman KL (2015). Fabrication and characterization of injectable hydrogels derived from decellularized skeletal and cardiac muscle. Methods.

[80] Yu Y, Ren LJ, Liu XY, Gong XB, Yao W (2021). Effects of substrate stiffness on mast cell migration. Eur. J. Cell Biol..

[81] Yang HW (2018). An investigation of the distribution and location of mast cells affected by the stiffness of substrates as a mechanical niche. Int. J. Biol. Sci..

[82] Hu KK, Bruce MA, Butte MJ (2014). Spatiotemporally and mechanically controlled triggering of mast cells using atomic force microscopy. Immunol. Res..

[83] Singelyn JM (2009). Naturally derived myocardial matrix as an injectable scaffold for cardiac tissue engineering. Biomaterials.

[84] Rane AA (2011). Increased infarct wall thickness by a bio-inert material is insufficient to prevent negative left ventricular remodeling after myocardial infarction. PLoS ONE.

[85] Jardine L (2019). Lipopolysaccharide inhalation recruits monocytes and dendritic cell subsets to the alveolar airspace. Nat. Commun..

[86] Hussain S (2020). TLR5 participates in the TLR4 receptor complex and promotes MyD88-dependent signaling in environmental lung injury. Elife.

[87] Wang, R. M. NanoString differential expression analysis with NanoStringDiff package. Zenodo. 10.5281/zenodo.7190263 (2022).

[88] Wang H (2016). NanoStringDiff: a novel statistical method for differential expression analysis based on NanoString nCounter data. Nucleic Acids Res..

[89] Kolde, R. pheatmap: pretty heatmaps. https://github.com/raivokolde/pheatmap.git (2015).

[90] Blighe, K., Rana, S. & Lewis, M. EnhancedVolcano: publication-ready volcano plots with enhanced colouring and labeling. R package version 1.6.0. 10.18129/B9.bioc.EnhancedVolcano (2020).

[91] Pihur V, Datta S, Datta S (2009). RankAggreg, an R package for weighted rank aggregation. BMC Bioinformatics.

[92] Yu G, Wang L-G, Han Y, He Q-Y (2012). clusterProfiler: an R package for comparing biological themes among gene clusters. OMICS.

[93] Ashburner M (2000). Gene ontology: tool for the unification of biology. The Gene Ontology Consortium. Nat. Genet..

[94] The Gene Ontology Consortium. (2021). The Gene Ontology resource: enriching a GOld mine. Nucleic Acids Res..

[95] Yu G (2010). GOSemSim: an R package for measuring semantic similarity among GO terms and gene products. Bioinformatics.

[96] Kanehisa M, Goto S (2000). KEGG: kyoto encyclopedia of genes and genomes. Nucleic Acids Res..

[97] Fabregat A (2016). The Reactome pathway Knowledgebase. Nucleic Acids Res..

[98] Yu G, He Q-Y (2016). ReactomePA: an R/Bioconductor package for reactome pathway analysis and visualization. Mol. Biosyst..

[99] Subramanian A (2005). Gene set enrichment analysis: a knowledge-based approach for interpreting genome-wide expression profiles. Proc. Natl Acad. Sci. USA.

[100] Dolgalev, I. msigdbr: MSigDB gene sets for multiple organisms in a tidy data format. R package version 7.2.1. https://igordot.github.io/msigdbr/ (2020).

[101] Luo, W. gageData: auxillary data for gage package. R package version 2.28.0. 10.18129/B9.bioc.gageData (2020).

[102] Wang, R. M. MATLAB script for analyzing images from Leica scanning microscope with .scn extension. Zenodo. 10.5281/zenodo.7196556 (2022).

