# Supplementary material for: Immunomodulatory contribution of mast cells to the regenerative biomaterial microenvironment
Source: NPJ Regen Med. 2023 Sep 20;8:53. doi: 10.1038/s41536-023-00324-0 (PMC10511634; doi:10.1038/s41536-023-00324-0)
Supplement: Supplementary file 1 — Supplemental Info [file 41536_2023_324_MOESM1_ESM.pdf]

Supplementary Information

Supplementary Figures

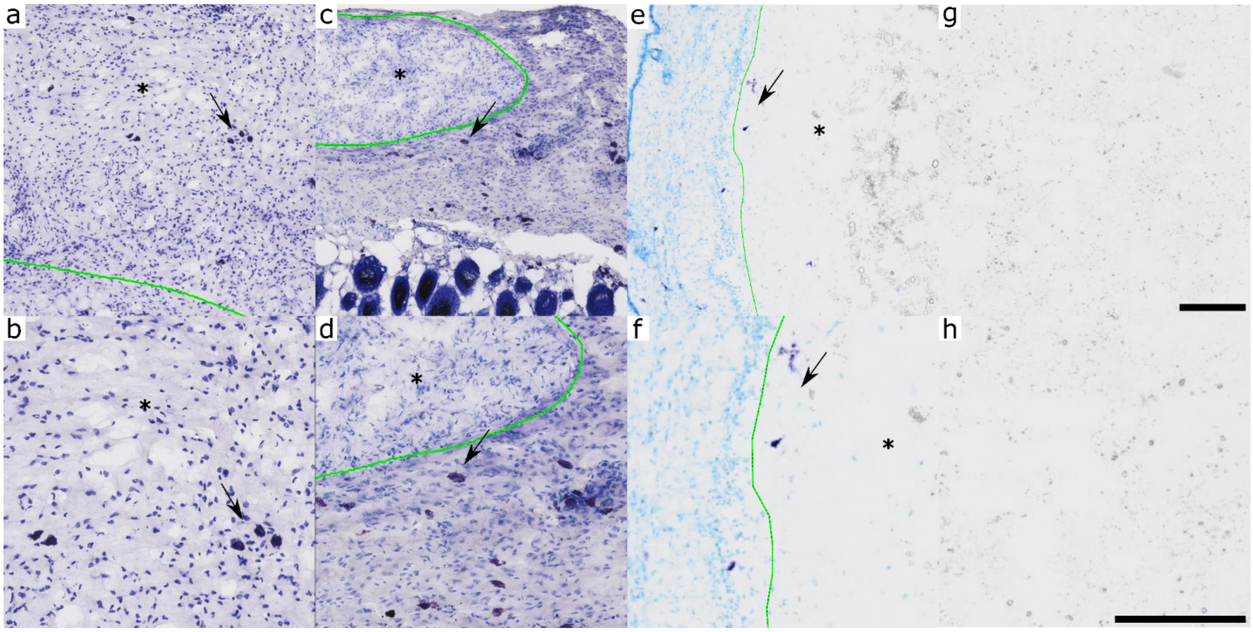

Supplementary Figure 1: Toluidine blue staining of subcutaneously injected ECM hydrogel, urinary bladder matrix powder, and synthetic PEG-trilysine hydrogel. Representative and zoomed toluidine blue staining images of a subcutaneously injected **(a, b)** ECM hydrogel scaffold, **(c, d)** milled urinary bladder matrix, and **(e, f)** border and **(g, h)** interior of a PEG-trilysine hydrogel (material outlined in green with asterisk on side where material is present) and neighboring dermal tissue in male wild-type mice 3 days post-injection. Region with mast cells staining is indicated by a black arrow. Scale bar is 200 μm.

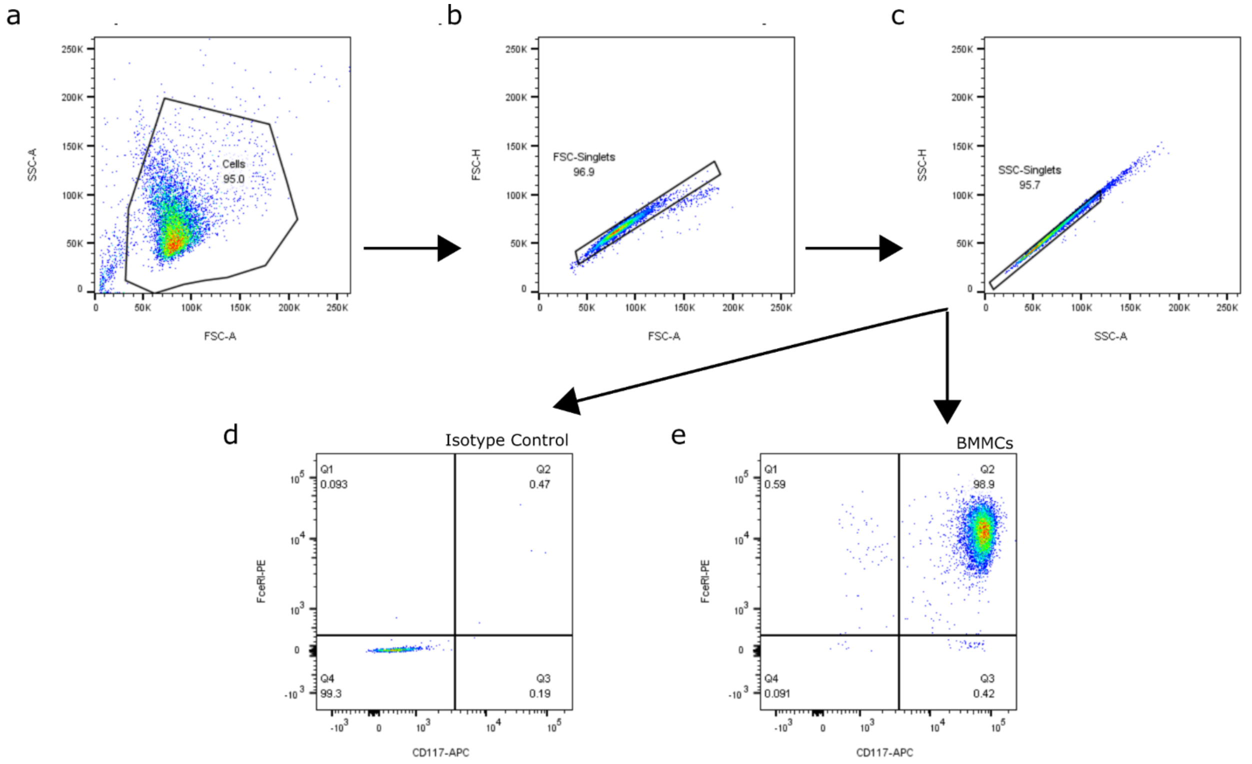

Supplementary Figure 2: Gating scheme for bone marrow derived differentiated mast cell culture purity. Representative gating of mast cells for assessing purity at four to six weeks of differentiation protocol. (**a)** Cells and **(b-c)** double discrimination of singlets based on forward and side scatter. Quadrant gating cut-offs were set relative to **(d)** isotype control for **(e)** CD117^{+}FcƐRI^{+} mast cells.

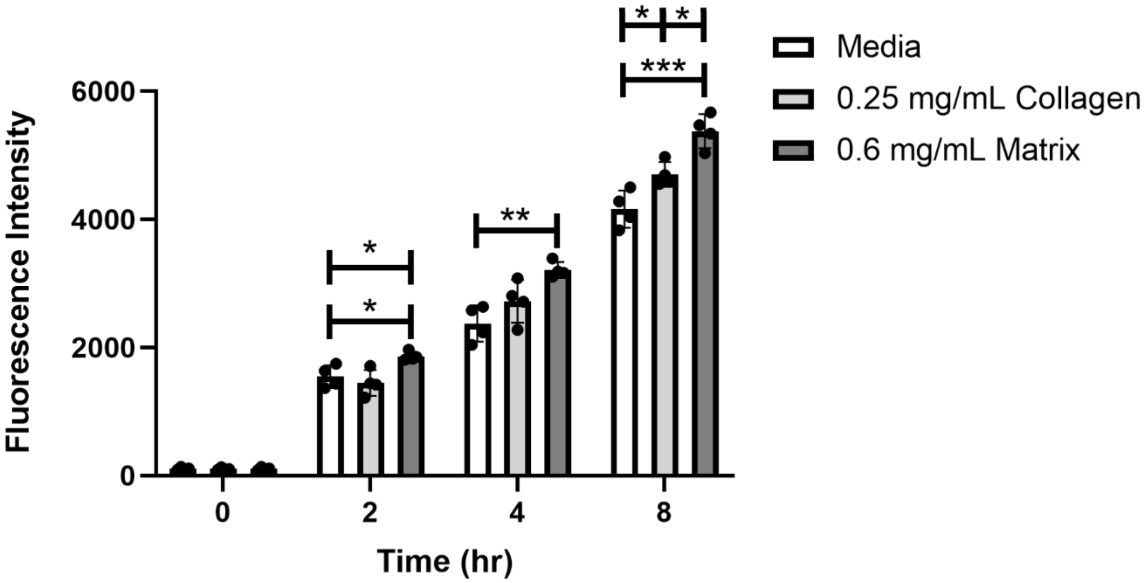

Supplementary Figure 3: Viability assay of mast cells incubated with ECM hydrogel. Four week differentiated bone marrow derived mast cells were incubated with ECM hydrogel material, collagen or culture media control. Collagen concentration was chosen based on previous studies showing analogous material properties to ECM hydrogels at this relative concentration[1]. AlamarBlue™ fluorescent readings were taken at 0, 2, 4, and 8 hours following plate set-up. n = 4 per group. Data displayed as mean ±SD. (*$p < 0.05$, **$p < 0.01$, ***$p < 0.001$).

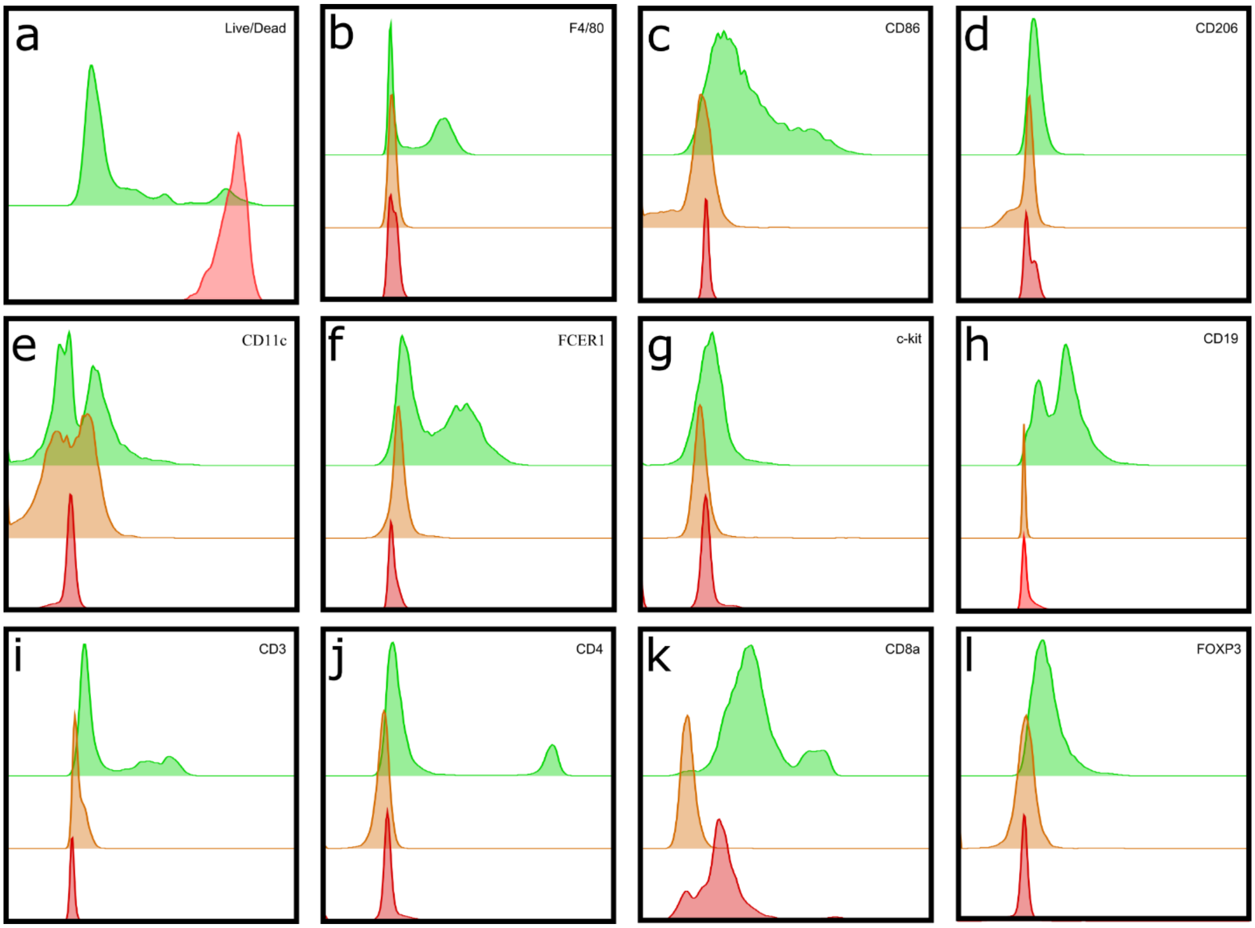

Supplementary Figure 4: Gating controls for flow cytometry markers. (a) LIVE, DEAD^{TM} Aqua thresholds utilized ethanol fixed sample as control (red) for determining live cell gating (green). **(b-l)** Gating for fluorescent antibody markers was set for positive signal (green) based on isotype (red) and fluorescence minus one controls (orange).

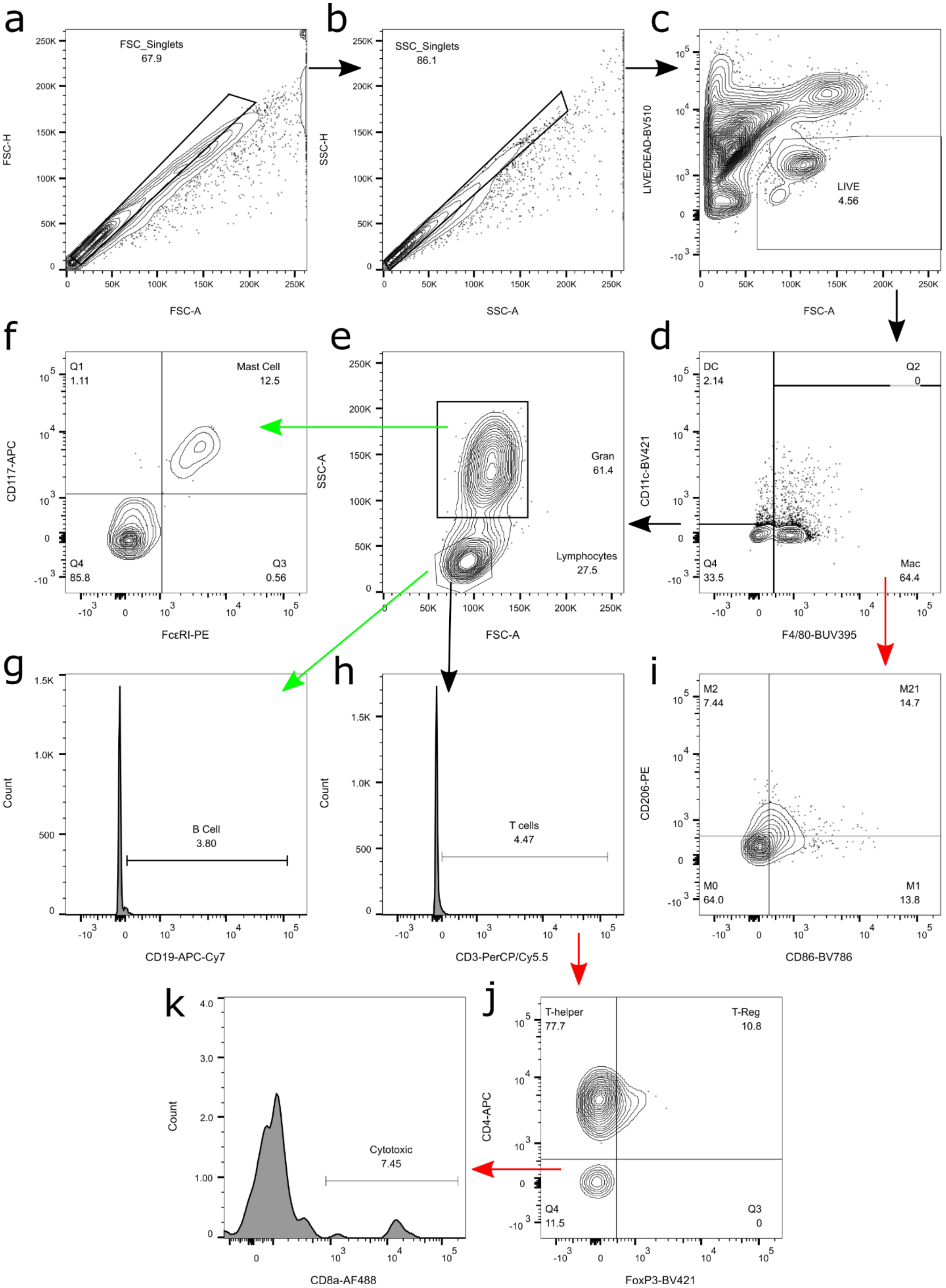
a
FSC_Singlets
67.9
FSC-H
FSC-A
b
SSC_Singlets
86.1
SSC-H
SSC-A
c
LIVE/DEAD-BV510
LIVE
4.56
FSC-A
d
DC
2.14
Q2
0
Q4
33.5
Mac
64.4
CD11c-BV421
F4/80-BUV395
e
Gran
61.4
Lymphocytes
27.5
SSC-A
FSC-A
f
Q1
1.11
Mast Cell
12.5
Q4
85.8
Q3
0.56
CD117-APC
FcεRI-PE
g
B Cell
3.80
Count
CD19-APC-Cy7
h
T cells
4.47
Count
CD3-PerCP/Cy5.5
i
M2
7.44
M21
14.7
M0
64.0
M1
13.8
CD206-PE
CD86-BV786
j
T-helper
77.7
T-Reg
10.8
Q4
11.5
Q3
0
CD4-APC
FoxP3-BV421
k
Cytotoxic
7.45
Count
CD8a-AF488

Supplementary Figure 5: Gating Scheme for flow cytometry analysis. Representative gating of single cell suspension isolated from subcutaneously injected ECM hydrogel. General gating for both panels (black arrows) started with double discrimination of singlets based on **(a)** forward and **(b)** side scatter. (**c)** Live cells were gated based lack of stain uptake and forward scatter to distinguish debris. **(d)** Macrophage and dendritic cell populations were gated based on F4/80^{+} and CD11c^{+}, respectively. **(e)** F4/80^{-} populations were separated into granulocyte and lymphocytes. For a general immune cell population panel (green arrow), granulocytes were assessed as **(f)** CD117^{+}FcƐRI^{+} mast cells and lymphocytes for **(g)** CD19^{+} B cells and **(h)** CD3^{+}CD19^{-} for T cells. For immune cell polarization panel (red arrow), **(i)** F4/80^{+} macrophage polarization was assessed for numbers of M1 CD86^{+}CD206^{-} and M2 CD86^{-}CD206^{+}. CD3+ T cells were assessed as **(j)** CD4^{+}FOXP3^{-} T helper cells, CD4^{+}FOXP3^{+} regulatory T cells, and **(k)** CD4^{-}FOXP3^{-}CD8a^{+} cytotoxic T cells.

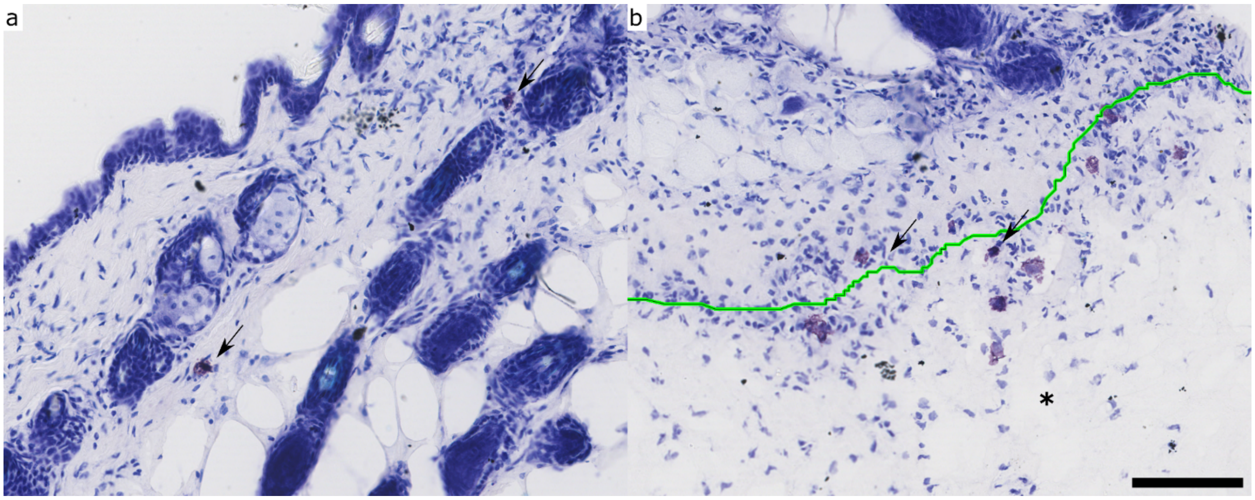

Supplementary Figure 6: Mast cells engraftment in deficient mice. Representative toluidine blue staining in 11-13 week old deficient mice following dorsal subcutaneous injection of *in vitro* differentiated bone marrow derived mast cells at 4-6 weeks old showing reconstitution of mast cells in the **(a)** skin and infiltrating into subcutaneously injected **(b)** ECM scaffold at 3 days post-injection (material outlined in green with asterisk on side where material is present). Mast cells staining is indicated by a black arrow with both resting and degranulated mast cells observed at this timepoint. Scale bar is 100 μm.

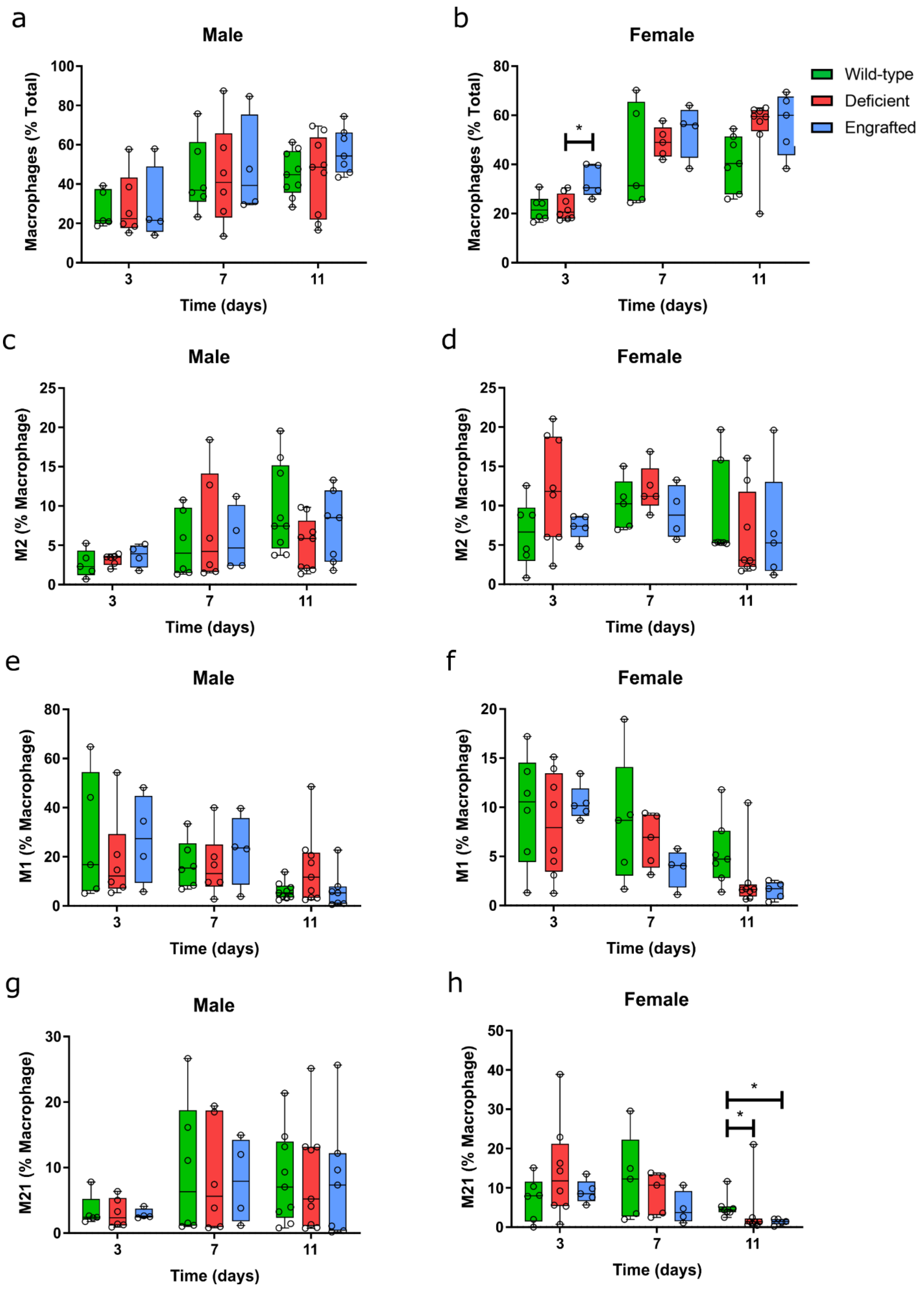
a
Male
Macrophages (% Total)
Time (days)
b
Female
*
Wild-type
Deficient
Engrafted
c
Male
M2 (% Macrophage)
d
Female
e
Male
M1 (% Macrophage)
f
Female
g
Male
M21 (% Macrophage)
h
Female

Supplementary Figure 7: Flow cytometry of total and macrophage polarized subpopulations. Percentages of total F4, 80^+ macrophages relative to live cells **(a, b)**, and $CD206^+$ M2 **(c, d)**, $CD86^+$ M1 **(e, f)** and co-expressing $CD206^+CD86^+$ M21 **(g, h)** macrophages relative to total macrophages at three, seven, and eleven days post-injection between wild-type (green), deficient (red), and engrafted (blue) male **(a, c, e, g)** and female mice **(b, d, f, h)** with median values indicated by a black bar per group. n = 4-9 per group. Box plots display median with 25-75% percentile distribution.

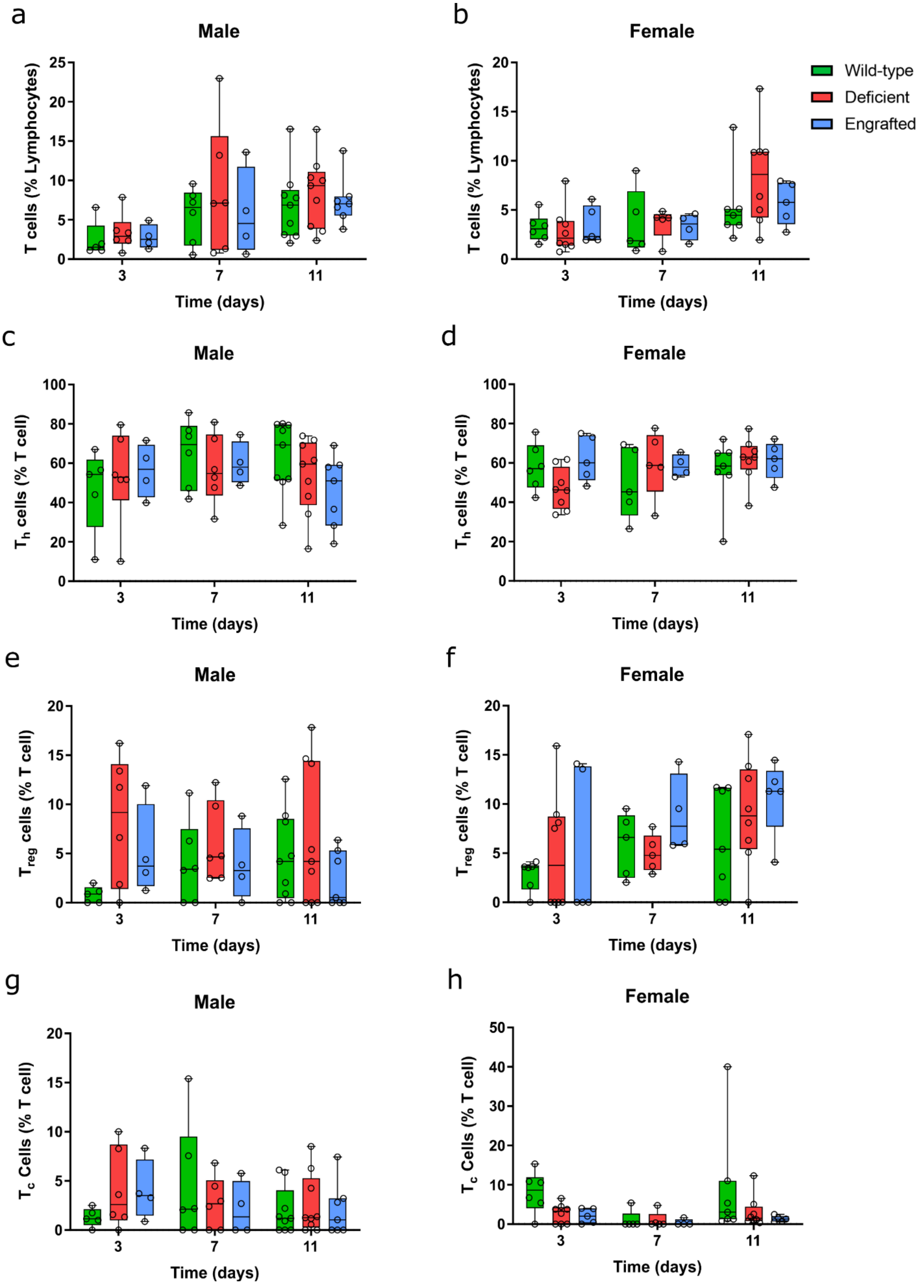
a
Male
T cells (% Lymphocytes)
Time (days)
b
Female
Wild-type
Deficient
Engrafted
c
Male
T_h cells (% T cell)
d
Female
e
Male
T_{reg} cells (% T cell)
f
Female
g
Male
T_c Cells (% T cell)
h
Female

Supplementary Figure 8: Flow cytometry of total T cells and differentiated subpopulations. Percentages of total $CD3^+$ T cells relative to total live cells **(a, b)**, and $CD4^+$ T-helper (T_h) cells **(c, d)**, $CD4^+FOXP3^+$ regulatory T (T_{reg}) cells **(e, f)** and $CD8^+$ cytotoxic T (T_c) cells **(g, h)** relative to total T cells at three, seven, and eleven days post-injection between wild-type (green), deficient (red), and engrafted (blue) male **(a, c, e, g)** and female mice **(b, d, f, h)** with median values indicated by a black bar per group. n = 4-9 per group. Box plots display median with 25-75% percentile distribution.

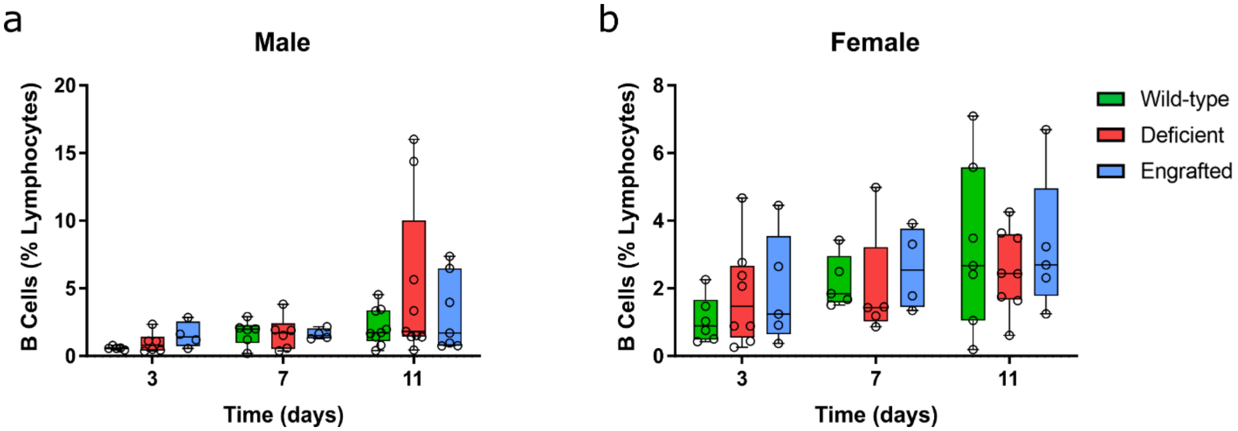

Supplementary Figure 9: Flow cytometry of B-cells. Percentages of CD19⁺ B cells relative to total live cells at three, seven, and eleven days post-injection between wild-type (green), deficient (red), and engrafted (blue) male **(a)** and female mice **(b)** with median values indicated by a black bar per group. n = 4-9 per group. Box plots display median with 25-75% percentile distribution.

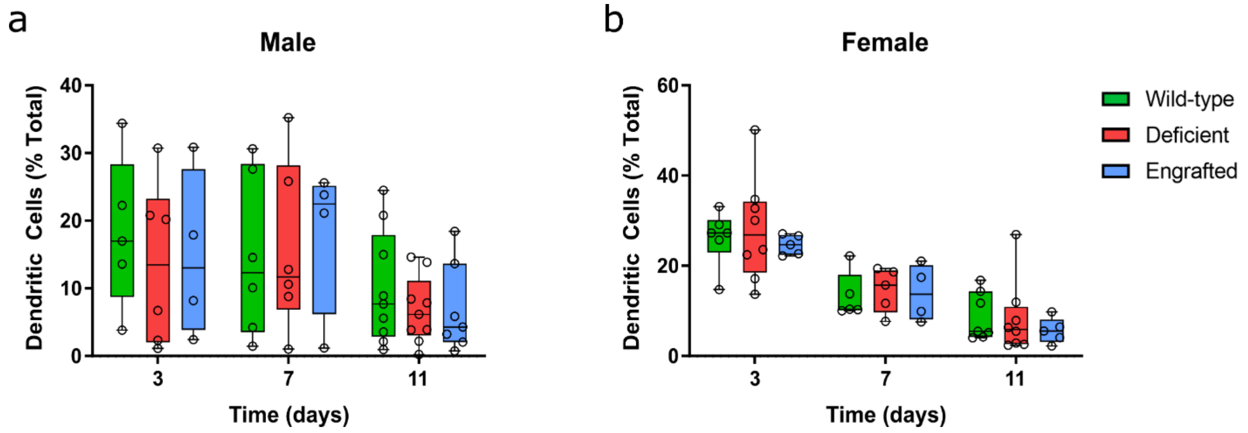

Supplementary Figure 10: Flow cytometry of dendritic cells. Percentages of CD11c^{+}F4, 80^{-} dendritic cells relative to total live cells at three, seven, and eleven days post-injection between wild-type (green), deficient (red), and engrafted (blue) male **(a)** and female mice **(b)** with median values indicated by a black bar per group. n = 4-9 per group. Box plots display median with 25-75% percentile distribution.

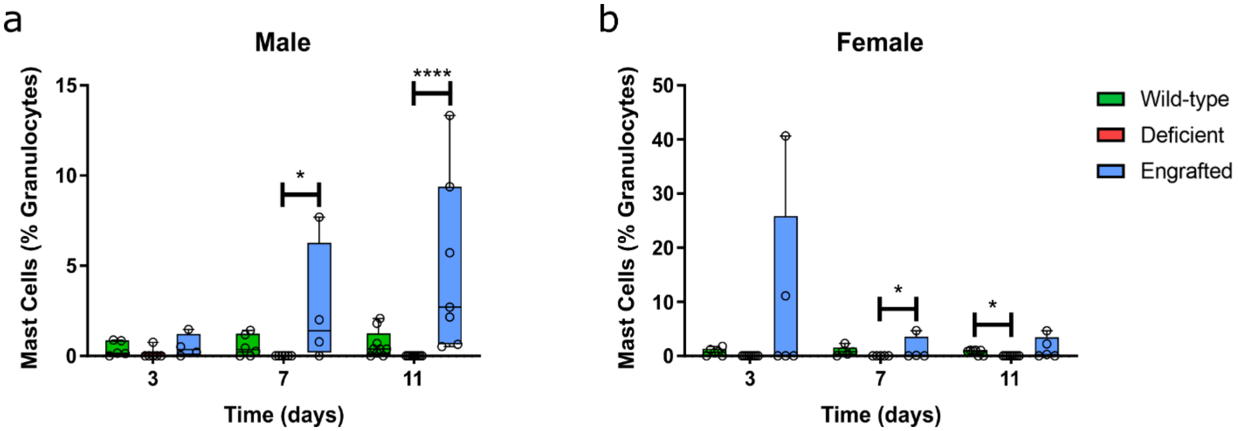

Supplementary Figure 11: Flow cytometry of mast cells. Percentages of CD117$^+$FcƐRI$^+$ mast cells relative to total live cells at three, seven, and eleven days post-injection between wild-type (green), deficient (red, not present), and engrafted (blue) male **(a)** and female mice **(b)**. n = 4-9 per group. Box plots display median with 25-75% percentile distribution.

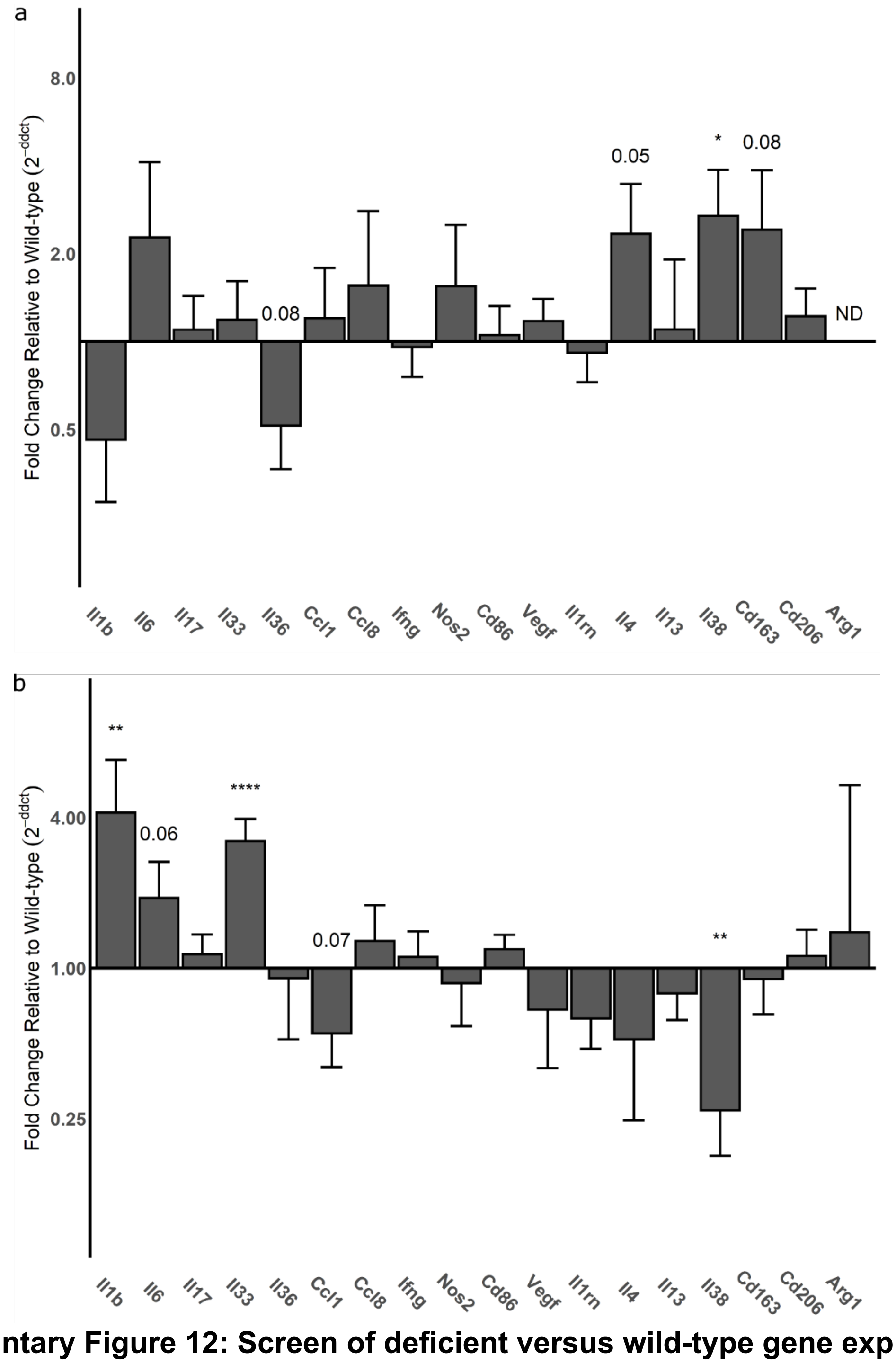

Supplementary Figure 12: Screen of deficient versus wild-type gene expression in male mice. qPCR results of pro-inflammatory versus pro-remodeling immune markers at day 3 **(a)** and day 11 **(b)** in male mice. Fold change and significance of deficient mouse sample gene expression was normalized relative to wild-type gene expression. Gene expression plotted as geometric mean ± geometric SEM. (ND = not detected, value displayed for trend $p \leq 0.1$, *$p < 0.05$, **$p < 0.01$, and ****$p < 0.0001$). n = 11-16 per group.

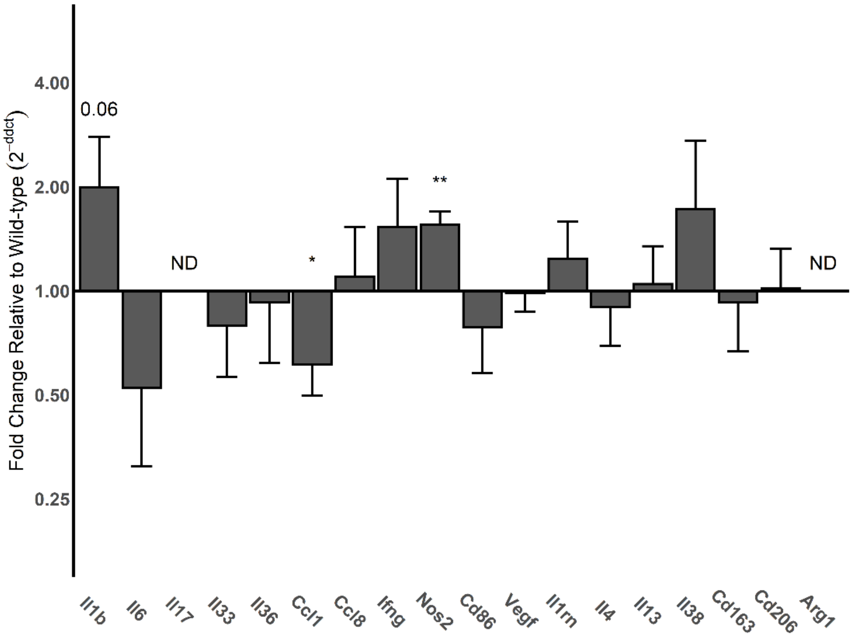

Supplementary Figure 13: Screen of deficient versus wild-type gene expression in female mice. qPCR results of pro-inflammatory versus pro-remodeling immune markers at day 3 in female mice. Fold change and significance of deficient mouse sample gene expression was normalized relative to wild-type gene expression. Gene expression plotted as geometric mean ± geometric SEM. (ND = not detected, value displayed for trend $p \leq 0.1$, *$p < 0.05$, **$p < 0.01$). n = 6-8 per group.

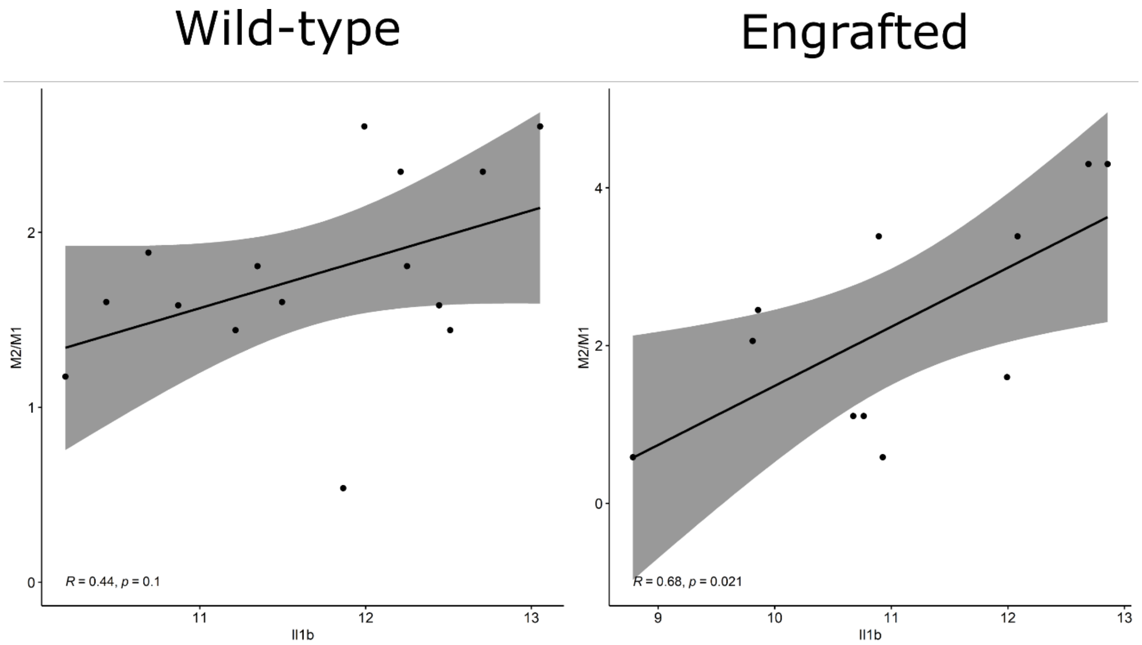

Supplementary Figure 14: Linear regression plot of macrophage polarization ratio and Il1b gene expression. Pearson correlation plots displaying association between M2/M1 ratio from CD206/CD86 macrophage flow ratio values and Il1b Δct gene expression values for wild-type and mast cell engrafted mice. The black line displays the linear relationship between the two variables while the grey area indicates the 95% confidence interval. One outlier Il1b Δct value was excluded based on being over 7 standard deviations away from the rest of the data, and various outlier tests (Grubb's, Dixon, Chi-squared tests). n = 11-15.

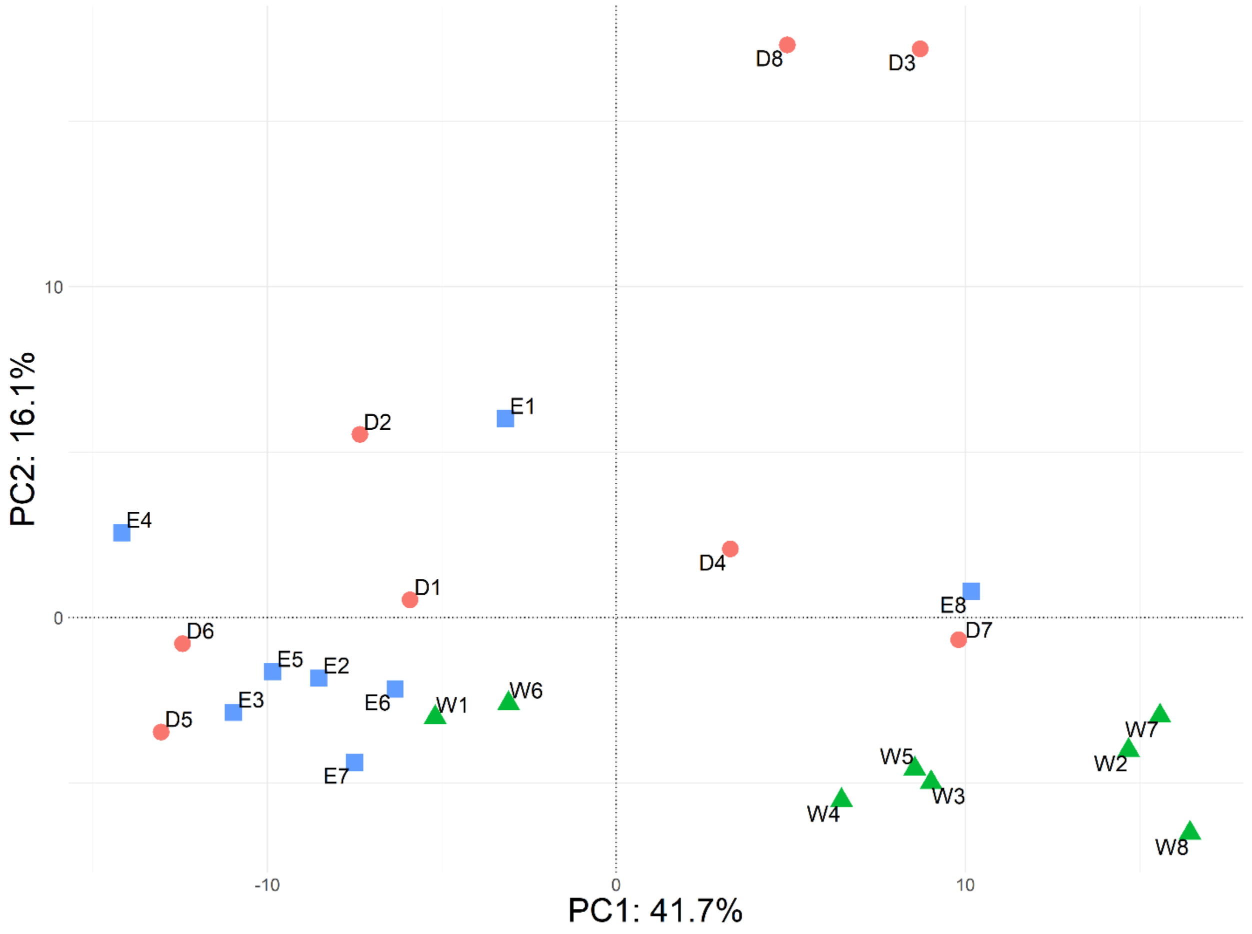

Supplementary Figure 15: Principal component analysis plot of Nanostring nCounter data. PC1 (41.7% of variance) versus PC2 (16.1% of variance) of the top 50% of assessed genes sorted by variance of their normalized gene expression based on the NanostringDiff package method. Points indicate wild-type, W1-W8 (green triangles), deficient, D1-D8 (red circles), and engrafted, E1-E8 (blue squares) samples. n = 8 per group.

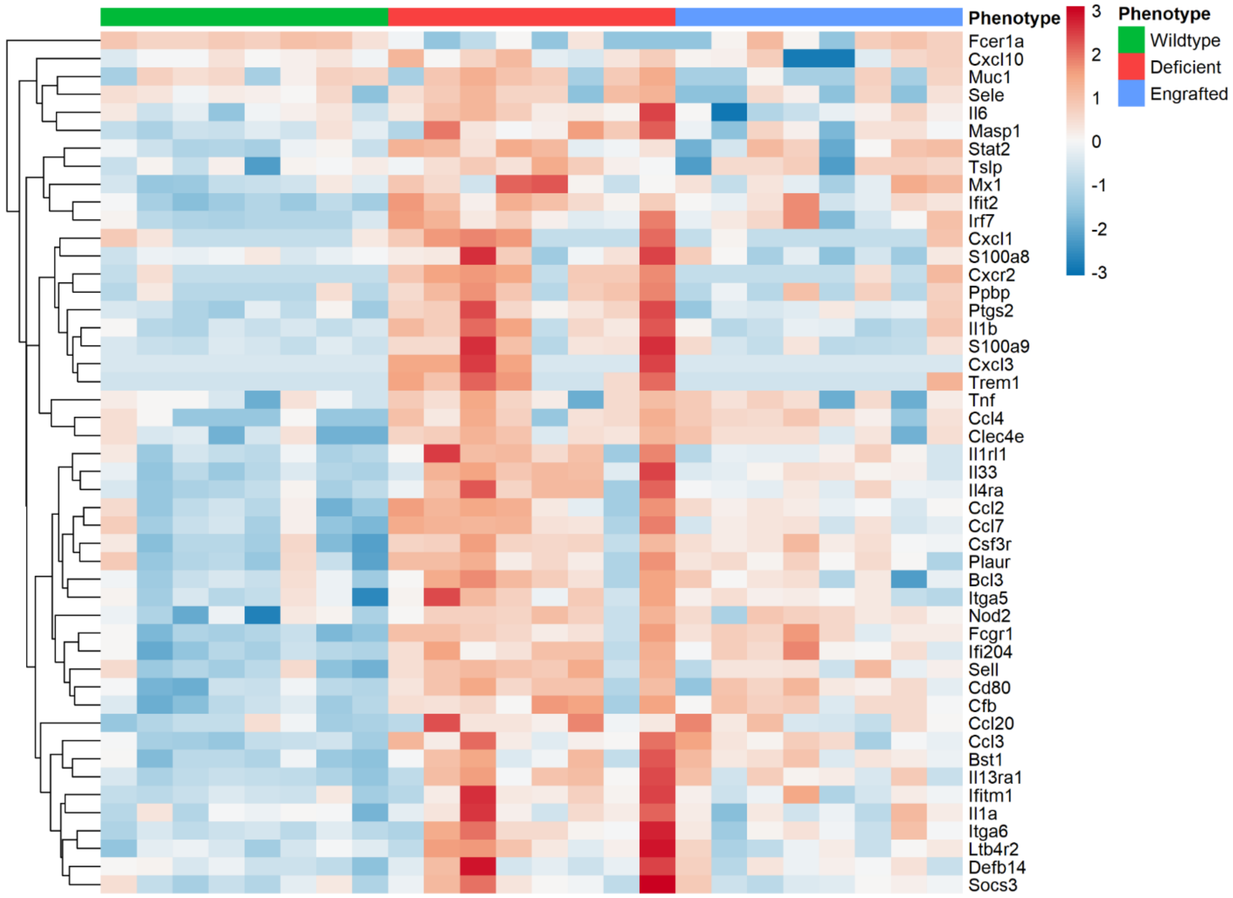

Supplementary Figure 16: Heatmap of wild-type, deficient, engrafted gene expression. Z-scores of differentially expressed genes based on q-value < 0.05 and log2 fold change ≥ 1. Nanostring nCounter gene expression data normalized based on the NanostringDiff package[2] method and displayed with pheatmap package[3] in R. Sample ID of wild-type (top green bar), deficient (top red bar), and engrafted mouse sample (top blue bar) for male mice at day 11 post-injection. Upregulated expression in red and downregulated in blue. n = 8 per group.

a

b

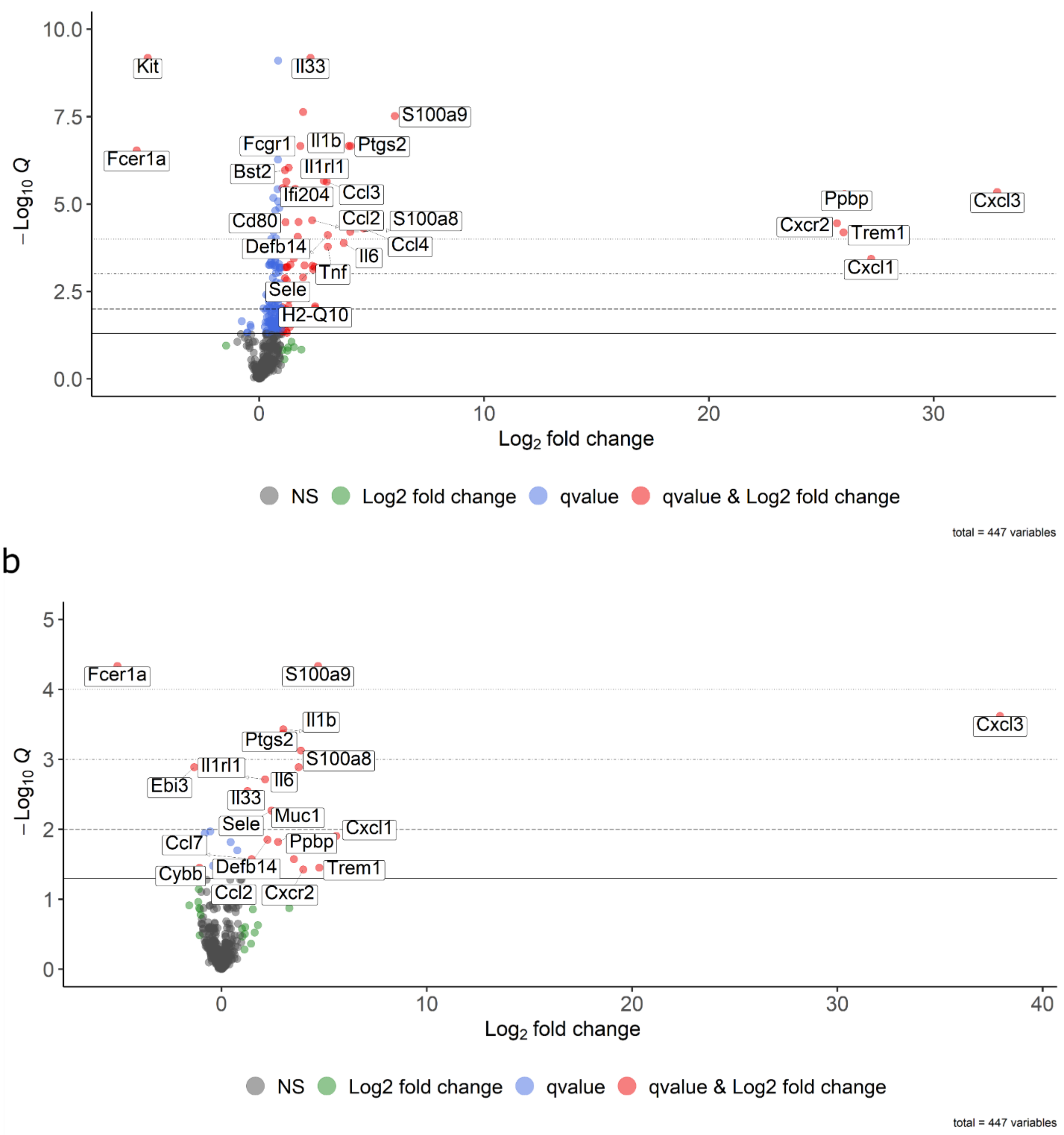

Supplementary Figure 17: Volcano plots of pairwise comparisons for deficient response. Top 25 ranked differentially expressed genes between deficient versus **(a)** wild-type and **(b)** engrafted sample groups with dots indicating false discovery rate less than 0.05 (blue, red), absolute fold change greater than 2 (green, red) or non-significant (black). Dashed lines indicating different significance thresholds. Results from n = 8 per group pooled from 2-3 independent experiments. (**q* < 0.05, ***q* < 0.01, ****q* < 0.001 and *****q* < 0.0001).

Supplementary Tables

Supplementary Table 1: Immune Related Primers

Gene	Forward Primer			Reverse Primer		
Arg1	5'	GAACACGGCAGTGGCTTTAAC	3'	5'	TGCTTAGTTCTGTCTGCTTTGC	3'
Ccl1	5'	AGAAAGAGCTTCCCCTGAAGTTT	3'	5'	TGAGGCGCAGCTTTCTCTACC	3'
Ccl8	5'	CCCCTTCGGGTGCTGAAAAG	3'	5'	TCACTGACCCACTTCTGTGTG	3'
Cd163	5'	GAGACACACGGAGCCATCAA	3'	5'	CGTTAGTGACAGCAGAGGCA	3'
Cd206	5'	GTGGACGCTCTAAGTGCCAT	3'	5'	GAATCTGACACCCAGCGGAA	3'
Cd86	5'	GACTTGAACAACCAGACTCCTG	3'	5'	ATCAGCAAGACTGTCACAAAGA	3'
Gapdh*	5’	CATCAAGAAGGTGGTGAAGC	3’	5’	GTTGTCATACCAGGAAATGAGC	3’
Ifng	5’	CGGCTGACTGAACTCAGATTG	3’	5’	CTGCAGCTCTGAATGTTTCTTAT	3’
Il13	5’	GAGCAACATCACACAAGACCAGA	3’	5’	GGCCAGGTCCACACTCCATA	3’
Il17	5’	AAGCTGGACCACCACATGAA	3’	5’	CCCTGAAAGTGAAGGGGCAG	3’
Il1b	5’	TGCCACCTTTTGACAGTGATG	3’	5’	TGATGTGCTGCTGCGAGATT	3’
Il1rn	5’	TCGGAGTACCTGTCATGCAAA	3’	5’	GCTTGCATCTTGCAGGGTCT	3’
Il33	5’	TCCAACTCCAAGATTTCCCCG	3’	5’	CAGTGCAGTAGACATGGCAGAA	3’
Il36	5’	AGGGCAAACCAACTTTGCAG	3’	5’	GAAGTGGAGCCCTCTATGCC	3’
Il38	5’	TGCAGGAATGTGCTCCCTTC	3’	5’	GGTCTAGGCCTCGGTTAGGA	3’
Il4	5’	GAGACTCTTTCGGGCTTTTCG	3’	5’	CAGTGATGTGGACTTGGACTC	3’
Il6	5’	TCCAGTTGCCTTCTTGGGAC	3’	5’	AGTCTCCTCTCCGGACTTGT	3’
Nos2	5’	CAGCTGGGCTGTACAAACCTT	3’	5’	CATTGGAAGTGAAGCGTTTCG	3’
Vegf	5’	GCACATAGAGAGAATGAGCTTCC	3’	5’	CTCCGCTCTGAACAAGGCT	3’

*Housekeeping Gene

Supplementary Table 2: Estrogen Receptor Primers

Gene	Forward Primer			Reverse Primer		
Esr1	5'	ACGCTCTGCCTTGATCACAC	3'	5'	CGAGTTACAGACTGGCTCCC	3'
Esr2	5'	GGTCCTGTGAAGGATGTAAGGC	3'	5'	TAACACTTGCGAAGTCGGCAGG	3'
Gapdh*	5'	CCCCTTCGGGTGCTGAAAAG	3'	5'	TCACTGACCCACTTCTGTGTG	3'

*Housekeeping Gene

Supplementary Table 3: Differentially Expressed Genes (q < 0.05)

Gene	Entrez ID	logFC	pvalue	qvalue	Comparison*
Bcl3	12051	0.96169649	0.0001054	0.00120808	Def:OG
Bst1	12182	1.11830749	0.00019134	0.00194389	Def:OG
Bst2	69550	0.53603854	0.00200392	0.01518222	Def:OG
Casp8	12370	0.41918401	6.3543E-05	0.0008354	Def:OG
Ccl2	20296	1.89680656	1.1491E-06	2.7034E-05	Def:OG
Ccl20	20297	1.24219447	0.00354698	0.02331616	Def:OG
Ccl3	20302	2.00175485	3.4153E-06	7.2697E-05	Def:OG
Ccl4	20303	3.21048965	8.3207E-06	0.00015497	Def:OG
Ccl7	20306	1.76375961	2.5692E-06	5.7422E-05	Def:OG
Cd24a	12484	0.60596165	0.00347673	0.02319547	Def:OG
Cd44	12505	0.48739085	9.7481E-05	0.00114668	Def:OG
Cd80	12519	0.71598694	0.00053364	0.00496955	Def:OG
Cd82	12521	-0.393306	0.00322404	0.02251787	Def:OG
Cebpb	12608	0.34623001	0.00605416	0.03707135	Def:OG
Cfb	14962	0.71651477	0.00013146	0.00143328	Def:OG
Clec4e	56619	2.65412174	0.00024281	0.00235949	Def:OG
Csf3r	12986	0.81387742	0.00096219	0.0081151	Def:OG
Cxcl1	14825	16.4075754	5.8602E-12	6.4495E-10	Def:OG
Cxcl10	15945	1.45361867	0.00563	0.03495291	Def:OG
Cxcl3	330122	35.376083	5.8602E-12	6.4495E-10	Def:OG
Cxcr2	12765	14.8415238	1.023E-07	3.5177E-06	Def:OG
Ddx58	230073	0.51773177	6.5056E-07	1.8175E-05	Def:OG
Defb14	244332	2.64540291	3.6017E-07	1.0733E-05	Def:OG
Ets1	23871	0.35799557	0.00261322	0.0191493	Def:OG
Fcer1a	14125	-5.2553055	5.7668E-09	2.8642E-07	Def:OG
Fcgr1	14129	0.96576671	0.00013777	0.00146626	Def:OG
Hif1a	15251	0.48622925	0.0006305	0.00563664	Def:OG
Ifi204	15951	0.89305581	0.00036756	0.00349577	Def:OG
Ifi35	70110	0.53015351	1.9779E-05	0.00034004	Def:OG
Ifih1	71586	0.41702725	0.00328498	0.02259054	Def:OG
Ifit2	15958	1.21375544	1.0835E-06	2.6907E-05	Def:OG
Ifitm1	68713	1.52645206	2.1919E-05	0.00036288	Def:OG
Il13ra1	16164	0.71734686	1.1495E-05	0.00020552	Def:OG
Il15ra	16169	0.55385611	0.00556318	0.03495291	Def:OG
Il1a	16175	0.78386753	0.00165205	0.01367528	Def:OG
Il1b	16176	3.49598248	6.5569E-12	6.4495E-10	Def:OG
Il1r1	16177	0.44052214	2.8016E-05	0.00043183	Def:OG
Il1rl1	17082	2.50021835	1.4854E-09	9.4853E-08	Def:OG
Il1rl2	107527	0.49937012	6.3963E-06	0.00012431	Def:OG
Il1rn	16181	0.38248162	3.7373E-06	7.5936E-05	Def:OG
Il33	77125	1.77680895	8.6571E-12	6.4495E-10	Def:OG

Gene	Entrez ID	logFC	pvalue	qvalue	Comparison*
Il4ra	16190	1.0450472	8.1054E-08	3.0192E-06	Def:OG
Il6	16193	3.76027532	1.8364E-08	7.4625E-07	Def:OG
Irak4	266632	0.39825528	0.00748803	0.04523173	Def:OG
Irf7	54123	1.07333276	0.00057388	0.00523522	Def:OG
Irgm1	15944	0.55583698	0.00378445	0.02451666	Def:OG
Itga5	16402	0.74098199	0.00017107	0.00177831	Def:OG
Itga6	16403	0.70216696	4.8938E-05	0.00066289	Def:OG
Itgb1	16412	0.32268046	0.00388622	0.02481629	Def:OG
Ltb4r1	16995	0.62696043	0.00186815	0.0147091	Def:OG
Ltb4r2	57260	0.88227927	3.2843E-05	0.00048936	Def:OG
Masp1	17174	1.08401375	4.3613E-05	0.00060923	Def:OG
Muc1	17829	2.38449534	6.7877E-05	0.00085887	Def:OG
Mx1	17857	0.69838439	0.00342332	0.0231852	Def:OG
Myd88	17874	0.52374775	6.9171E-05	0.00085887	Def:OG
Nfkb2	18034	0.4546497	0.00011362	0.00126975	Def:OG
Nfkbiz	80859	0.5066325	0.00090254	0.00791052	Def:OG
Nod2	257632	0.85099095	0.0030613	0.02172063	Def:OG
Pecam1	18613	0.53061448	0.00214905	0.0160104	Def:OG
Plaur	18793	0.86125994	0.00193183	0.01488841	Def:OG
Pml	18854	0.36928278	0.00290669	0.02095628	Def:OG
Ppbp	57349	14.7815329	1.1364E-08	5.0795E-07	Def:OG
Ptger4	19219	-0.3622475	0.00772546	0.04604376	Def:OG
Ptgs2	19225	3.52974826	7.3744E-12	6.4495E-10	Def:OG
S100a8	20201	3.95652448	3.4136E-09	1.9074E-07	Def:OG
S100a9	20202	5.36769599	5.8602E-12	6.4495E-10	Def:OG
Sele	20339	2.4500527	1.0485E-06	2.6907E-05	Def:OG
Sell	20343	1.7640824	0.00021234	0.00210923	Def:OG
Socs3	12702	1.10046276	8.4556E-05	0.00102153	Def:OG
Stat2	20847	0.81681082	0.00187566	0.0147091	Def:OG
Stat3	20848	0.51366144	3.531E-05	0.00050915	Def:OG
Thy1	21838	0.44147497	0.00092949	0.00799008	Def:OG
Tnf	21926	2.29002659	2.2793E-05	0.00036388	Def:OG
Tnfrsf4	22163	0.37668453	0.00789556	0.04643838	Def:OG
Trem1	58217	15.3797873	1.391E-07	4.4414E-06	Def:OG
Tslp	53603	1.50058921	0.00181989	0.0147091	Def:OG
Ahr	11622	0.56448308	0.01603709	0.03733635	Def:WT
Arhgdib	11857	0.74408796	0.00072	0.00357599	Def:WT
B2m	12010	0.73373826	0.00016113	0.00096035	Def:WT
Batf	53314	0.97972046	0.00098494	0.0043763	Def:WT
Bcap31	27061	0.19091078	0.00251655	0.00948378	Def:WT
Bcl3	12051	1.15886217	9.7302E-05	0.0006467	Def:WT
Bcl6	12053	0.43173393	0.00764583	0.02233781	Def:WT

Gene	Entrez ID	logFC	pvalue	qvalue	Comparison*
Bid	12122	0.60422495	0.00022318	0.00129557	Def:WT
Bst1	12182	1.74871239	1.9755E-06	3.2706E-05	Def:WT
Bst2	69550	1.1462163	2.9051E-08	1.0821E-06	Def:WT
Btk	12229	0.78436174	0.01469296	0.03550138	Def:WT
C1qa	12259	0.82884942	0.01279229	0.03212446	Def:WT
C1qb	12260	0.79389141	0.02096143	0.04732201	Def:WT
C1ra	50909	0.66905654	0.00782752	0.02257356	Def:WT
C1s1	50908	0.65018012	0.01103375	0.02867493	Def:WT
C3	12266	1.35668245	0.0131766	0.0327219	Def:WT
Card9	332579	0.84414501	0.00053554	0.00278358	Def:WT
Casp1	12362	0.47241089	7.2879E-05	0.00055215	Def:WT
Casp3	12367	0.24734896	0.01305265	0.03259517	Def:WT
Casp8	12370	0.62830888	3.2871E-07	6.6789E-06	Def:WT
Ackr2	59289	1.23142366	0.0220897	0.04816632	Def:WT
Ccl12	20293	1.19497619	0.00289159	0.01034034	Def:WT
Ccl2	20296	2.35594181	1.6847E-06	2.8964E-05	Def:WT
Ccl20	20297	1.95238181	0.00021455	0.00126191	Def:WT
Ccl3	20302	3.00319881	7.7016E-08	2.2951E-06	Def:WT
Ccl4	20303	4.64745749	3.3363E-06	4.9711E-05	Def:WT
Ccl6	20305	1.42542436	0.00581636	0.01793045	Def:WT
Ccl7	20306	2.05645603	1.1091E-05	0.00013046	Def:WT
Ccl9	20308	1.30645127	0.00973316	0.02636134	Def:WT
Ccr5	12774	1.11409207	0.01061065	0.02789977	Def:WT
Ccrl2	54199	2.01562382	7.685E-05	0.00057253	Def:WT
Cd109	235505	0.43685867	0.00495218	0.01604074	Def:WT
Cd14	12475	1.53795107	3.9396E-05	0.00035939	Def:WT
Cd244a	18106	1.03728775	0.00235414	0.00907154	Def:WT
Cd24a	12484	0.56175483	0.0218067	0.04778232	Def:WT
Cd274	60533	0.91238478	0.00762245	0.02233781	Def:WT
Cd44	12505	0.44733897	0.00235027	0.00907154	Def:WT
Cd53	12508	1.10907501	0.00978967	0.02636134	Def:WT
Cd80	12519	1.17588156	2.0751E-06	3.3128E-05	Def:WT
Cd81	12520	0.29819965	0.02172157	0.04778232	Def:WT
Cd86	12524	0.73473587	0.00297494	0.01055397	Def:WT
Adgre5	26364	0.73148057	0.01039545	0.02765931	Def:WT
Ceacam1	26365	0.68300415	0.00995719	0.02665188	Def:WT
Cfb	14962	1.20903654	7.2585E-08	2.2951E-06	Def:WT
Cfh	12628	0.81080907	0.01399221	0.03399195	Def:WT
Cfp	18636	1.08392498	0.00373887	0.01247219	Def:WT
Clec4e	56619	3.68941811	0.00012863	0.00078761	Def:WT
Clec5a	23845	0.89480075	0.00570523	0.01783383	Def:WT
Cmklr1	14747	0.64798096	0.00605902	0.01842437	Def:WT

Gene	Entrez ID	logFC	pvalue	qvalue	Comparison*
Csf1	12977	0.8973911	1.3141E-05	0.0001458	Def:WT
Csf2rb	12983	0.98938871	0.00261556	0.00958322	Def:WT
Csf3r	12986	1.29171056	1.3373E-05	0.0001458	Def:WT
Ctnnb1	12387	-0.3753829	0.01336422	0.03300445	Def:WT
Ctsc	13032	0.33712772	0.01843356	0.04247321	Def:WT
Cxcl1	14825	27.2214946	4.2216E-05	0.00037001	Def:WT
Cxcl10	15945	1.77015962	0.00624591	0.01886433	Def:WT
Cxcl13	55985	2.49354743	0.00259948	0.00958322	Def:WT
Cxcl3	330122	32.8314335	2.0417E-07	4.5632E-06	Def:WT
Cxcr2	12765	25.7009567	2.2868E-06	3.5248E-05	Def:WT
Ddx58	230073	0.84718634	5.3728E-12	8.0055E-10	Def:WT
Defb14	244332	3.04643048	5.8316E-06	7.6668E-05	Def:WT
Entpd1	12495	0.86670259	0.00014679	0.0008867	Def:WT
Ets1	23871	0.55380947	6.8971E-05	0.00054088	Def:WT
Fas	14102	0.78941453	0.00063847	0.00320668	Def:WT
Fasl	14103	1.19844211	0.01863119	0.04270843	Def:WT
Fcer1a	14125	-5.4444058	5.9105E-09	2.9355E-07	Def:WT
Fcer1g	14127	0.94957757	0.00260382	0.00958322	Def:WT
Fcgr1	14129	1.82930655	3.9407E-09	2.2018E-07	Def:WT
Fcgr4	246256	1.57436952	0.00078751	0.00382629	Def:WT
Fkbp5	14229	0.80061768	0.02127086	0.04754037	Def:WT
Gata3	14462	-0.5221126	0.02174266	0.04778232	Def:WT
Gm10499	69717	0.75305572	0.0003068	0.00171424	Def:WT
H2-K1	14972	0.542672	0.00042207	0.00227309	Def:WT
H2-Q10	15007	2.48664674	0.00215424	0.00851692	Def:WT
Icam1	15894	0.58431184	0.00087012	0.0040515	Def:WT
Ifi204	15951	1.6055303	1.3978E-07	3.6755E-06	Def:WT
Ifi35	70110	0.83646589	1.2039E-08	5.3814E-07	Def:WT
Ifih1	71586	0.84535918	4.3475E-07	8.4493E-06	Def:WT
Ifit2	15958	1.95861349	2.0999E-10	2.3466E-08	Def:WT
Ifitm1	68713	1.8946873	2.0976E-05	0.00019534	Def:WT
Ifnar1	15975	0.28940976	0.00313488	0.0110338	Def:WT
Ifnar2	15976	0.65556645	0.0021103	0.00842237	Def:WT
Ifngr1	15979	0.72656073	0.00891404	0.02474893	Def:WT
Ikbke	56489	0.74465874	7.9656E-05	0.0005743	Def:WT
Ikzf1	22778	0.98136072	0.00875728	0.02461953	Def:WT
Il10ra	16154	0.71577211	0.01782327	0.04127979	Def:WT
Il10rb	16155	0.63117993	0.0124124	0.03134656	Def:WT
Il13ra1	16164	1.03084143	1.2727E-07	3.5556E-06	Def:WT
Il15ra	16169	0.91312133	0.00010964	0.00069027	Def:WT
Il16	16170	0.60970361	0.01520224	0.03624722	Def:WT
Il17ra	16172	0.48880153	0.00095048	0.00433537	Def:WT

Gene	Entrez ID	logFC	pvalue	qvalue	Comparison*
Il18	16173	0.54204873	0.0121307	0.03098528	Def:WT
Il18rap	16174	1.36202952	0.00664958	0.01994873	Def:WT
Il1a	16175	0.87760667	0.00285454	0.01029015	Def:WT
Il1b	16176	3.98437347	3.9322E-09	2.2018E-07	Def:WT
Il1r1	16177	0.49558386	5.786E-05	0.00047895	Def:WT
Il1rap	16180	0.3687875	0.02229761	0.04838365	Def:WT
Il1rl1	17082	2.87567927	6.3562E-08	2.1855E-06	Def:WT
Il1rl2	107527	0.55496161	1.9456E-05	0.00018504	Def:WT
Il1rn	16181	0.4171664	1.5351E-05	0.00015248	Def:WT
Il2rg	16186	0.8864661	0.00119465	0.00513472	Def:WT
Il33	77125	2.28437285	2.9817E-12	6.6642E-10	Def:WT
Il4ra	16190	1.31811485	2.2473E-08	9.1322E-07	Def:WT
Il6	16193	3.75964139	1.1457E-05	0.00013132	Def:WT
Il6ra	16194	0.70047987	0.01057268	0.02789977	Def:WT
Il6st	16195	0.43193261	0.00935909	0.02566504	Def:WT
Irak2	108960	0.53555608	8.2775E-06	0.0001	Def:WT
Irak3	73914	0.67598444	0.00117042	0.00507939	Def:WT
Irak4	266632	0.71024537	4.9622E-05	0.00042656	Def:WT
Irf5	27056	0.65679115	0.00732106	0.02167228	Def:WT
Irf7	54123	1.71775196	6.7991E-06	8.6835E-05	Def:WT
Irf8	15900	0.8690524	0.00514048	0.01653091	Def:WT
Irgm1	15944	1.20037553	1.6096E-07	3.7869E-06	Def:WT
Itga4	16401	0.8094452	0.00098104	0.0043763	Def:WT
Itga5	16402	0.91101173	0.00010101	0.00064725	Def:WT
Itga6	16403	0.89330619	1.4194E-05	0.00014726	Def:WT
Itgal	16408	0.79410903	0.00134936	0.00569022	Def:WT
Itgam	16409	1.16493811	0.00440876	0.01457127	Def:WT
Itgb1	16412	0.53003901	5.1008E-05	0.0004302	Def:WT
Jak1	16451	0.65924119	1.4288E-05	0.00014726	Def:WT
Jak2	16452	0.44926921	0.001598	0.00661395	Def:WT
Jak3	16453	0.65026171	0.0106753	0.02790562	Def:WT
Kit	16590	-4.9558404	2.9817E-12	6.6642E-10	Def:WT
Lcp2	16822	1.32940849	0.00126113	0.00536881	Def:WT
Pirb	18733	1.14403175	0.00023143	0.00132625	Def:WT
Lilrb4a	14728	1.03478993	0.00575915	0.01787735	Def:WT
Litaf	56722	0.51827679	0.00695844	0.02073614	Def:WT
Ltb4r1	16995	0.79560927	0.00083028	0.00394824	Def:WT
Ltb4r2	57260	0.9657837	0.00010136	0.00064725	Def:WT
Ltbr	17000	0.31549049	0.00083928	0.00394902	Def:WT
Ly96	17087	0.72330096	4.0763E-05	0.00036442	Def:WT
Masp1	17174	1.25132012	9.0271E-05	0.0006222	Def:WT
Msr1	20288	1.30256347	0.0021721	0.00851692	Def:WT

Gene	Entrez ID	logFC	pvalue	qvalue	Comparison*
Muc1	17829	2.01435614	0.00360269	0.01229316	Def:WT
Mx1	17857	0.99363692	0.00047026	0.00247302	Def:WT
Myd88	17874	0.81523006	1.5428E-07	3.7869E-06	Def:WT
Ncf4	17972	0.86543997	0.01524492	0.03624722	Def:WT
Nfatc1	18018	0.71225062	9.503E-05	0.00064361	Def:WT
Nfatc2	18019	0.55498507	0.00368462	0.01238366	Def:WT
Nfkb1	18033	0.43732891	7.1734E-05	0.00055215	Def:WT
Nfkb2	18034	0.63548606	4.4024E-06	6.2726E-05	Def:WT
Nfkbia	18035	0.54538593	0.00476453	0.01554557	Def:WT
Nfkbiz	80859	0.89627854	6.9011E-07	1.2853E-05	Def:WT
Nod2	257632	1.38639349	6.7525E-05	0.000539	Def:WT
Notch1	18128	-0.533063	0.02166384	0.04778232	Def:WT
Nox4	50490	0.99090246	0.00566374	0.01782881	Def:WT
Pdgfrb	18596	0.48574117	0.02073456	0.04704745	Def:WT
Pecam1	18613	0.66702852	0.00107554	0.0047134	Def:WT
Plau	18792	0.72514788	0.0148094	0.03559033	Def:WT
Plaur	18793	1.1781171	0.00041479	0.00226109	Def:WT
Pml	18854	0.63024412	1.4496E-05	0.00014726	Def:WT
Pou2f2	18987	0.71405486	0.00203624	0.0082	Def:WT
Ppbp	57349	26.0377163	2.4465E-07	5.2076E-06	Def:WT
Psmb10	19171	0.75852374	0.00242369	0.00925973	Def:WT
Psmb9	16912	0.62024577	0.00840313	0.02392484	Def:WT
Ptafr	19204	0.91317796	0.00443332	0.01457127	Def:WT
Ptger4	19219	-0.3897851	0.01110418	0.02869115	Def:WT
Ptgs2	19225	4.07435572	3.1602E-09	2.2018E-07	Def:WT
Ptpn22	19260	0.98953642	0.00941626	0.02566504	Def:WT
Ptpn6	15170	0.74282868	7.7544E-06	9.6284E-05	Def:WT
Ptprc	19264	0.7775139	0.01571891	0.03710537	Def:WT
Rela	19697	0.34589487	0.00268787	0.0097681	Def:WT
Relb	19698	0.51016166	0.00252476	0.00948378	Def:WT
S100a8	20201	4.04777647	4.4905E-06	6.2726E-05	Def:WT
S100a9	20202	6.02911858	3.4166E-10	3.0544E-08	Def:WT
Sele	20339	2.46560872	9.0476E-05	0.0006222	Def:WT
Sell	20343	2.36721255	8.2428E-05	0.00058484	Def:WT
Smad3	17127	0.28600294	0.00861511	0.02437312	Def:WT
Socs3	12702	1.22837364	0.00026994	0.00152741	Def:WT
Stat1	20846	0.87899754	6.3858E-05	0.00051899	Def:WT
Stat2	20847	1.23039835	9.838E-05	0.0006467	Def:WT
Stat3	20848	0.7213516	8.6241E-07	1.542E-05	Def:WT
Stat5a	20850	0.56132111	0.00181245	0.00736516	Def:WT
Stat5b	20851	0.45350613	0.02113319	0.04747004	Def:WT
Stat6	20852	0.4019469	0.00092076	0.00424311	Def:WT

Gene	Entrez ID	logFC	pvalue	qvalue	Comparison*
Syk	20963	0.85443908	0.00345461	0.01187853	Def:WT
Tagap	72536	1.05732642	0.01956431	0.04461861	Def:WT
Tap1	21354	0.61954619	0.00056414	0.00289853	Def:WT
Tapbp	21356	0.4942555	0.00338005	0.01179488	Def:WT
Tbk1	56480	0.37132498	0.00837889	0.02392484	Def:WT
Tcf7	21414	-0.7769312	0.00782532	0.02257356	Def:WT
Tgfb1	21803	0.55056093	0.00883522	0.02468339	Def:WT
Tgfbi	21810	0.92423954	0.00075132	0.00369053	Def:WT
Tgfbr2	21813	0.71963592	0.00367852	0.01238366	Def:WT
Thy1	21838	0.515858	0.00098883	0.0043763	Def:WT
Tirap	117149	0.440983	0.00144998	0.00605738	Def:WT
Tlr1	21897	1.05679169	0.00082931	0.00394824	Def:WT
Tlr2	24088	1.05054955	0.00044102	0.00234684	Def:WT
Tlr3	142980	0.46222935	0.00921363	0.02542279	Def:WT
Tlr4	21898	0.90461966	0.0051927	0.01657956	Def:WT
Tlr5	53791	0.67047182	0.00600269	0.0183781	Def:WT
Tlr8	170744	0.98090176	0.01577186	0.03710537	Def:WT
Tlr9	81897	0.83962399	0.00167537	0.00687057	Def:WT
Sting1	72512	0.57733489	0.01383827	0.03380167	Def:WT
Tnf	21926	3.04849334	1.6941E-05	0.00016462	Def:WT
Tnfaip3	21929	0.69645709	7.8314E-05	0.00057387	Def:WT
Tnfrsf11a	21934	0.94127559	0.000333	0.00183769	Def:WT
Tnfrsf14	230979	0.64009793	0.01358461	0.03336441	Def:WT
Tnfrsf1b	21938	0.8156123	0.01600547	0.03733635	Def:WT
Tnfrsf4	22163	0.56630022	0.0005953	0.00302387	Def:WT
Tnfsf14	50930	1.25105424	0.00340389	0.01179488	Def:WT
Trem1	58217	25.9973372	4.7768E-06	6.4704E-05	Def:WT
Tslp	53603	2.40179304	0.00011788	0.00073185	Def:WT
Vcam1	22329	0.81962002	0.0122699	0.03116276	Def:WT
Xbp1	22433	0.33997335	0.00552938	0.01752931	Def:WT
Zeb1	21417	0.5506855	0.01125763	0.02892046	Def:WT
Ccl2	20296	1.43767131	0.00262108	0.03905403	Def:Eng
Ccl7	20306	1.47106319	0.00137369	0.02669728	Def:Eng
Cd44	12505	0.52744274	0.00034548	0.01072949	Def:Eng
Cd82	12521	-0.5443381	0.0003527	0.01072949	Def:Eng
Cxcl1	14825	5.59365619	0.00047244	0.01242227	Def:Eng
Cxcl3	330122	37.9207325	1.609E-06	0.00023974	Def:Eng
Cxcr2	12765	3.98209086	0.00243273	0.03749759	Def:Eng
Cybb	13058	-1.0707071	0.00220247	0.03548621	Def:Eng
Defb14	244332	2.24437535	0.0005684	0.01411526	Def:Eng
Ebi3	50498	-1.3229136	2.3196E-05	0.00129608	Def:Eng
Fcer1a	14125	-5.0662052	2.0642E-07	4.6135E-05	Def:Eng

Gene	Entrez ID	logFC	pvalue	qvalue	Comparison*
Hif1a	15251	0.60121988	0.00034343	0.01072949	Def:Eng
Il1b	16176	3.00759148	3.3444E-06	0.00037373	Def:Eng
Il1r1	16177	0.38546041	0.00186245	0.03330055	Def:Eng
Il1rl1	17082	2.12475742	3.8798E-05	0.00192697	Def:Eng
Il1rl2	107527	0.44377864	0.00068523	0.01531481	Def:Eng
Il1rn	16181	0.34779683	0.00036005	0.01072949	Def:Eng
Il33	77125	1.26924505	6.3377E-05	0.00283297	Def:Eng
Il4ra	16190	0.77197954	0.00093636	0.01993114	Def:Eng
Il6	16193	3.76090926	2.2249E-05	0.00129608	Def:Eng
Ltb4r2	57260	0.79877484	0.00222285	0.03548621	Def:Eng
Muc1	17829	2.75463454	0.00064569	0.01519062	Def:Eng
Pou2f2	18987	-0.8066599	0.00040863	0.01141619	Def:Eng
Ppbp	57349	3.52534944	0.00135651	0.02669728	Def:Eng
Ptgs2	19225	2.9851408	4.8123E-06	0.00043022	Def:Eng
S100a8	20201	3.8652725	1.0062E-05	0.00074965	Def:Eng
S100a9	20202	4.7062734	1.8056E-07	4.6135E-05	Def:Eng
Sele	20339	2.43449668	0.00013296	0.00540305	Def:Eng
Tgfbr1	21812	-0.4098002	0.00185108	0.03330055	Def:Eng
Trem1	58217	4.76223741	0.00215921	0.03548621	Def:Eng
Ahr	11622	0.75430395	0.00134904	0.00469158	Eng:WT
App	11820	0.23703065	0.02482129	0.04803082	Eng:WT
Arhgdib	11857	0.96346057	1.2918E-05	0.00013428	Eng:WT
B2m	12010	0.97422275	6.0437E-07	1.3508E-05	Eng:WT
Batf	53314	1.13809924	0.00014528	0.00083256	Eng:WT
Bcap31	27061	0.19787806	0.00227327	0.0069126	Eng:WT
Bcl6	12053	0.7123485	1.1887E-05	0.00012652	Eng:WT
Bid	12122	0.60202523	0.0002872	0.00138043	Eng:WT
Blnk	17060	0.83702215	0.00018749	0.00099773	Eng:WT
Bst1	12182	1.2608098	0.00053732	0.00226588	Eng:WT
Bst2	69550	1.22035553	3.8665E-09	3.4566E-07	Eng:WT
Btk	12229	1.06032712	0.00103541	0.00388932	Eng:WT
C1qa	12259	1.29929041	0.00011131	0.00065471	Eng:WT
C1qb	12260	1.40080189	5.7401E-05	0.00038296	Eng:WT
C1ra	50909	0.7952419	0.00161792	0.00529177	Eng:WT
C1s1	50908	0.95679634	0.00019755	0.0010268	Eng:WT
C2	12263	0.76632443	0.0062554	0.01487321	Eng:WT
Card9	332579	0.93121384	0.00015753	0.00086935	Eng:WT
Casp1	12362	0.60850419	3.6781E-07	8.6533E-06	Eng:WT
Casp8	12370	0.41824974	0.00071363	0.00284814	Eng:WT
Ackr2	59289	1.95268597	0.00034097	0.00158763	Eng:WT
Ccl12	20293	0.93787497	0.01898689	0.03829113	Eng:WT
Ccl20	20297	1.42037469	0.00700219	0.01646978	Eng:WT

Gene	Entrez ID	logFC	pvalue	qvalue	Comparison*
Ccl3	20302	2.00288794	0.00020853	0.0010714	Eng:WT
Ccl4	20303	2.87393567	0.00243621	0.00720167	Eng:WT
Ccl6	20305	1.21427825	0.01819468	0.03722258	Eng:WT
Ccr2	12772	1.08098323	0.00152464	0.00504826	Eng:WT
Ackr4	252837	0.75385576	0.00854289	0.01928621	Eng:WT
Ccrl2	54199	2.32585551	5.8477E-06	7.0646E-05	Eng:WT
Cd109	235505	0.46912599	0.00258412	0.00731115	Eng:WT
Cd14	12475	1.50064156	6.0068E-05	0.00039486	Eng:WT
Cd22	12483	0.92564342	0.00488959	0.0122789	Eng:WT
Cd244a	18106	0.90080631	0.00850561	0.01928621	Eng:WT
Cd274	60533	1.03926031	0.00246572	0.00720378	Eng:WT
Cd36	12491	1.02615286	0.0034856	0.00932971	Eng:WT
Cd4	12504	0.81216212	0.01739972	0.03609024	Eng:WT
Cd40	21939	0.68367149	0.02329629	0.04565257	Eng:WT
Cd48	12506	1.07236617	0.00190481	0.0060296	Eng:WT
Cd53	12508	0.99236886	0.02134014	0.04239574	Eng:WT
Cd74	16149	1.32088967	0.00481794	0.01216735	Eng:WT
Cd79b	15985	1.77036294	0.00023698	0.00119023	Eng:WT
Cd80	12519	0.91978924	0.00025004	0.00122819	Eng:WT
Cd81	12520	0.36684126	0.00481129	0.01216735	Eng:WT
Cd83	12522	1.18306563	0.0080006	0.01824628	Eng:WT
Cd86	12524	1.2279971	8.7632E-07	1.6277E-05	Eng:WT
Adgre5	26364	0.77051964	0.00703742	0.01646978	Eng:WT
Ceacam1	26365	0.6372064	0.01852808	0.03764569	Eng:WT
Cfb	14962	0.98504354	1.1121E-05	0.00012427	Eng:WT
Cfd	11537	1.34300245	0.00771106	0.01767612	Eng:WT
Cfh	12628	1.03167478	0.00183541	0.00590238	Eng:WT
Cfp	18636	1.07396095	0.00407297	0.0106469	Eng:WT
Ciita	12265	1.02723603	0.01306236	0.02827778	Eng:WT
Clec4e	56619	2.07059273	0.02260677	0.04451642	Eng:WT
Clec5a	23845	1.10957359	0.00068869	0.00277338	Eng:WT
Cmklr1	14747	1.02875593	1.4987E-05	0.00015226	Eng:WT
Crlf2	57914	1.13277333	0.00041817	0.00185071	Eng:WT
Csf1	12977	0.96126471	3.2412E-06	4.2613E-05	Eng:WT
Csf1r	12978	1.18147382	0.00025528	0.00124034	Eng:WT
Csf2rb	12983	0.8987904	0.00622525	0.01487321	Eng:WT
Csf3r	12986	0.95566629	0.00128965	0.00461178	Eng:WT
Ctnnb1	12387	-0.4937133	0.0011572	0.00423991	Eng:WT
Ctsc	13032	0.51082616	0.00036504	0.00166501	Eng:WT
Ctss	13040	1.53050252	0.00209181	0.00649332	Eng:WT
Cul9	78309	0.6433325	0.0056978	0.01376712	Eng:WT
Cx3cr1	13051	1.88446856	4.1068E-06	5.245E-05	Eng:WT

Gene	Entrez ID	logFC	pvalue	qvalue	Comparison*
Cxcl12	20315	0.65528387	3.5986E-05	0.00027712	Eng:WT
Cxcl13	55985	2.96611795	0.00044096	0.00193243	Eng:WT
Cxcl9	17329	1.79456854	0.00494573	0.01228189	Eng:WT
Cxcr2	12765	21.7188659	6.1062E-15	1.3647E-12	Eng:WT
Cxcr3	12766	0.86041	0.00654275	0.01547413	Eng:WT
Cxcr4	12767	0.74395624	0.00244889	0.00720167	Eng:WT
Cybb	13058	1.70493756	1.6116E-06	2.5728E-05	Eng:WT
Ddx58	230073	0.65890913	1.0484E-07	3.9052E-06	Eng:WT
Ebi3	50498	1.73784772	3.9003E-08	1.7435E-06	Eng:WT
Adgre1	13733	1.68809653	3.2416E-05	0.00026117	Eng:WT
Entpd1	12495	1.20906427	1.4642E-07	4.6749E-06	Eng:WT
Ets1	23871	0.39162781	0.00516658	0.01268935	Eng:WT
Fas	14102	0.66240816	0.00475044	0.01216735	Eng:WT
Fcamr	64435	-0.9199033	0.0047944	0.01216735	Eng:WT
Fcer1g	14127	1.3212003	3.2463E-05	0.00026117	Eng:WT
Fcgr1	14129	1.72707969	2.5359E-08	1.2595E-06	Eng:WT
Fcgr2b	14130	1.22210907	0.00286752	0.00791224	Eng:WT
Fcgr3	14131	1.41340529	0.00053677	0.00226588	Eng:WT
Fcgr4	246256	1.82825872	0.00010921	0.00065091	Eng:WT
Fcgrt	14132	0.94860189	0.00162186	0.00529177	Eng:WT
Fkbp5	14229	1.11004084	0.00150906	0.00504826	Eng:WT
Gm10499	69717	0.85595691	5.4245E-05	0.00036738	Eng:WT
Gpr183	321019	1.0275173	0.00258426	0.00731115	Eng:WT
H2-Aa	14960	1.4669812	0.00137819	0.00470269	Eng:WT
H2-Ab1	14961	1.30272773	0.00295674	0.00805891	Eng:WT
H2-DMa	14998	1.41892721	0.00133821	0.00469158	Eng:WT
H2-DMb2	15000	1.34895752	0.01215528	0.02663436	Eng:WT
H2-Eb1	14969	1.25211547	0.00569521	0.01376712	Eng:WT
H2-K1	14972	0.76572993	7.0802E-07	1.4386E-05	Eng:WT
H2-Q10	15007	2.52891983	0.00188644	0.00602312	Eng:WT
Hcst	23900	1.39403329	0.01446411	0.03093521	Eng:WT
Hfe	15216	0.9101743	0.00612682	0.01472412	Eng:WT
Hlx	15284	0.60834606	0.01498651	0.03189986	Eng:WT
Icam1	15894	0.56409713	0.00144451	0.00489164	Eng:WT
Ifi204	15951	1.42494896	2.7192E-06	3.7984E-05	Eng:WT
Ifi35	70110	0.61262477	3.2719E-05	0.00026117	Eng:WT
Ifih1	71586	0.85666387	3.3766E-07	8.3851E-06	Eng:WT
Ifit2	15958	1.4897161	1.1104E-06	1.8383E-05	Eng:WT
Ifnar1	15975	0.48404448	8.8792E-07	1.6277E-05	Eng:WT
Ifnar2	15976	0.99893665	3.2324E-06	4.2613E-05	Eng:WT
Ifngr1	15979	1.1701267	2.9423E-05	0.00025292	Eng:WT
Ifngr2	15980	0.58047192	0.00010849	0.00065091	Eng:WT

Gene	Entrez ID	logFC	pvalue	qvalue	Comparison*
Ikbke	56489	0.93046701	9.585E-07	1.6479E-05	Eng:WT
Ikzf1	22778	1.06242789	0.0047901	0.01216735	Eng:WT
Ikzf2	22779	0.89958417	0.0024925	0.00723473	Eng:WT
Il10ra	16154	1.35952885	8.6266E-06	0.00010148	Eng:WT
Il10rb	16155	1.02790002	5.2204E-05	0.00036461	Eng:WT
Il13ra1	16164	0.62698914	0.001247	0.00449523	Eng:WT
Il15	16168	0.65940967	0.01578765	0.03344587	Eng:WT
Il15ra	16169	0.71853044	0.00280918	0.00779939	Eng:WT
Il16	16170	1.33463179	1.4225E-07	4.6749E-06	Eng:WT
Il17ra	16172	0.50952526	0.00062972	0.00260633	Eng:WT
Il18r1	16182	1.15951823	0.00033445	0.00157366	Eng:WT
Il1r2	16178	0.66071896	0.02129115	0.04239574	Eng:WT
Il22ra2	237310	2.09737618	0.00087191	0.00333115	Eng:WT
Il2rg	16186	0.83352257	0.00242767	0.00720167	Eng:WT
Il33	77125	1.01512779	0.00135395	0.00469158	Eng:WT
Il4ra	16190	0.54613532	0.01901707	0.03829113	Eng:WT
Il6ra	16194	1.12054153	4.9127E-05	0.00034857	Eng:WT
Il7r	16197	1.37017697	0.00533726	0.0130369	Eng:WT
Il9	16198	-3.3368729	0.01353639	0.02909021	Eng:WT
Irak1	16179	0.30984556	0.01653577	0.03470183	Eng:WT
Irak2	108960	0.68782514	1.5206E-08	9.7102E-07	Eng:WT
Irak3	73914	1.07409303	3.0656E-07	8.0606E-06	Eng:WT
Irak4	266632	0.62398018	0.00037344	0.00166928	Eng:WT
Irf1	16362	0.82930717	3.3952E-05	0.00026625	Eng:WT
Irf5	27056	1.03239076	2.8027E-05	0.00025056	Eng:WT
Irf7	54123	1.28883841	0.00065351	0.00267998	Eng:WT
Irf8	15900	1.48074606	2.4808E-06	3.5772E-05	Eng:WT
Irgm1	15944	1.28907709	2.0624E-08	1.1524E-06	Eng:WT
Itga4	16401	1.2725377	2.7751E-07	7.753E-06	Eng:WT
Itgal	16408	0.94512192	0.00015226	0.00086154	Eng:WT
Itgam	16409	1.24827856	0.00232593	0.00702494	Eng:WT
Itgb1	16412	0.41471711	0.00151792	0.00504826	Eng:WT
Itgb2	16414	1.22332848	0.00317641	0.00860519	Eng:WT
Jak1	16451	0.69694869	4.6263E-06	5.7443E-05	Eng:WT
Jak2	16452	0.46538118	0.0011338	0.00418848	Eng:WT
Kit	16590	-5.7186637	6.1062E-15	1.3647E-12	Eng:WT
Klrc1	16641	1.32485128	0.01076894	0.02394883	Eng:WT
Klrd1	16643	0.97436829	0.02226736	0.04404208	Eng:WT
Klrk1	27007	1.03460339	0.00712796	0.01659478	Eng:WT
Lair1	52855	1.51589578	0.00132997	0.00469158	Eng:WT
Lcp2	16822	1.42963036	0.00059577	0.00248887	Eng:WT
Pirb	18733	1.2587462	5.3937E-05	0.00036738	Eng:WT

Gene	Entrez ID	logFC	pvalue	qvalue	Comparison*
Lilrb4a	14728	1.48597448	8.7345E-05	0.00055663	Eng:WT
Litaf	56722	0.7497895	0.00010029	0.00062266	Eng:WT
Ltb	16994	1.00359888	0.0027642	0.00772248	Eng:WT
Ltbr	17000	0.21551707	0.02413681	0.04690936	Eng:WT
Ly86	17084	1.49372747	4.5455E-05	0.00033309	Eng:WT
Ly96	17087	0.87040401	9.1033E-07	1.6277E-05	Eng:WT
Map4k1	26411	1.19085122	0.01816964	0.03722258	Eng:WT
Mapk14	26416	0.33958452	0.00493417	0.01228189	Eng:WT
Mapkapk2	17164	0.41444371	1.5936E-05	0.0001583	Eng:WT
Mbp	17196	1.74488851	0.0008643	0.00333052	Eng:WT
Msr1	20288	1.40085145	0.001009	0.00382224	Eng:WT
Myd88	17874	0.58296464	0.00019412	0.00102082	Eng:WT
Ncf4	17972	1.14844744	0.0013647	0.00469248	Eng:WT
Nfatc1	18018	0.67888846	0.00022288	0.00113214	Eng:WT
Nfatc2	18019	0.91478239	1.9518E-06	2.9186E-05	Eng:WT
Nfkb1	18033	0.46244643	2.938E-05	0.00025292	Eng:WT
Nfkb2	18034	0.36167273	0.00925163	0.02067739	Eng:WT
Nfkbia	18035	0.7758751	6.3557E-05	0.00041174	Eng:WT
Nfkbiz	80859	0.77929209	1.6614E-05	0.00016145	Eng:WT
Nod2	257632	1.07080509	0.00276368	0.00772248	Eng:WT
Nos2	18126	-0.8623067	0.023388	0.04565257	Eng:WT
Notch1	18128	-0.5878474	0.01148845	0.02542245	Eng:WT
Nox4	50490	1.05601213	0.00331706	0.00893208	Eng:WT
Pdcd2	18567	-0.2881732	0.01706521	0.03564555	Eng:WT
Pdgfb	18591	0.60536365	0.00191544	0.0060296	Eng:WT
Pla2g2e	26970	-1.1309751	0.02078589	0.04166498	Eng:WT
Pml	18854	0.52192268	0.00035813	0.00165037	Eng:WT
Pou2f2	18987	1.52071474	7.3573E-11	8.2218E-09	Eng:WT
Pparg	19016	0.82341688	0.0005327	0.00226588	Eng:WT
Ppbp	57349	22.5123668	0.00401621	0.01056027	Eng:WT
Prdm1	12142	-0.5899911	0.01309508	0.02827778	Eng:WT
Prkcd	18753	0.64610747	0.00072398	0.00286387	Eng:WT
Psmb10	19171	0.94917712	0.00016053	0.00087508	Eng:WT
Psmb5	19173	-0.2581162	0.01277936	0.02786524	Eng:WT
Psmb9	16912	0.92884507	8.8413E-05	0.00055663	Eng:WT
Psmd7	17463	-0.3299275	0.00215347	0.00659316	Eng:WT
Ptafr	19204	1.38848425	1.8555E-05	0.00017647	Eng:WT
Ptpn22	19260	1.44364995	0.00017695	0.00095299	Eng:WT
Ptpn6	15170	1.15948851	4.3878E-12	6.5379E-10	Eng:WT
Ptprc	19264	1.13290796	0.00047335	0.00205426	Eng:WT
Rela	19697	0.42550965	0.0002412	0.00119796	Eng:WT
Relb	19698	0.70936354	3.0038E-05	0.00025334	Eng:WT

Gene	Entrez ID	logFC	pvalue	qvalue	Comparison*
Rorc	19885	0.79172026	0.00734812	0.01701871	Eng:WT
Ski	20481	0.35751956	0.01743958	0.03609024	Eng:WT
Slamf7	75345	1.63130228	9.3585E-06	0.00010726	Eng:WT
Smad5	17129	-0.3324006	0.01630312	0.03437498	Eng:WT
Spn	20737	1.35890761	0.00066943	0.00272034	Eng:WT
Stat1	20846	0.96718896	1.1453E-05	0.00012487	Eng:WT
Stat2	20847	0.82717505	0.00919682	0.02065818	Eng:WT
Stat3	20848	0.41538031	0.00456291	0.01185825	Eng:WT
Stat5a	20850	0.42736267	0.01823657	0.03722258	Eng:WT
Stat5b	20851	0.61030802	0.00197919	0.00618671	Eng:WT
Stat6	20852	0.49474701	4.7808E-05	0.00034468	Eng:WT
Syk	20963	0.98319764	0.00079158	0.00310385	Eng:WT
Tagap	72536	2.1877319	1.9588E-06	2.9186E-05	Eng:WT
Tap1	21354	0.89872064	6.4836E-07	1.3801E-05	Eng:WT
Tapbp	21356	0.69851067	3.6577E-05	0.00027712	Eng:WT
Tbk1	56480	0.43550131	0.00214682	0.00659316	Eng:WT
Tcf7	21414	-0.886617	0.0025646	0.00731115	Eng:WT
Tgfb1	21803	0.8053235	0.00013587	0.00078876	Eng:WT
Tgfbi	21810	0.97758756	0.00037077	0.00166928	Eng:WT
Tgfbr1	21812	0.56033591	2.0837E-05	0.00019405	Eng:WT
Tgfbr2	21813	0.75184592	0.00243069	0.00720167	Eng:WT
Tlr1	21897	1.31243876	3.7282E-05	0.00027775	Eng:WT
Tlr2	24088	1.6356949	6.6107E-08	2.6864E-06	Eng:WT
Tlr3	142980	0.47173881	0.01167659	0.02571152	Eng:WT
Tlr4	21898	0.97972481	0.00252705	0.00728769	Eng:WT
Tlr8	170744	1.33652875	0.00110021	0.00409828	Eng:WT
Tlr9	81897	1.55966813	8.4923E-09	6.3267E-07	Eng:WT
Tnfaip3	21929	0.74698313	2.5744E-05	0.00023484	Eng:WT
Tnfrsf11a	21934	1.38033019	1.8494E-07	5.5111E-06	Eng:WT
Tnfrsf13b	57916	0.77315539	0.00289449	0.00793765	Eng:WT
Tnfrsf14	230979	0.98682031	0.00015572	0.00086935	Eng:WT
Tnfrsf1b	21938	1.06918921	0.00167188	0.00541544	Eng:WT
Tnfsf12	21944	1.20061326	0.00010553	0.00064621	Eng:WT
Tollip	54473	0.27600459	0.0074007	0.01705213	Eng:WT
Trem2	83433	1.48315363	0.00377492	0.00998455	Eng:WT
Trp53	22059	-0.5286375	0.00122164	0.0044396	Eng:WT
Tslp	53603	1.80240766	0.00505415	0.0124818	Eng:WT
Tyrobp	22177	1.31288337	0.00030339	0.00144271	Eng:WT
Vcam1	22329	1.10389234	0.00080549	0.00313089	Eng:WT
Zeb1	21417	0.6353865	0.00356883	0.00949565	Eng:WT

*Def = Deficient, Eng = Engrafted, OG = Other groups, WT = Wild-type

Supplementary Table 4: Gene Set Enrichment Analysis

ID	Description	NES	pvalue	qvalues	Comparison*
mmu04657	K_IL-17 signaling pathway	1.72	9.93E-06	1.10E-03	Def:OG
mmu04060	K_Cytokine-cytokine receptor interaction	1.46	4.72E-04	2.61E-02	Def:OG
mmu04061	K_Viral protein interaction with cytokine and cytokine receptor	1.58	8.86E-04	2.61E-02	Def:OG
mmu04062	K_Chemokine signaling pathway	1.59	9.46E-04	2.61E-02	Def:OG
mmu04668	K_TNF signaling pathway	1.60	1.46E-03	3.23E-02	Def:OG
GO:0071621	G_granulocyte chemotaxis	1.67	1.48E-05	1.12E-02	Def:OG
GO:0006935	G_chemotaxis	1.55	3.58E-05	1.12E-02	Def:OG
GO:0042330	G_taxis	1.55	3.58E-05	1.12E-02	Def:OG
GO:0050900	G_leukocyte migration	1.52	4.59E-05	1.12E-02	Def:OG
R-MMU-6798695	R_Neutrophil degranulation	1.59	7.74E-04	4.13E-02	Def:OG
R-MMU-162582	R_Signal Transduction	1.38	1.87E-03	4.13E-02	Def:OG
R-MMU-380108	R_Chemokine receptors bind chemokines	1.67	2.23E-03	4.13E-02	Def:OG
R-MMU-373076	R_Class A/1 (Rhodopsin-like receptors)	1.61	2.71E-03	4.13E-02	Def:OG
R-MMU-500792	R_GPCR ligand binding	1.60	2.85E-03	4.13E-02	Def:OG
R-MMU-372790	R_Signaling by GPCR	1.59	2.86E-03	4.13E-02	Def:OG
R-MMU-375276	R_Peptide ligand-binding receptors	1.63	3.82E-03	4.32E-02	Def:OG
R-MMU-418594	R_G alpha (i) signalling events	1.65	3.93E-03	4.32E-02	Def:OG
R-MMU-168249	R_Innate Immune System	1.38	4.28E-03	4.32E-02	Def:OG
MM3882	H__COMPLEMENT	1.61	2.51E-03	2.72E-02	Def:OG
MM3889	H__EPITHELIAL_MESENCHYMAL_TRANSITION	1.68	3.04E-03	2.72E-02	Def:OG
mmu04657	K_IL-17 signaling pathway	1.88	1.64E-05	1.71E-03	Def:WT
mmu04062	K_Chemokine signaling pathway	1.79	9.96E-05	3.46E-03	Def:WT
mmu04061	K_Viral protein interaction with cytokine and cytokine receptor	1.73	6.97E-04	1.45E-02	Def:WT
mmu04060	K_Cytokine-cytokine receptor interaction	1.56	9.30E-04	1.61E-02	Def:WT
mmu04668	K_TNF signaling pathway	1.68	3.03E-03	3.95E-02	Def:WT
GO:0071621	G_granulocyte chemotaxis	1.89	1.88E-06	2.85E-03	Def:WT
GO:1990266	G_neutrophil migration	1.87	3.84E-06	2.85E-03	Def:WT
GO:0006935	G_chemotaxis	1.72	4.30E-05	7.14E-03	Def:WT
GO:0042330	G_taxis	1.72	4.30E-05	7.14E-03	Def:WT
GO:0007281	G_germ cell development	-2.18	7.02E-05	1.01E-02	Def:WT
GO:0040011	G_locomotion	1.51	1.14E-04	1.50E-02	Def:WT
GO:0006928	G_movement of cell or subcellular component	1.51	1.24E-04	1.50E-02	Def:WT
GO:0009617	G_response to bacterium	1.59	1.69E-04	1.61E-02	Def:WT
GO:0051674	G_localization of cell	1.52	1.73E-04	1.61E-02	Def:WT
GO:0061844	G_antimicrobial humoral immune response mediated by antimicrobial peptide	1.90	2.08E-04	1.82E-02	Def:WT
GO:0006954	G_inflammatory response	1.49	2.30E-04	1.91E-02	Def:WT
GO:0055123	G_digestive system development	-2.04	3.10E-04	2.38E-02	Def:WT
GO:0019725	G_cellular homeostasis	1.70	3.42E-04	2.46E-02	Def:WT

ID	Description	NES	pvalue	qvalues	Comparison*
GO:0070887	G_cellular response to chemical stimulus	1.35	5.17E-04	3.27E-02	Def:WT
GO:0006873	G_cellular ion homeostasis	1.73	7.18E-04	4.10E-02	Def:WT
GO:0007204	G_positive regulation of cytosolic calcium ion concentration	1.76	7.19E-04	4.10E-02	Def:WT
GO:0048568	G_embryonic organ development	-1.88	7.26E-04	4.10E-02	Def:WT
GO:0009605	G_response to external stimulus	1.33	8.20E-04	4.35E-02	Def:WT
GO:0098771	G_inorganic ion homeostasis	1.70	9.95E-04	4.35E-02	Def:WT
GO:0070098	G_chemokine-mediated signaling pathway	1.84	1.00E-03	4.35E-02	Def:WT
GO:1990868	G_response to chemokine	1.84	1.00E-03	4.35E-02	Def:WT
GO:1990869	G_cellular response to chemokine	1.84	1.00E-03	4.35E-02	Def:WT
GO:0007548	G_sex differentiation	-1.88	1.02E-03	4.35E-02	Def:WT
R-MMU-380108	R_Chemokine receptors bind chemokines	1.88	4.81E-05	4.81E-03	Def:WT
R-MMU-372790	R_Signaling by GPCR	1.76	1.68E-04	6.65E-03	Def:WT
R-MMU-500792	R_GPCR ligand binding	1.77	2.04E-04	6.65E-03	Def:WT
R-MMU-418594	R_G alpha (i) signalling events	1.85	2.66E-04	6.65E-03	Def:WT
R-MMU-373076	R_Class A/1 (Rhodopsin-like receptors)	1.80	3.63E-04	6.87E-03	Def:WT
R-MMU-375276	R_Peptide ligand-binding receptors	1.84	4.31E-04	6.87E-03	Def:WT
R-MMU-388396	R_GPCR downstream signalling	1.80	4.81E-04	6.87E-03	Def:WT
R-MMU-6798695	R_Neutrophil degranulation	1.66	2.18E-03	2.72E-02	Def:WT
R-MMU-162582	R_Signal Transduction	1.47	3.31E-03	3.68E-02	Def:WT
R-MMU-168249	R_Innate Immune System	1.47	4.50E-03	4.50E-02	Def:WT
mmu04657	K_IL-17 signaling pathway	2.17	1.35E-09	1.44E-07	Def:Eng
mmu04621	K_NOD-like receptor signaling pathway	2.05	8.56E-06	3.03E-04	Def:Eng
mmu04064	K_NF-kappa B signaling pathway	1.98	8.25E-05	2.19E-03	Def:Eng
mmu04668	K_TNF signaling pathway	2.02	1.04E-04	2.21E-03	Def:Eng
mmu05417	K_Lipid and atherosclerosis	1.91	4.88E-04	7.41E-03	Def:Eng
mmu04061	K_Viral protein interaction with cytokine and cytokine receptor	1.87	2.88E-03	3.40E-02	Def:Eng
mmu04145	K_Phagosome	-1.81	3.54E-03	3.57E-02	Def:Eng
mmu04062	K_Chemokine signaling pathway	1.89	3.70E-03	3.57E-02	Def:Eng
GO:0071621	G_granulocyte chemotaxis	2.03	4.70E-06	4.15E-03	Def:Eng
GO:0097530	G_granulocyte migration	1.99	7.46E-06	4.15E-03	Def:Eng
GO:0006928	G_movement of cell or subcellular component	1.69	1.21E-05	4.15E-03	Def:Eng
GO:0040011	G_locomotion	1.69	1.29E-05	4.15E-03	Def:Eng
GO:0051674	G_localization of cell	1.69	2.54E-05	4.54E-03	Def:Eng
GO:0006935	G_chemotaxis	1.82	3.68E-04	3.95E-02	Def:Eng
GO:0042330	G_taxis	1.82	3.68E-04	3.95E-02	Def:Eng
GO:0009617	G_response to bacterium	1.71	4.31E-04	4.34E-02	Def:Eng
GO:0006954	G_inflammatory response	1.60	4.97E-04	4.65E-02	Def:Eng
GO:0002495	G_antigen processing and presentation of peptide antigen via MHC class II	-1.92	5.49E-04	4.65E-02	Def:Eng
GO:0002504	G_antigen processing and presentation of peptide or polysaccharide antigen via MHC class II	-1.92	5.49E-04	4.65E-02	Def:Eng

ID	Description	NES	pvalue	qvalues	Comparison*
MM3882	H__COMPLEMENT	2.00	1.62E-04	2.90E-03	Def:Eng
MM3889	H__EPITHELIAL_MESENCHYMAL_TRANSITI ON	1.93	9.50E-04	8.50E-03	Def:Eng
MM3860	H__TNFA_SIGNALING_VIA_NFKB	1.88	2.68E-03	1.60E-02	Def:Eng
mmu04062	K_Chemokine signaling pathway	1.91	3.44E-04	1.90E-02	Eng:WT
GO:0055123	G_digestive system development	-2.27	1.33E-05	2.07E-02	Eng:WT
GO:0097530	G_granulocyte migration	1.89	1.05E-04	3.10E-02	Eng:WT
GO:0043207	G_response to external biotic stimulus	1.52	1.13E-04	3.10E-02	Eng:WT
GO:0051707	G_response to other organism	1.52	1.13E-04	3.10E-02	Eng:WT
GO:0006955	G_immune response	1.49	1.19E-04	3.10E-02	Eng:WT
GO:0044419	G_interspecies interaction between organisms	1.45	2.44E-04	4.63E-02	Eng:WT
GO:0009607	G_response to biotic stimulus	1.50	2.67E-04	4.63E-02	Eng:WT
R-MMU-380108	R_Chemokine receptors bind chemokines	2.09	2.77E-05	2.72E-03	Eng:WT
R-MMU-375276	R_Peptide ligand-binding receptors	2.02	1.22E-04	4.64E-03	Eng:WT
R-MMU-372790	R_Signaling by GPCR	1.92	1.42E-04	4.64E-03	Eng:WT
R-MMU-500792	R_GPCR ligand binding	1.95	2.07E-04	4.83E-03	Eng:WT
R-MMU-373076	R_Class A/1 (Rhodopsin-like receptors)	1.99	2.79E-04	4.83E-03	Eng:WT
R-MMU-418594	R_G alpha (i) signalling events	2.00	2.96E-04	4.83E-03	Eng:WT
R-MMU-388396	R_GPCR downstream signalling	1.95	5.01E-04	7.00E-03	Eng:WT
R-MMU-168249	R_Innate Immune System	1.57	7.59E-04	9.28E-03	Eng:WT

*Def = Deficient, Eng = Engrafted, OG = Other groups, WT = Wild-type

Supplementary References

1. Rao, N.*, et al.* Engineering an Injectable Muscle-Specific Microenvironment for Improved Cell Delivery Using a Nanofibrous Extracellular Matrix Hydrogel. *ACS Nano* **11**, 3851-3859 (2017).

2. Wang, H.*, et al.* NanoStringDiff: a novel statistical method for differential expression analysis based on NanoString nCounter data. *Nucleic Acids Research* **44**, e151-e151 (2016).

3. R., K. pheatmap: Pretty heatmaps [Software]. (2015).
