# Supplementary material for: Immunomodulatory contribution of mast cells to the regenerative biomaterial microenvironment
Source: NPJ Regen Med. 2023 Sep 20;8:53. doi: 10.1038/s41536-023-00324-0 (PMC10511634; doi:10.1038/s41536-023-00324-0)
Supplement: Supplementary file 2 — Reporting Summary [file 41536_2023_324_MOESM2_ESM.pdf]

nature portfolio

Corresponding author(s): Karen Christman

Last updated by author(s): July 9, 2023

Reporting Summary

Nature Portfolio wishes to improve the reproducibility of the work that we publish. This form provides structure for consistency and transparency in reporting. For further information on Nature Portfolio policies, see our Editorial Policies and the Editorial Policy Checklist.

Statistics

For all statistical analyses, confirm that the following items are present in the figure legend, table legend, main text, or Methods section.

n/a	Confirmed	
☐	☒	The exact sample size (*n*) for each experimental group/condition, given as a discrete number and unit of measurement
☐	☒	A statement on whether measurements were taken from distinct samples or whether the same sample was measured repeatedly
☐	☒	The statistical test(s) used AND whether they are one- or two-sided<br>*Only common tests should be described solely by name; describe more complex techniques in the Methods section.*
☐	☒	A description of all covariates tested
☐	☒	A description of any assumptions or corrections, such as tests of normality and adjustment for multiple comparisons
☐	☒	A full description of the statistical parameters including central tendency (e.g. means) or other basic estimates (e.g. regression coefficient) AND variation (e.g. standard deviation) or associated estimates of uncertainty (e.g. confidence intervals)
☐	☒	For null hypothesis testing, the test statistic (e.g. *F*, *t*, *r*) with confidence intervals, effect sizes, degrees of freedom and *P* value noted<br>*Give P values as exact values whenever suitable.*
☒	☐	For Bayesian analysis, information on the choice of priors and Markov chain Monte Carlo settings
☒	☐	For hierarchical and complex designs, identification of the appropriate level for tests and full reporting of outcomes
☐	☒	Estimates of effect sizes (e.g. Cohen's *d*, Pearson's *r*), indicating how they were calculated

Our web collection on statistics for biologists contains articles on many of the points above.

Software and code

Policy information about availability of computer code

Data collection	Aperio: Scanner Console Version 102.0.0.65<br>Ariol: Software 4.0.0.53 (2012)<br>CFX Biorad 96: CFX Manager 3.1<br>Flow Core Diva Software 8.0.2
Data analysis	Image analysis was performed with custom scripts in MATLAB 2019a (https://doi.org/10.5281/zenodo.7196556). Statistics were performed in Prism 8. Flow cytometry data was analyzed in FlowJo v10.6.2. Nanostring analysis was performed with manufacturer nSolver Analysis Software 4.0 and custom R scripts under R version 4.04 utilizing publicly available R packages (https://doi.org/10.5281/zenodo.7190263).

For manuscripts utilizing custom algorithms or software that are central to the research but not yet described in published literature, software must be made available to editors and reviewers. We strongly encourage code deposition in a community repository (e.g. GitHub). See the Nature Portfolio guidelines for submitting code & software for further information.

Data

Policy information about availability of data

All manuscripts must include a data availability statement. This statement should provide the following information, where applicable:

- Accession codes, unique identifiers, or web links for publicly available datasets
- A description of any restrictions on data availability
- For clinical datasets or third party data, please ensure that the statement adheres to our policy

Raw Nanostring data files and normalized counts are available through GEO (GSE218383). Additional data may be requested from the authors.

Field-specific reporting

Please select the one below that is the best fit for your research. If you are not sure, read the appropriate sections before making your selection.

☒ Life sciences ☐ Behavioural & social sciences ☐ Ecological, evolutionary & environmental sciences

For a reference copy of the document with all sections, see nature.com/documents/nr-reporting-summary-flat.pdf

Life sciences study design

All studies must disclose on these points even when the disclosure is negative.

Sample size

Sample size was determined based on power analysis from low n pilot experiments up to n =3 for first iteration results. A power analysis was performed based on these initial results with a alpha value of 0.05 and a desired power of 0.90 to determine a suggested sample size for experiments. Nanostring samples utilized sample from PCR results with samples excluded if not enough RNA or randomly excluded to have n = 8/per group due to limited available sample input space per panel (12 per panel).

Data exclusions

For PCR results, samples were excluded if not enough RNA isolated for analysis. Nanostring samples utilized RNA sample from PCR results with samples randomly selected for exclusion to have n - 8/per group due to limited available sample input space per panel (12 per panel). Flow cytometry samples that lacked a defined cluster in LIVE/DEAD staining that indicated a low number of viable cells from isolation.

Replication

In vitro results were repeated at least once to confirm reproducibility. Following first experimental iterations (n = 2-3) that showed differences between groups, additional samples were collected across 2-3 experimental batches of animals for demonstrating consistency of results and reaching samples sizes as suggested by power analysis.

Randomization

For injection of decellularized versus non-decellularized materials, mice were randomized for receiving the two material injections. For single material experiments, order of injections across different mouse models were randomized and each procedure day included injections involving at least wild-type and deficient mice to avoid adjustments from procedural proficiency per day and per day variability influencing only a single experimental group.

Blinding

For image analysis, information indicating groups for analyses were replaced with randomized identifiers while analysis was being performed and decoded when grouping finalized results..

Reporting for specific materials, systems and methods

We require information from authors about some types of materials, experimental systems and methods used in many studies. Here, indicate whether each material, system or method listed is relevant to your study. If you are not sure if a list item applies to your research, read the appropriate section before selecting a response.

Materials & experimental systems

n/a	Involved in the study	
☐	☒	Antibodies
☒	☐	Eukaryotic cell lines
☒	☐	Palaeontology and archaeology
☐	☒	Animals and other organisms
☒	☐	Human research participants
☒	☐	Clinical data
☒	☐	Dual use research of concern

Methods

n/a	Involved in the study	
☒	☐	ChIP-seq
☐	☒	Flow cytometry
☒	☐	MRI-based neuroimaging

Antibodies

Antibodies used

Stain, Company, Catalog, Clone, Lot;
F4/80, eBioscience,14-4801-82, BM8, E04273-1601-85;
Ly-6G/Ly-6C, eBioscience, 14-5931-82, RB6-8C5, 2028665;
CD11c BV421, Biolegend, 117343, N418, B258584;
F4/80 BUV395, BD Biosciences, 565614, T45-2342, 9059561;
CD3 PerCp/Cy5.5, Biolegend, 100218, 17A2, B260626;
CD117 APC, Biolegend, 105812, 2B8, B255216;
FceRI PE, Biolegend, 134307, MAR-1, B278337;
CD19 APC-Cy7, Biolegend, 115530, 6D5, B290859;
CD4 APC, Biolegend, 100412, GK1.5, B280301;
CD8a AF488, Biolegend, 100723, 53-6.7, B254526;
CD206 (MMR) PE, Biolegend, 141705, C068C2, B280037;
CD86 BV785, Biolegend, 105043, GL-1, B269768;
FoxP3 BV421, Biolegend, 126419, MF-14, B285905;

Validation

All immunohistochemistry antibodies were validated for signal versus noise utilizing primary only, secondary only and isotype controls during titration optimization. Secondary and isotype controls were also performed per bulk batch round of staining slides. Flow cytometry antibodies were titrated along with being compared to isotype and fluorescence minus one controls during panel set-up, along with isotype control staining per bulk batch round of cell suspension staining.

Animals and other organisms

Policy information about studies involving animals; ARRIVE guidelines recommended for reporting animal research

Laboratory animals

Male and female homozygous mast cell-deficient mouse, w-sh mice: B6.Cg-KitW-sh/HNihrJaeBsmJ and wild-type C57BL6/J mice between 11-13 weeks of age.

Wild animals

Study did not involve wild samples.

Field-collected samples

Study did not involve field-collected samples.

Ethics oversight

All procedures in this study were performed in accordance with the guidelines established by the Committee on Animal Research at the University of California, San Diego and the Association for the Assessment and Accreditation of Laboratory Animal Care.

Note that full information on the approval of the study protocol must also be provided in the manuscript.

Flow Cytometry

Plots

Confirm that:

☒ The axis labels state the marker and fluorochrome used (e.g. CD4-FITC).

☒ The axis scales are clearly visible. Include numbers along axes only for bottom left plot of group (a 'group' is an analysis of identical markers).

☒ All plots are contour plots with outliers or pseudocolor plots.

☒ A numerical value for number of cells or percentage (with statistics) is provided.

Methodology

Sample preparation

Non-adhered bone marrow derived mast cells were directly collected by centrifugation, washed, stained and sorted. Pieces from multiple excised subcutaneous injections per animal were pooled and minced in ice-cold HBSS (Gibco) and enzymatically digested in a solution consisting of 1:1 solution of HBSS (calcium and magnesium supplemented) and 1% bovine serum albumin in PBS with 1 µM HEPES (Gibco), 300 U/mL collagenase type IV (Worthington Biochemical), 60 U/mL hyaluronidase (Sigma-Aldrich) and 10 U/mL DNase I (Sigma-Aldrich). Material in enzymatic digestion solution were incubated at 37ºC under mechanical agitation at 750 rpm on a thermomixer (Benchmark Scientific) for 45 minutes. Solutions were then kept in ice and FACs buffer consisting of 1% bovine serum albumin and 1mM EDTA in DPBS lacking calcium and magnesium added to inhibit further enzyme reaction. Digested tissue was filtered through a 100µm cell strainer. Cells were centrifuged at 400 rcf centrifugation at 4ºC and resuspended in HBSS. Cell suspension was stained with LIVE/DEADTM Fixable Aqua (ThermoFisher Scientific) for 10 minutes at 4ºC and excess dye was quenched with FACs buffer. Cells were fixed and permeabilized by BD Cytofix/CytopermTM Buffer (BD Biosciences) for 10 minutes and washed in BD Perm/WashTM Buffer (BD Biosciences). Cells were counted by hemocytometer and stained with antibody panels for immune cell subpopulations.

Instrument

Samples were analyzed on a BD FACSCanto(TM) II and BD LSRFortessa(TM) X-20 (BD Biosciences).

Software

Gating and flow data were processed in FlowJo v10.6.2.

Cell population abundance

Live cells following isolation of single cell suspension were designated based on a LIVE/DEAD stain gating. As the injected materials are acellular, a large percentage of the total cells isolated were infiltrating immune cells, specifically on average containing macrophages (~30-50%), dendritic cells (~5-15%), T cells (~1-4%), B cells (~0.1-2%), and mast cells (~0-0.05%) with variations based on timepoint and in individual samples. Individual data points for relative abundance of designated immune cell populations are displayed in the Supplementary Figures 7-11.

Gating strategy

Gating for positive and negative staining was set based on isotype and fluorescence minus one controls during panel set-up validation along with

☒ Tick this box to confirm that a figure exemplifying the gating strategy is provided in the Supplementary Information.
